# Disrupted minor intron splicing activates reductive carboxylation-mediated lipogenesis to drive metabolic dysfunction–associated steatotic liver disease progression

**DOI:** 10.1172/JCI186478

**Published:** 2025-03-18

**Authors:** Yinkun Fu, Xin Peng, Hongyong Song, Xiaoyun Li, Yang Zhi, Jieting Tang, Yifan Liu, Ding Chen, Wenyan Li, Jing Zhang, Jing Ma, Ming He, Yimin Mao, Xu-Yun Zhao

**Affiliations:** 1 Department of Biochemistry and Molecular Cell Biology, Shanghai Key Laboratory for Tumor Microenvironment and Inflammation, Key Laboratory of Cell Differentiation and Apoptosis of National Ministry of Education and; 2 Institute for Translational Medicine on Cell Fate and Disease, Shanghai Ninth People’s Hospital, Key Laboratory of Cell Differentiation and Apoptosis of National Ministry of Education, Department of Pathophysiology, Shanghai Jiao Tong University School of Medicine, Shanghai, China.; 3 Division of Gastroenterology and Hepatology, Renji Hospital, Shanghai Jiao Tong University School of Medicine; NHC Key Laboratory of Digestive Diseases, Shanghai Research Center of Fatty Liver Disease, Shanghai, China.; 4 Department of Endocrinology and Metabolism, Renji Hospital, Shanghai Jiao Tong University School of Medicine, Shanghai, China.; 5 Department of Pathology, Affiliated Hospital of Youjiang Medical University for Nationalities, Baise, China.

**Keywords:** Hepatology, Metabolism, Amino acid metabolism, Fibrosis, RNA processing

## Abstract

Aberrant RNA splicing is tightly linked to diseases, including metabolic dysfunction–associated steatotic liver disease (MASLD). In this study, we revealed that minor intron splicing, a unique and conserved RNA processing event, is largely disrupted upon the progression of metabolic dysfunction–associated steatohepatitis (MASH) in mice and humans. We demonstrated that deficiency of minor intron splicing in the liver induced MASH transition upon obesity-induced insulin resistance and LXR activation. Mechanistically, inactivation of minor intron splicing led to minor intron retention of *Insig1* and *Insig2*, resulting in premature termination of translation, which drove proteolytic activation of SREBP1c. This mechanism was conserved in patients with MASH. Notably, disrupted minor intron splicing activated glutamine reductive metabolism for de novo lipogenesis through induction of Idh1, which caused accumulation of ammonia in the liver, thereby initiating hepatic fibrosis upon LXR activation. Ammonia clearance or IDH1 inhibition blocked hepatic fibrogenesis and mitigated MASH progression. More importantly, overexpression of *Zrsr1* restored minor intron retention and ameliorated the development of MASH, indicating that dysfunctional minor intron splicing is an emerging pathogenic mechanism that drives MASH progression. Additionally, our results suggest that reductive carboxylation flux triggered by minor intron retention in hepatocytes serves as a crucial checkpoint and potential target for MASH therapy.

## Abstract 2

Inactivation of minor intron splicing caused by a decrease in Zrsr1 and Zrsr2 expression in the MASH liver leads to activation of the LXR-SREBP1c-IDH1 axis-mediated reductive carboxylation of glutamine for *de novo* lipogenesis and ammonia production via minor intron retention of *Insig1* and *Insig2*, therefore driving MASH progression.

## Graphical abstract

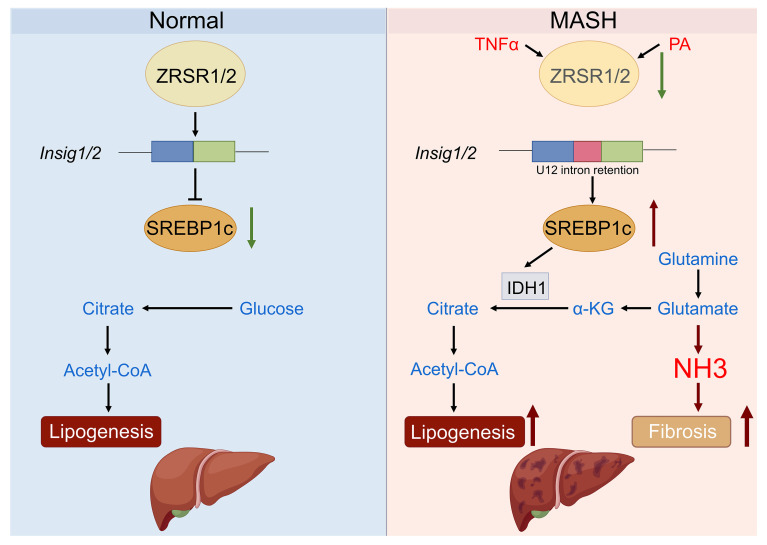

## Introduction

Metabolic dysfunction–associated steatotic liver disease (MASLD), previously termed nonalcoholic fatty liver disease (NAFLD), is a clinicopathological condition that is increasingly recognized as a component of the epidemic of obesity. It causes a spectrum of liver damage, ranging from simple fatty liver with uncomplicated steatosis to progressive metabolic dysfunction–associated steatohepatitis (MASH), which increases the risk of fibrosis and cirrhosis. It is estimated that more than 25% of the adult population worldwide has fatty liver, and approximately 5%–10% of these individuals further progress to MASH (1–3). MASLD is rapidly emerging as a leading etiology for chronic liver disease and a common indication for adult liver transplantation. Notably, the prevalence of MASLD is expected to increase worldwide along with the increasing incidence of obesity and type 2 diabetes mellitus (T2DM) due to the lack of effective pharmacotherapies (4, 5). An increase in hepatic steatosis is a hallmark of MASLD progression. More importantly, the pathogenic consequences of the MASH transition, such as hepatic fibrosis, cirrhosis and hepatocyte death, are largely implicated in the mortality and the prognosis of MASH and are ultimately irreversible (6–9). Therefore, blocking the MASH transition is a critical intervention for the pathogenesis of MASH.

RNA splicing is a fundamental biological process that is mediated by a special group of RNA-binding proteins that control the posttranscriptional processing of RNA and generate protein diversity (10, 11). However, abnormal RNA splicing has become a pathological factor in diverse diseases (12, 13). The splicing of introns depends on the terminal dinucleotides of the intron, which include GT-AG/GC-AG and AT-AC, referred to as major (U2-type) and minor (U12-type) introns, respectively. While major introns are universally present in the majority of genes, minor introns are rare and exist in only a small class of genes (<0.4% of all introns); however, they are highly conserved and function in various indispensable biological pathways (14). Recent studies revealed that dysregulation of the RNA splicing machinery is important for the development of MASLD (12, 15–17). The minor intron splicing factors zinc finger CCCH-type, RNA binding motif, and serine/arginine-rich 1/2 (*Zrsr1* and *Zrsr2*) are highly conserved paralogs that recognize a unique splice site of minor introns upon splicing (18). In humans, *ZRSR2* is the dominant minor intron splicing factor, since *ZRSR1* is considered a pseudogene, whereas in mice, both *Zrsr1* and *Zrsr2* are required for minor intron splicing (19). Mutations in *Zrsr1* or *Zrsr2* cause retention of most of the minor introns, which is associated with multiple diseases, including developmental disorders (19–22), neurodegeneration (23), and cancer (24). Although the pivotal role of major intron splicing in driving MASLD progression has been elucidated, how minor intron splicing is implicated in MASH pathogenesis needs further investigation.

De novo lipogenesis is a metabolic process that is closely associated with hepatic steatosis and MASH pathogenesis. In a conventional view, hepatocytes use acetyl-CoA generated by the glycolysis pathway for the de novo synthesis of fatty acids and lipids, whereas under certain conditions, such as hypoxia, glutamine is reductively carboxylated to citrate to produce lipogenic carbon (25). Notably, during cancer development, glutamine is frequently absorbed and reductively carboxylated to synthesize lipids to fulfill the demand for energy storage and cell proliferation (26). Targeting glutaminolysis has been proven to inhibit tumorigenesis by suppressing lipid synthesis (27). The relationship between MASH progression and aberrant stimulation of hepatic de novo lipogenesis has been firmly established in previous studies (28, 29). *Srebp1c* is a central regulator of the hepatic lipogenic gene program, which includes fatty acid synthase (*Fasn*) and stearoyl-CoA desaturase 1 (*Scd1*) (30, 31). Liver X receptor (*Lxr*), a nuclear hormone receptor, directly binds to the *Srebp1c* promoter and modulates *Srebp1c* expression. It is documented that LXR-mediated SREBP1c activity is highly stimulated during MASH progression (32). Treatment with the LXR agonist T0901317 (T1317) results in severe hypertriacylglycerolemia and hepatic triacylglycerol accumulation, whereas treatment with the LXR antagonist SR9238 blocks MASH progression (33). During the development of MASLD, an increased number of SREBP1c proteins are processed to their active forms in the Golgi apparatus by protease cleavage. Insulin-induced gene 1 and 2 (*Insig1* and *Insig2*) are ER-localized polytopic membrane proteins that bind SREBP cleavage–activating protein (SCAP) and prevent it from escorting SREBP1c to the Golgi apparatus for proteolytic processing (34–37). Loss of INSIG proteins is largely implicated in MASH and cancer progression (38, 39).

In this study, we revealed that minor intron splicing was profoundly inactivated during MASH development. Depletion of *Zrsr1* and *Zrsr2*, two essential minor intron splicing factors in the liver, directly induced hepatic steatosis and further promoted MASH progression upon obesity-induced insulin resistance and LXR activation. Deficiency of *Zrsr1* and *Zrsr2* in the liver triggered *Insig1* and *Insig2* minor intron retention for premature termination of translation, thereby constantly activating SREBP1c. Metabolomics and isotope-labeled metabolite flux analyses supported the notion that the dominant carbon source in the liver for lipogenesis switches to glutamine, and surprisingly, it undergoes SREBP1c-induced isocitrate dehydrogenase 1–mediated (IDH1-mediated) reductive carboxylation upon inactivation of minor intron splicing. This metabolic remodeling was critical in MASH progression due to the overflow of ammonia production in the liver. Clearance of ammonia or suppression of IDH1 activity reversed hepatic fibrosis and MASH progression. Herein, we demonstrated that minor intron splicing inactivation played an unexpected role in MASH progression by inducing SREBP1c nuclear form processing. Minor intron splicing deficiency augmented glutamine-derived lipogenic carbon by inducing reductive carboxylation flux for de novo lipogenesis. Thus, this LXR/SREBP1c/IDH1-mediated reductive carboxylation pathway may serve as a pivotal and unique checkpoint for MASH progression and a promising target for MASH therapy.

## Results

### Minor intron retention caused by reduction of Zrsr1 and Zrsr2 in the liver promotes MASH progression.

**Figure 1:**
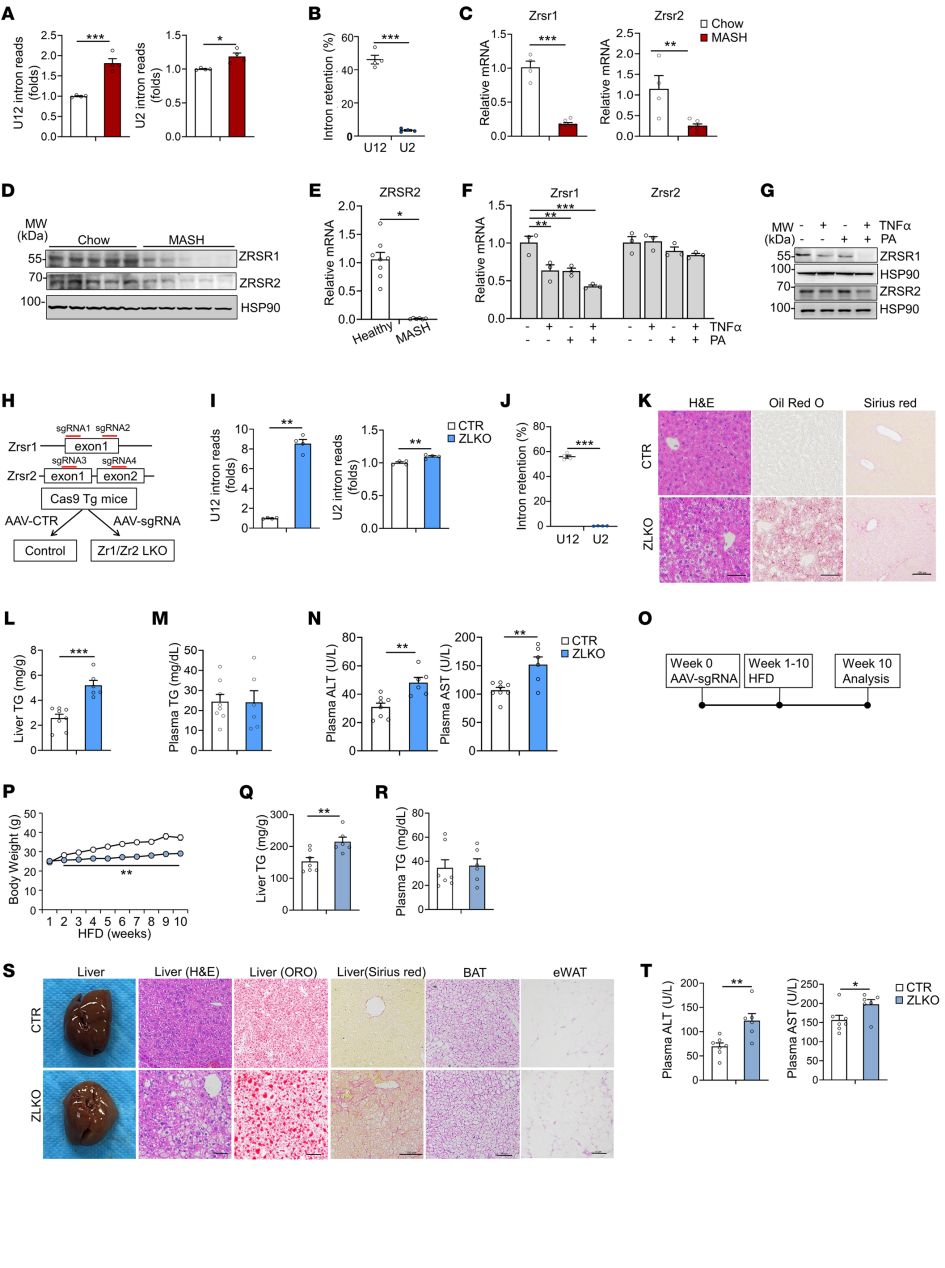
Inactivation of minor intron splicing caused by decrease in Zrsr1 and Zrsr2 expression in the liver induces MASH phenotype. (**A**) Fold change in minor (U12) and major (U2) intron reads of livers of mice after chow (*n* = 4) and CDA-HFD (MASH diet, *n* = 4) feeding for 6 months. (**B**) Percentages of U12 and U2 retained introns. (**C** and **D**) qPCR (**C**) and immunoblotting (**D**) analyses of Zrsr1 and Zrsr2 expression in livers of mice fed chow diet (*n* = 4) or MASH diet (*n* = 7). (**E**) qPCR analysis of ZRSR2 expression in liver samples from healthy donors as controls (*n* = 8) and patients with MASH (*n* = 6). (**F** and **G**) qPCR (**F**) and immunoblotting (**G**) analysis of Zrsr1 and Zrsr2 expression in primary hepatocytes (*n* = 3) treated with vehicle, 50 μM TNF-α, or 0.1 mM PA alone or in combination for 24 hours. (**H**) Schematic diagram of the strategy used to generate Zrsr1 and Zrsr2 double-deficient mice via CRISPR/CAS9. (**I**) Fold change in U12 and U2 intron reads of livers of AAV-control– (CTR, *n* = 4) and Zrsr1- and Zrsr2-sgRNA–injected Cas9-Tg mice (ZLKO, *n* = 4) on chow-diet feeding. (**J**) Percentages of U12 and U2 retained introns. (**K**) H&E, Oil Red O, and Sirius red staining (scale bars: 100 μm). (**L** and **M**) Liver (**L**) and plasma (**M**) triglyceride (TG) content in CTR (*n* = 8) and ZLKO mice (*n* = 6). (**N**) Plasma ALT and AST levels. (**O**) Diagram of the study design. (**P**) Body weight curves of CTR (*n* = 7) and ZLKO (*n* = 6) mice fed HFD for 10 weeks. (**Q** and **R**) Liver (**Q**) and plasma (**R**) TG content. (**S**) General morphology, H&E staining, Oil Red O staining, and Sirius red staining of liver tissue and H&E staining of fat tissue (scale bars: 100 μm). (**T**) Plasma ALT and AST levels. Data are presented as mean ± SEM. *P < 0.05, **P < 0.01, ***P < 0.001, by 2-tailed unpaired Student’s *t* test (**A**–**C**, **E**, **I**, **J**, **L**–**N**, **Q**, **R**, and **T**), by 2-way ANOVA with multiple comparisons (**P**), by 1-way ANOVA with Dunnett’s test (**F**).

The deficiency of RNA splicing is tightly linked to MASLD progression. However, whether minor intron splicing is disrupted in the MASH liver is unclear. Here, we performed RNA-Seq analyses of livers from mice fed chow diet and CDA-HFD (choline-deficient, l-amino acid–defined high-fat diet; also called MASH diet) and compared the occurrence rates of minor (U12) and major (U2) intron retention in the liver according to The Intron Annotation and Orthology Database (IAOD) (40). The results revealed that the fold change in amounts of both minor/U12 intron and major/U2 intron reads significantly increased in mouse livers after MASH diet feeding (Figure 1A). Interestingly, among the 657 minor introns found within 606 recognized genes that contain minor introns, a substantial number (46%) were retained. In contrast, of the 211,005 major introns present in 32,396 known genes with major introns, only 3.5% were retained, suggesting that minor intron retention is a common RNA splicing deficiency event in the MASH liver (Figure 1B). The dysregulation of minor intron splicing factors leads to minor intron retention. In our analysis of the minor intron splicing factors and small nuclear RNAs (snRNAs) in livers of MASH diet–fed mice, we found expression of *Zrsr1* and *Zrsr2* — which recognize the consensus splice site of the minor intron — to be substantially reduced (Figure 1, C and D and Supplemental Figure 1A; supplemental material available online with this article; https://doi.org/10.1172/JCI186478DS1). In addition, an RNA-Seq dataset (Gene Expression Omnibus database [GEO], GSE126848) of liver samples from healthy donors and individuals with MASH, as well as quantitative PCR (qPCR) analyses of liver biopsy samples collected from MASH patients, further confirmed that expression of *ZRSR2*, the predominant minor intron splicing factor in humans, was suppressed in patients with MASH (Figure 1E, Supplemental Figure 1B, and Supplemental Table 1). Excess fatty acid accumulation and severe inflammation in the liver facilitated MASH progression. Notably, treating primary hepatocytes with palmitic acid (PA) in combination with the inflammatory TNF-α significantly downregulated *Zrsr1* expression and slightly decreased *Zrsr2* expression (Figure 1, F and G). These results indicate that downregulation of *Zrsr1* and *Zrsr2* in the MASH liver may cause minor intron retention.

To investigate the consequences of the reduction in *Zrsr1* and *Zrsr2*, we took advantage of CRISPR/Cas9 editing strategy to generate a *Zrsr1* and *Zrsr2* double-deficient mouse model (ZLKO) via injection of adeno-associated virus (AAV), which carried 2 sgRNA pairs targeting *Zrsr1* and *Zrsr2*, as indicated, into a *Cas9*-transgenic mouse line (Figure 1H). The ZLKO mouse model offers a distinct advantage by effectively circumventing the potential compensatory effects that might occur when either *Zrsr1* or *Zrsr2* is depleted individually, as previously reported (41). After AAV-sgRNA virus injection, *Zrsr1* and *Zrsr2* were both largely depleted in the liver but not in other tissues. Additionally, inactivation of *Zrsr1* and *Zrsr2* was highly restricted to hepatocytes (Supplemental Figure 1, C–H). To verify whether minor intron splicing was compromised by *Zrsr1* and *Zrsr2* depletion, we analyzed RNA-Seq data from livers of ZLKO and control mice. The fold change in amounts of minor (U12) intron reads significantly increased, whereas that of major (U2) intron reads slightly increased in livers of ZLKO mice (Figure 1I). Among the 657 minor introns in 606 minor intron–containing genes, 56% were retained, whereas only 0.3% of the 211,005 major introns in 32,396 known major intron–containing genes showed retention, indicating that *Zrsr1* and *Zrsr2* abrogation mainly led to minor intron retention (Figure 1J and Supplemental Table 2). We subsequently analyzed the metabolic phenotype of the ZLKO mice. Compared with those of control mice, the livers of ZLKO mice were heavier and contained more lipid droplets, as shown by H&E and Oil Red O staining, while body weights of the mice were equivalent (Figure 1K and Supplemental Figure 2A). Then, we measured liver and plasma triglyceride and cholesterol content in control and ZLKO mice. Liver triglyceride and cholesterol levels in the ZLKO mice were significantly elevated, but plasma triglyceride and cholesterol levels in these mice remained unaltered (Figure 1, L and M, and Supplemental Figure 2B). Elevated hepatic triglyceride accumulation may be driven by insulin resistance. Therefore, we measured glucose content and insulin sensitivity of the control and ZLKO mice. Although the ad libitum glucose level was unchanged in the ZLKO mice, they exhibited slightly enhanced insulin sensitivity, as indicated by glucose tolerance test (GTT), and their insulin level was slightly lower (Supplemental Figure 2, C–E). These results indicate that the loss of *Zrsr1* and *Zrsr2* directly led to a hepatic steatosis phenotype independent of insulin resistance.

Onset of hepatic steatosis is a crucial risk factor in the advancement of MASLD. Notably, Sirius red staining and alanine aminotransferase (ALT) and aspartate aminotransferase (AST) measurements indicated that hepatic fibrogenesis and injury were initiated in the livers of ZLKO mice (Figure 1, K and N). Insulin resistance is a well-known driver of MASLD progression. High-fat diet–induced (HFD-induced) obesity impairs glucose homeostasis and leads to insulin resistance. To further explore the role of minor intron splicing in obesity-induced insulin resistance, we fed ZLKO and control mice a HFD for 10 weeks (Figure 1O). The ZLKO mice gained less weight than the control mice (Figure 1P) but had heavier livers (Supplemental Figure 3A); and liver triglyceride content was largely elevated, while plasma triglyceride, cholesterol, nonesterified fatty acid (NEFA), and β-hydroxybutyrate levels were not affected (Figure 1, Q and R, and Supplemental Figure 3, B–D). The food intake of the ZLKO mice during HFD feeding was not affected (Supplemental Figure 3E). Additionally, plasma glucose and insulin levels were not altered in the ZLKO mice (Supplemental Figure 3, F and G). Moreover, results of the GTT and insulin tolerance test (ITT) also revealed no differences between the control and ZLKO mice after HFD feeding (Supplemental Figure 3H), suggesting that the control and ZLKO mice had similar degrees of insulin resistance during diet-induced obesity. Liver histology and Oil Red O staining revealed more severe hepatic steatosis in the livers of ZLKO mice after HFD feeding than in those of control mice (Figure 1S). Although brown adipose tissue (BAT) histology and thermogenesis-related gene expression did not differ, epididymal white adipose tissue (eWAT) presented more multilocular structures and increased expression of thermogenic genes, indicating that more beige fat formed in ZLKO mice upon HFD feeding (Figure 1S and Supplemental Figure 3, I and J). This phenotype was strongly associated with restricted body weight gain in HFD-fed ZLKO mice (Figure 1P). In contrast, the ZLKO mice fed a low-fat diet had no difference in body weight gain (Supplemental Figure 3K), while thermogenic gene expression was unchanged in both BAT and eWAT, suggesting that beige fat formation in the eWAT of ZLKO mice was a unique event during obesity development (Supplemental Figure 3, L and M). Intriguingly, although the ZLKO mice were leaner after HFD feeding, the MASH phenotype — including liver collagen deposition and the degree of injury — was significantly augmented, as revealed by Sirius red staining and ALT and AST levels (Figure 1, S and T). These results imply that minor intron retention leads to accelerated MASH progression in obesity-induced insulin resistance conditions.

### Minor intron retention activates SREBP1c-mediated de novo lipogenesis, which results in the progression of MASH upon LXR activation.

**Figure 2:**
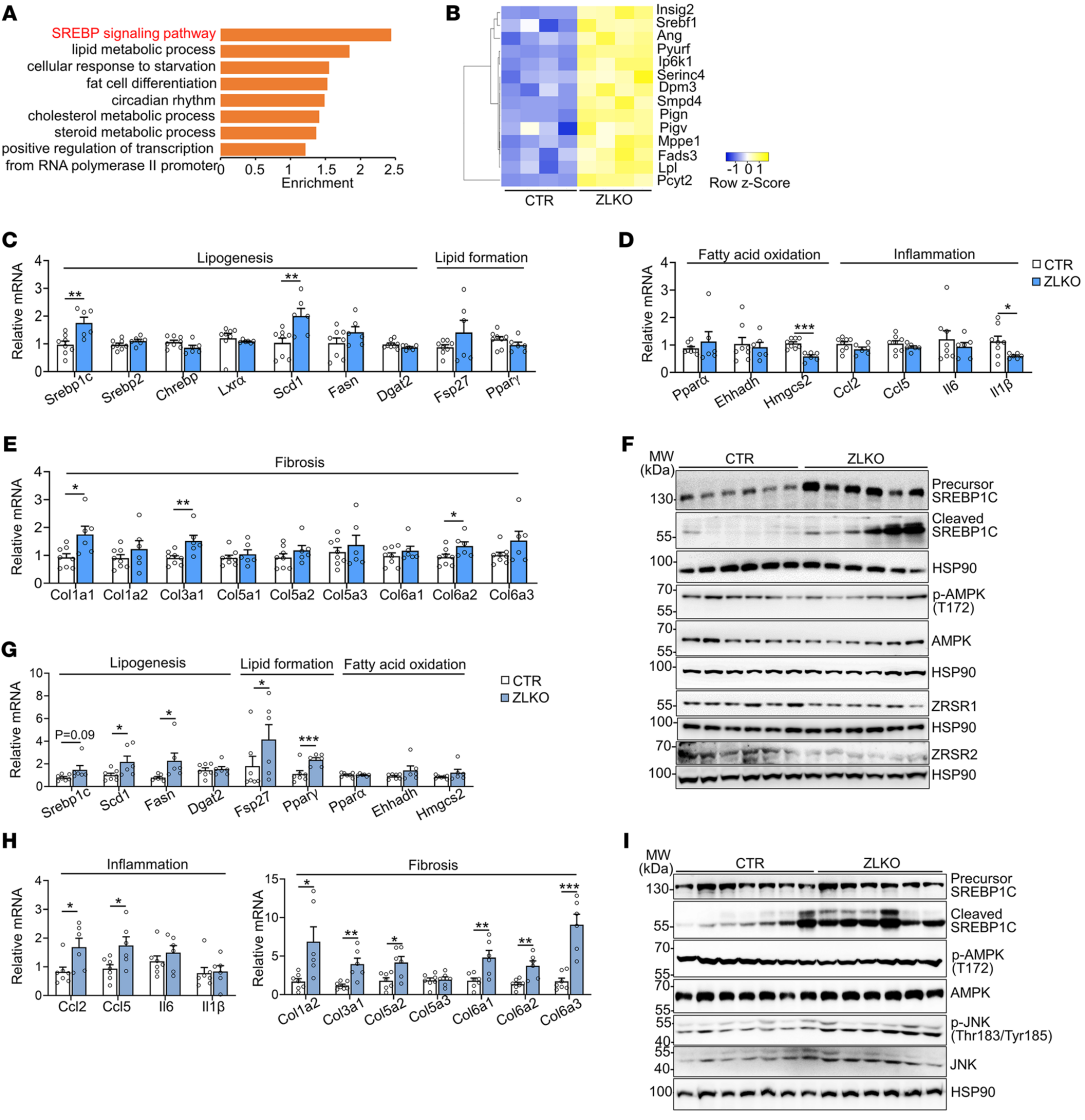
Minor intron splicing deficiency activates SREBP1c-mediated de novo lipogenesis. (**A**) Gene Ontology analysis showing enriched pathways associated with genes upregulated in the livers of ZLKO mice (*n* = 4) compared with those in CTR mice (*n* = 4) on chow-diet feeding. (**B**) Heatmap showing a set of lipogenic genes whose expression is upregulated in the livers of ZLKO mice on chow-diet feeding. (**C**–**E**) qPCR analysis of hepatic genes involved in lipogenesis and lipid formation (**C**), fatty acid oxidation and inflammation (**D**), and fibrosis (**E**) in CTR (*n* = 8) and ZLKO (*n* = 6) mice. (**F**) Immunoblotting of liver lysates from CTR and ZLKO mice. (**G** and **H**) qPCR analysis of hepatic genes involved in lipid metabolism (**G**) and inflammation and fibrosis (**H**) in CTR (*n* = 7) and ZLKO (*n* = 6) mice after HFD feeding. (**I**) Immunoblotting of liver lysates from CTR and ZLKO mice after HFD feeding. Data are presented as mean ± SEM. *P < 0.05, **P < 0.01, ***P < 0.001 by 2-tailed unpaired Student’s *t* test (**C**–**E**, **G**, and **H**).

To explore the potential pathways regulated by minor intron splicing deficiency, we analyzed RNA-Seq data from the livers of control and ZLKO mice. Overall, depletion of *Zrsr1* and *Zrsr2* led to more genes being upregulated than downregulated (Supplemental Figure 4A). Then, we clustered genes upregulated or downregulated more than 1.4-fold via DAVID clustering analysis (https://david.ncifcrf.gov/). The results showed that among the upregulated genes, there was drastic enrichment of genes in the SREBP signaling pathway, while the downregulated genes were clustered into lipid catabolism–associated pathways (Figure 2A and Supplemental Figure 4B). Moreover, expression of a gene set that regulates the de novo lipogenesis pathway was also greatly increased (Figure 2B). qPCR analyses revealed that expression of the lipogenic genes *Srebp1c* and *Scd1* was increased in the livers of ZLKO mice, while expression of genes regulating lipid formation did not change (Figure 2C). Interestingly, expression of genes involved in fatty acid oxidation and inflammation was slightly downregulated, whereas expression of genes linked to fibrosis was stimulated, suggesting that induction of fibrosis in the livers of ZLKO mice may be uncoupled from the inflammatory state (Figure 2, D and E). Immunoblotting revealed that *Srebp1c* expression and cleavage were augmented after depletion of *Zrsr1* and *Zrsr2*. Phosphorylation and total levels of AMPK, which regulates de novo lipogenesis, were not affected (Figure 2F and Supplemental Figure 4C). Consistent with this finding, after HFD feeding, expression of the upregulated lipogenic genes *Scd1* and *Fasn* and of the lipid formation genes fat-specific protein 27 (*Fsp27*) and *Pparγ* further increased, together with an increase in inflammatory- and fibrogenesis-related genes in ZLKO mice (Figure 2, G and H). Western blot analysis revealed that precursor and cleaved SREBP1c levels were increased in obese ZLKO mice. Since obesity increases immune cell infiltration in the liver, which elevates JNK signaling, we further measured phosphorylation of JNK. Western blot analysis revealed that expression of phosphorylated JNK was increased, further suggesting that minor intron splicing deficiency triggered hepatic inflammation and exacerbated fibrosis during obesity development. Levels of phosphorylated and total AMPK were unchanged, which was consistent with the results for the chow-fed group (Figure 2I and Supplemental Figure 4D).

**Figure 3:**
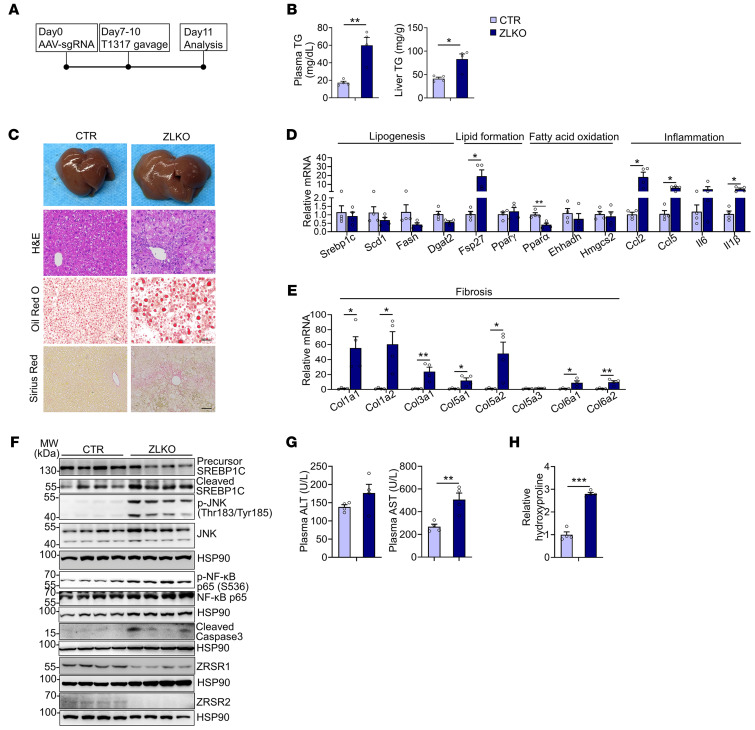
Minor intron splicing deficiency–induced activation of SREBP1c-mediated lipogenesis facilitates MASH progression under LXR activation. (**A**) Diagram of the study design. (**B**) Plasma (left) and liver (right) TG content of CTR (*n* = 4) and ZLKO (*n* = 4) mice receiving oral gavage of 25 mg/kg/d T1317 for 4 days. (**C**) General morphology, H&E staining, Oil Red O staining, and Sirius red staining (scale bars: 100 μm). (**D** and **E**) qPCR analysis of hepatic genes involved in lipid metabolism and inflammation (**D**) and fibrosis (**E**). (**F**) Immunoblotting of liver lysates from CTR and ZLKO mice. (**G** and **H**) Plasma ALT and AST levels (**G**) and liver hydroxyproline content (**H**). Data are presented as mean ± SEM. *P < 0.05, **P < 0.01, ***P < 0.001 by 2-tailed unpaired Student’s *t* test (**B**, **D**, **E**, **G**, and **H**).

The nuclear receptor LXRα/β binds retinoid X receptor α/β (RXRα/β) to form a heterodimer that transcriptionally induces *Srebp1c* expression. To investigate whether minor intron splicing directly modulates SREBP1c activity, we gavaged control and ZLKO mice with SR9238, an LXR antagonist, to suppress LXR activity (Supplemental Figure 5A). SR9238 treatment in WT mice significantly decreased expression of hepatic lipogenic genes (Supplemental Figure 5B). Conversely, in ZLKO mice, despite treatment with SR9238, levels of liver triglycerides and cholesterol remained significantly higher than those of control-treated mice (Supplemental Figure 5, C–F). In addition, although upregulation of *Srebp1c* mRNA in the livers of ZLKO mice was largely suppressed, expression of cleaved SREBP1c was still increased in ZLKO mice, which suggests that minor intron splicing may directly control SREBP1c nuclear form processing. This notion is further supported by induction of the SREBP1c target gene *Scd1* and the lipid marker *Fsp27* (Supplemental Figure 5, G and H). Aberrant activation of LXR/RXR complex activity during obesity is emerging as a vital cause of MASLD and facilitates MASH progression. To investigate whether minor intron splicing plays an important role in LXR/SREBP1c-mediated MASLD progression, we gavaged the LXR agonist T1317 in control and ZLKO mice (Figure 3A). In WT mice, T1317 treatment highly induced expression of hepatic lipogenic genes (Supplemental Figure 5I). Of note, T1317-treated ZLKO mice exhibited more severe liver steatosis, as well as elevated liver weights and plasma and hepatic triglyceride and cholesterol levels (Figure 3B and Supplemental Figure 5, J and K). Notably, the livers of the ZLKO mice became fibrotic after T1317 treatment, as shown by liver morphology and Sirius red staining (Figure 3C). Gene expression analyses revealed that inflammatory genes such as *Ccl2*, *Ccl5*, and *Il1β* and fibrotic genes such as collagen 1a1 (*Col1a1*), *Col1a2*, and *Col3a1* were dramatically upregulated. Expression of the lipogenic genes *Srebp1c*, *Fasn*, and *Scd1* was not further induced, probably due to the increase in inflammation in the liver, which suppressed the lipogenic program. However, expression of the lipid marker *Fsp27* was increased, which was associated with enhanced lipid deposition in the livers of ZLKO mice (Figure 3, C–E). Western blot analyses indicated that expression of precursor SREBP1c was equivalent but that the active form of SREBP1c was more abundant in ZLKO mice after T1317 treatment. Phosphorylation of the immune-associated NF-κB P65 subunit and activation of JNK were highly induced, suggesting that inflammation was extensively triggered in the liver. Additionally, the increase in cleaved caspase-3 indicated that apoptosis was induced in the ZLKO mouse liver after T1317 treatment (Figure 3F). The increase in inflammation and fibrosis in the *Zrsr1* and *Zrsr2* double-deficient livers led to liver damage and the MASH phenotype, which was supported by an increase in ALT and AST levels and hydroxyproline deposits in the livers of these mice (Figure 3, G and H). These results indicate that inactivation of minor intron splicing directly triggered SREBP1c processing, which promoted hepatic lipid accumulation. More importantly, the MASH phenotype was induced in the liver with minor intron retention upon LXR activation.

### Minor intron retention of the Insig1 and Insig2 genes promotes SREBP1c proteolytic activation.

**Figure 4:**
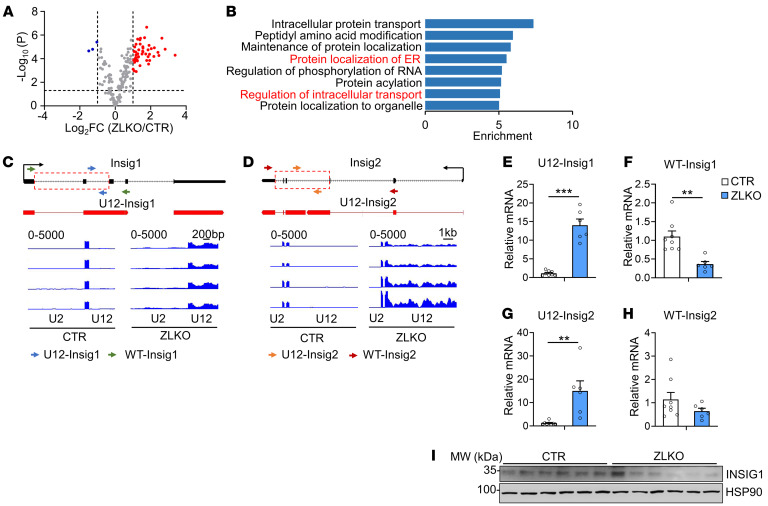
Disruption of minor intron splicing leads to *Insig1* and *Insig2* minor intron retention. (**A**) Volcano plot showing the upregulated (red) and downregulated (blue) minor intron retention genes in the livers of ZLKO mice (*n* = 4) compared with those of CTR mice (*n* = 4) on chow-diet feeding. FC, fold change. (**B**) Gene Ontology analysis showing pathways enriched in minor intron retention genes. (**C** and **D**) Genome Browser visualization showing peaks corresponding to the U12 and U2 introns in Insig1 and Insig2 in the livers of CTR (*n* = 4) and ZLKO (*n* = 4) mice. (**E**–**H**) qPCR analysis of U12 intron expression of Insig1 and Insig2 (**E** and **G**) and mRNA level of WT Insig1 and Insig2 (**F** and **H**) in the livers of CTR (*n* = 8) and ZLKO (*n* = 6) mice. The primers used are shown in **C** and **D**. (**I**) Immunoblotting of liver lysates from CTR and ZLKO mice.

**Figure 5:**
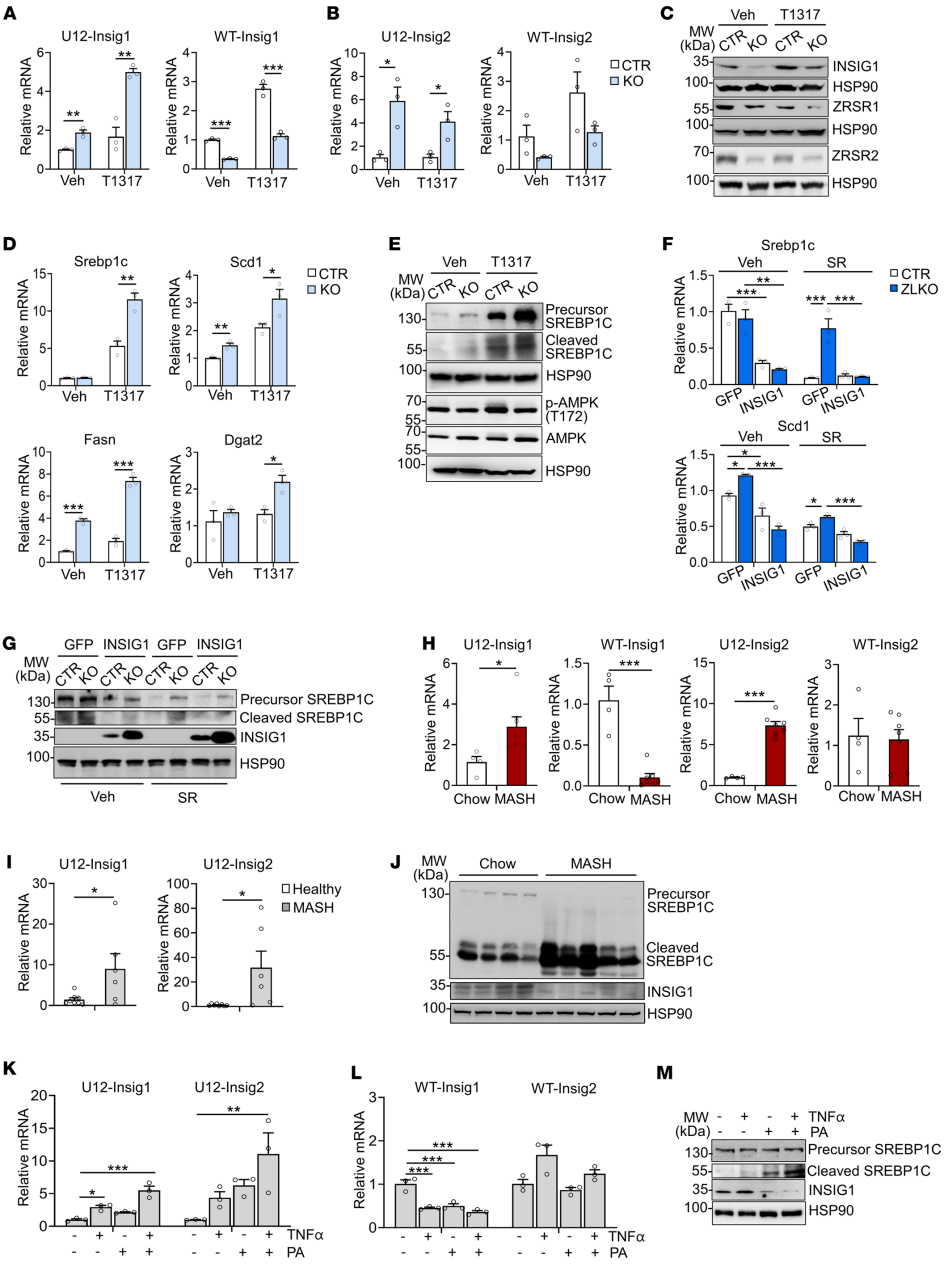
Minor intron retention of *Insig1* and *Insig2* mediates proteolytic activation of SREBP1c. (**A** and **B**) qPCR analysis of U12 intron and WT mRNA expression of Insig1 (**A**) and Insig2 (**B**) in control AML12 cells (CTR, *n* = 3) and AML12 cells with CRISPR/CAS9-mediated Zrsr1 and Zrsr2 knockout (KO, *n* = 3) treated with vehicle (Veh) or T1317 (5 μM) for 24 hours. (**C**) Immunoblotting of lysates from CTR and KO AML12 cells. (**D**) qPCR analysis of lipogenic genes in CTR and KO AML12 cells. (**E**) Immunoblotting of lysates from the cells in **D**. (**F**) Primary hepatocytes (*n* = 3) were isolated from the livers of CTR and ZLKO mice and infected with GFP and Insig1 adenoviruses. Expression of lipogenic genes was measured by qPCR after vehicle (Veh) or SR9238 (SR, 10 μM) treatment for 24 hours. (**G**) Immunoblotting of lysates from the cells in **F**. (**H**) qPCR analysis of U12 introns and WT mRNA expression of Insig1 and Insig2 in the livers of mice fed chow (*n* = 4) or MASH diet (*n* = 7) for 6 months. (**I**) qPCR analysis of U12 introns in Insig1 and Insig2 in liver samples from healthy donors (*n* = 8) and patients with MASH (*n* = 6). (**J**) Immunoblot analysis of liver lysates from mice fed chow or MASH diet. (**K** and **L**) qPCR analysis of U12 intron (**K**) and WT mRNA expression (**L**) of Insig1 and Insig2 in primary hepatocytes (*n* = 3) treated with vehicle, 50 μM TNF-α, or 0.1 mM PA alone or in combination for 24 hours. (**M**) Immunoblotting of cell lysates in **K** and **L**. Data are presented as mean ± SEM. *P < 0.05, **P < 0.01, ***P < 0.001 by 2-tailed unpaired Student’s *t* test (**A**, **B**, **D**, **H**, and **I**) and by 2-way ANOVA with multiple comparisons (**F**), by 1-way ANOVA with Dunnett’s test (**K** and **L**).

To explore the mechanism by which disrupted minor intron splicing activated SREBP1c processing, we further analyzed the expression and function of minor intron–retained genes via RNA-Seq. Expression of most these genes was upregulated, and in function they were strongly associated with protein localization of the endoplasmic reticulum (ER) and intracellular protein trafficking (Figure 4, A and B). Among them, Insig1 and Insig2 are classic inhibitors of de novo lipogenesis that function by anchoring the premature SREBP1c protein in the ER. We speculate that the minor intron retention of *Insig1* and *Insig2* may affect SREBP1c proteolytic activation. Through the UCSC Genome Browser, we revealed that there were increased peaks in the minor intron region of the *Insig1* and *Insig2* genes in ZLKO mice but not in adjacent major introns. The StringTie program reconstituted new transcripts of *Insig1* and *Insig2*, which were generated due to the retention of minor introns (Figure 4, C and D, and Supplemental Figure 6A). Furthermore, we verified that expression of minor introns in *Insig1* and *Insig2* was substantially increased in ZLKO mice, while expression of WT *Insig1* was decreased (Figure 4, E–H). The minor introns retained in the *Insig1* and *Insig2* transcripts contain alternative stop codons for premature termination of translation (Supplemental Figure 6A). Western blot analysis revealed that expression of *Insig1* decreased upon *Zrsr1* and *Zrsr2* depletion (Figure 4I). To investigate whether deficiency of minor intron splicing cell-autonomously mediated hepatic de novo lipogenesis through *Insig1* and *Insig2* minor intron retention, we substantially depleted *Zrsr1* and *Zrsr2* in an AML12 normal hepatic mouse cell line and primary hepatocytes via the CRISPR/Cas9 editing strategy. *Zrsr1* and *Zrsr2* sgRNA- and *Cas9*-transduced AML12 cells and primary hepatocytes (KO) presented increased *Insig1* and *Insig2* minor intron signals and decreased WT *Insig1* mRNA and protein expression (Figure 5, A–C, and Supplemental Figure 6B), while expression of the de novo lipogenic genes *Srebp1c*, *Fasn*, and *Scd1* was induced, as was SREBP1c cleavage. This effect was further amplified by LXR activation (Figure 5, D and E, and Supplemental Figure 6C). In addition, no significant change in the activation of AMPK was observed (Figure 5E). Notably, adenoviral overexpression of *Insig1* reversed *Zrsr1* and *Zrsr2* double deficiency–induced SREBP1c activation under both basal and LXR antagonist–treated conditions, indicating that the minor intron retention of *Insig* genes was the main factor causing SREBP1c processing in ZLKO mice (Figure 5, F and G). We next explored whether decreased *Zrsr1* and *Zrsr2* expression in the MASH stage further drives the minor intron retention of *Insig1* and *Insig2*. We measured expression of minor introns of *Insig1* and *Insig2* in the livers of mice fed a MASH diet and in patients with MASH. The results showed that minor intron expression of *Insig1* and *Insig2* was largely elevated in mice and humans at the MASH stage, while expression of WT Insig1 was reduced (Figure 5, H and I). Western blot analysis confirmed that INSIG1 protein expression was decreased and that SREBP1c processing was increased in the livers of mice at the MASH stage (Figure 5J). Notably, primary hepatocytes treated with PA and TNF-α alone presented increased minor intron expression of *Insig1* and *Insig2*, which was induced to a greater degree upon PA and TNF-α treatment in combination, while expression of WT Insig1 mRNA and protein was suppressed by PA and TNF-α treatment alone or in combination. Consequently, SREBP1c cleavage was increased (Figure 5, K–M). Taken together, these results provide evidence that hepatic minor intron retention of the *Insig1* and *Insig2* genes upon *Zrsr1* and *Zrsr2* depletion and at the MASH stage led to SREBP1c proteolytic activation.

### Dysfunction of minor intron splicing induces glutamine reductive carboxylation flux for de novo lipogenesis by activating IDH1.

**Figure 6:**
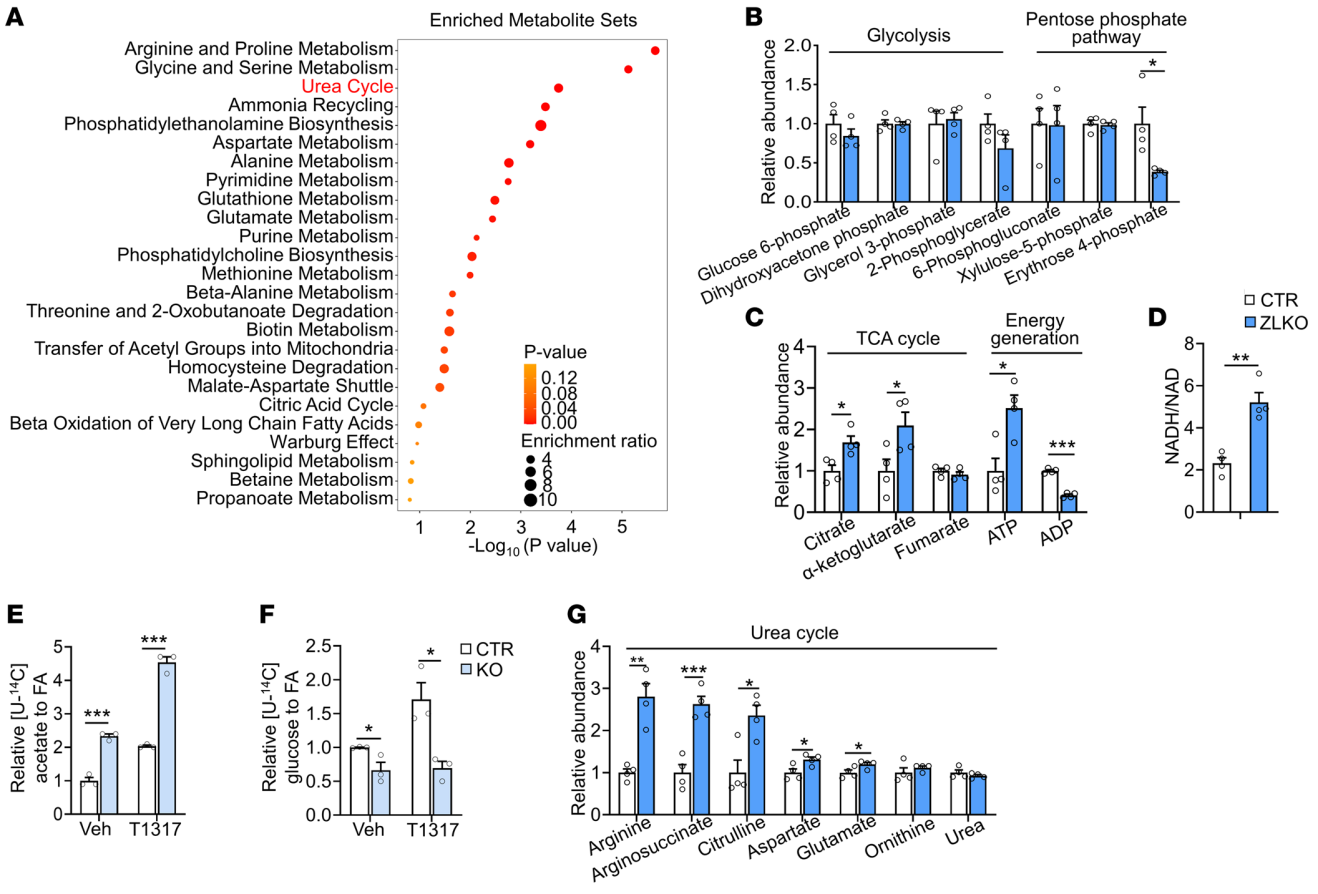
Dysfunction of minor intron splicing activates amino acid catabolism. (**A**) Clustering analysis performed by MetaboAnalyst showing the enriched metabolic pathways associated with the upregulated metabolites in livers of ZLKO mice (*n* = 4) compared with those of CTR mice (*n* = 4) after oral gavage administration of T1317 (25 mg/kg/d) for 4 days. (**B** and **C**) Levels of metabolites involved in glycolysis, the pentose phosphate pathway (**B**), the TCA cycle, and energy generation (**C**) were measured via metabolomics. (**D**) NADH and NAD levels were measured, and the NADH-to-NAD ratio was calculated. (**E** and **F**) Incorporation of [U-^14^C]acetate (**E**) and [U-^14^C]glucose (**F**) into lipids was measured in CTR and KO AML12 cells (*n* = 3) after vehicle (Veh) or T1317 (5 μM) treatment for 24 hours. (**G**) Metabolites involved in the urea cycle pathway were measured via metabolomics. Data are presented as mean ± SEM. **P < 0.01, ***P < 0.001 by 2-tailed unpaired Student’s *t* test (**B**–**G**).

**Figure 7:**
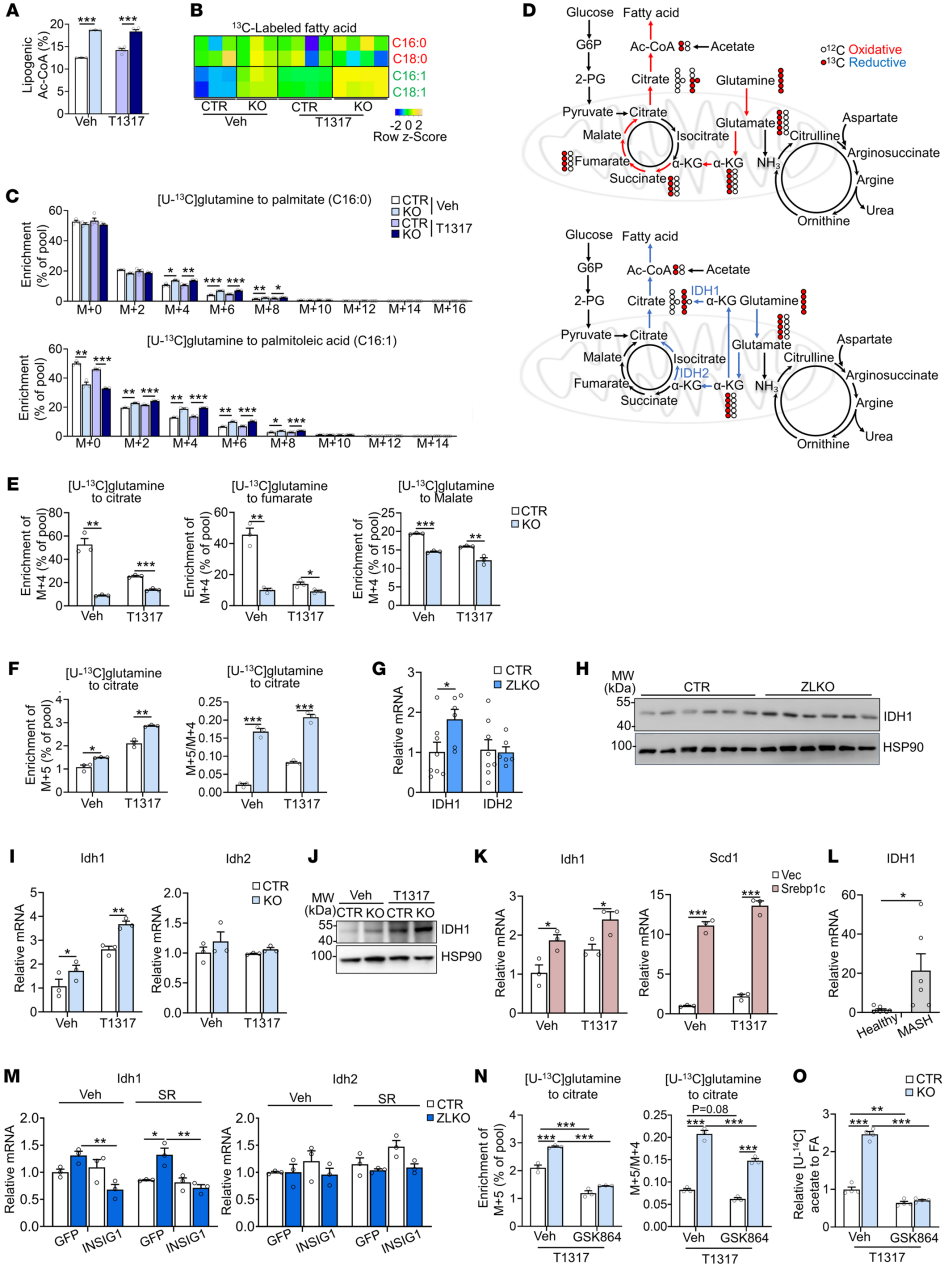
Minor intron splicing dysfunction activates reductive glutamine metabolism for de novo lipogenesis by inducing Idh1 expression. (**A**) Enrichment of lipogenic acetyl-CoA was calculated via FAMetA software in CTR and KO AML12 cells cultured with [U-^13^C]glutamine for 24 hours and then treated with Veh (*n* = 3) or 5 μM T1317 (*n* = 4) for 24 hours. (**B**) Heatmap representing the enrichment of ^13^C-labeled fatty acids in CTR and KO AML12 cells. (**C**) Mass isotopologue distribution analysis of palmitate and palmitoleic acid in CTR and KO AML12 cells (*n* = 3). (**D**) Schematic diagram showing the oxidative (red) and reductive (blue) metabolic flux of [U-^13^C]glutamine. (**E** and **F**) Mass isotopologue distribution analysis of citrate, fumarate, and malate (M+4) (**E**) and citrate (M+5) and the ratio of M+5– to M+4–labeled citrate (**F**) in CTR and KO AML12 cells (*n* = 3). (**G**) qPCR analysis of Idh1 and Idh2 expression in livers of CTR (*n* = 8) and ZLKO (*n* = 6) mice fed a normal chow diet. (**H**) Immunoblot analysis of the liver lysates from **G**. (**I**) qPCR analysis of Idh1 and Idh2 expression in CTR and KO AML12 cells (*n* = 3) treated with Veh or T1317 (5 μM) for 24 hours. (**J**) Immunoblot analysis of the cell lysates from **I**. (**K**) qPCR analysis of Idh1 and Scd1 expression in AML12 cells stably overexpressing vector (Vec, *n* = 3) or Srebp1c (*n* = 3) after Veh and T1317 treatment. (**L**) qPCR analysis of Idh1 expression in liver samples from healthy donors (*n* = 8) and patients with MASH (*n* = 6). (**M**) Primary hepatocytes (*n* = 3) were isolated from CTR and ZLKO mouse livers and infected with GFP and Insig1 adenoviruses. Expression of Idh1 and Idh2 was measured by qPCR after Veh or SR9238 (10 μM) treatment for 24 hours. (**N**) Mass isotopologue distribution analysis of citrate (M+5) and the ratio of M+5– to M+4–labeled citrate in CTR and KO AML12 cells (*n* = 3) cultured with [U-^13^C]glutamine and treated with T1317 (5 μM) in combination with Veh and GSK864 (10 μM) for 24 hours. (**O**) Incorporation of [U-^14^C]acetate into lipids. Data are presented as mean ± SEM. *P < 0.05, **P < 0.01, ***P < 0.001 by 2-tailed unpaired Student’s *t* test (**A**, **C**, **E**–**G**, **I**, **K**, and **L**) and by 2-way ANOVA with multiple comparisons (**M**–**O**).

Increased hepatic de novo lipogenesis may disrupt metabolic homeostasis in mice with minor intron splicing deficiency to promote MASH progression. Thus, we performed a metabolomics analysis of livers of control and ZLKO mice after T1317 treatment to evaluate whether metabolic remodeling upon minor intron splicing inactivation triggered MASH progression. De novo lipogenesis generally incorporates acetyl-CoA generated by glycolysis into fatty acids; however, our metabolomics results imply that the increased metabolites in *Zrsr1* and *Zrsr2* double-deficient livers were enriched in amino acid metabolism and the urea cycle (Figure 6A). The glycolysis and pentose phosphate pathways, which regularly contribute to de novo lipogenesis, were not significantly altered (Figure 6B). Intriguingly, levels of the TCA cycle intermediates citrate and α-ketoglutarate (α-KG) and ATP/ADP and NADH/NAD ratios increased, indicating that a lipogenic environment was formed in the livers of ZLKO mice (Figure 6, C and D). Based on [U-^14^C]–labeled acetate tracing experiments, we further confirmed that the disruption of minor intron splicing in hepatocytes increased the de novo synthesis of fatty acids from acetate-derived acetyl-CoA; however, [U-^14^C]–labeled glucose utilization for de novo lipogenesis decreased, especially upon LXR activation, suggesting that glycolysis was not the major carbon source for de novo lipogenesis induced by minor intron splicing deficiency (Figure 6, E and F). The increase in urea cycle metabolites suggested elevated amino acid degradation, which may have served as an alternative carbon source for de novo lipogenesis (Figure 6G). To verify this hypothesis, we used [U-^13^C]–labeled glutamine, one of the most abundant amino acids, as a tracer to assess the incorporation of amino acid–derived acetyl-CoA into de novo synthesized fatty acids. The calculated lipogenic acetyl-CoA contribution was increased in Zrsr1 and Zrsr2 double-inactivated hepatocytes, suggesting that amino acid metabolism–mediated lipogenesis was induced (Figure 7A). Interestingly, total ^13^C-labeled monounsaturated fatty acids (C16:1 and C18:1) but not saturated fatty acids (C16:0 and C18:0) were significantly increased in *Zrsr1* and *Zrsr2* double-deficient hepatocytes under both basal and LXR-activated conditions (Figure 7B). However, mass isotopomer distribution analysis revealed that M+4–, M+6–, and M+8–labeled palmitate (C16:0), representing palmitate containing 2, 3, and 4 acetyl-CoA molecules derived from [U-^13^C]glutamine, respectively, were increased by minor intron splicing disruption; M+2– to M+8–labeled palmitoleic acid (C16:1) was more profoundly increased along with a decrease in unlabeled (M+0) palmitoleic acid in *Zrsr1* and *Zrsr2* double-deficient hepatocytes, implying a high preference for glutamine-derived lipogenic carbon usage for monounsaturated fatty acid synthesis through de novo lipogenesis upon minor intron splicing deficiency. The activation of LXR specifically increased the formation of monounsaturated fatty acids from glutamine (Figure 7C). Amino acids can be metabolized into TCA cycle intermediates to oxidatively generate citrate, which is used for de novo lipogenesis. Under certain conditions, amino acids, especially glutamine, undergo reductive carboxylation to generate citrate (Figure 7D). To determine the metabolic route through which glutamine generates citrate for de novo lipogenesis in minor intron splicing–deficient hepatocytes, we performed a metabolic flux assay using a [U-^13^C]–labeled glutamine tracer to analyze the amount of oxidatively and reductively synthesized citrate. Enrichment of M+4–labeled citrate, fumarate, and malate, which represent the oxidative pathway of glutamine metabolism, was attenuated. LXR activation further inhibited this oxidative route of citrate synthesis from glutamine (Figure 7E). Then, we measured M+5–labeled citrate, which represents the citrate derived from glutamine through reductive carboxylation. The level of M+5–labeled citrate and the ratio of M+5– to M+4–labeled citrate increased in minor intron splicing–inactivated hepatocytes. In addition, T1317 treatment further elevated the reductive carboxylation of glutamine (Figure 7F).

IDH1 and IDH2 are key dehydrogenases located in the cytosol and mitochondria, respectively, that catalyze the reductive metabolism of α-KG to citrate. A previous report revealed that SREBP signaling regulates *Idh1* expression (42). Here, we also observed that *Idh1* expression was increased in the livers of ZLKO mice at both the mRNA and protein levels, while *Idh2* expression was not altered (Figure 7, G and H, and Supplemental Figure 7A). Induction of *Idh1* but not *Idh2* was also confirmed in AML12 cells with minor intron splicing defects. Expression of *Idh1* was further augmented under LXR-activated conditions (Figure 7, I and J). Furthermore, overexpression of *Srebp1c* in AML12 cells directly increased the expression of *Idh1* and the lipogenic gene *Scd1*, which was further elevated by T1317 treatment (Figure 7K). Upregulation of *Idh1* expression was also observed in the livers of patients with MASH (Figure 7L). Interestingly, *Insig1* overexpression by adenoviral transduction rescued the increase in *Idh1* expression in minor intron splicing–deficient primary hepatocytes, further confirming that dysfunctional minor intron splicing induced *Idh1* expression through SREBP1c activation (Figure 7M). Notably, suppressing IDH1 activity via the IDH1 inhibitor GSK864 attenuated basal and stimulated M+5–labeled citrate production and the M+5/M+4–labeled citrate ratio in hepatocytes deficient in minor intron splicing upon T1317 treatment (Figure 7N). As a result, de novo lipogenesis, as indicated by [U-^14^C]–labeled acetate flux, was substantially blocked (Figure 7O). Taken together, these results support that stimulation of SREBP1c activity in minor intron splicing–inactivated hepatocytes drove reductive carboxylation of glutamine to generate lipogenic carbon for de novo lipogenesis through induction of *Idh1* expression.

### IDH1-mediated glutamine reductive carboxylation flux induces hepatic fibrogenesis via ammonia-driven activation of hepatic stellate cells.

**Figure 8:**
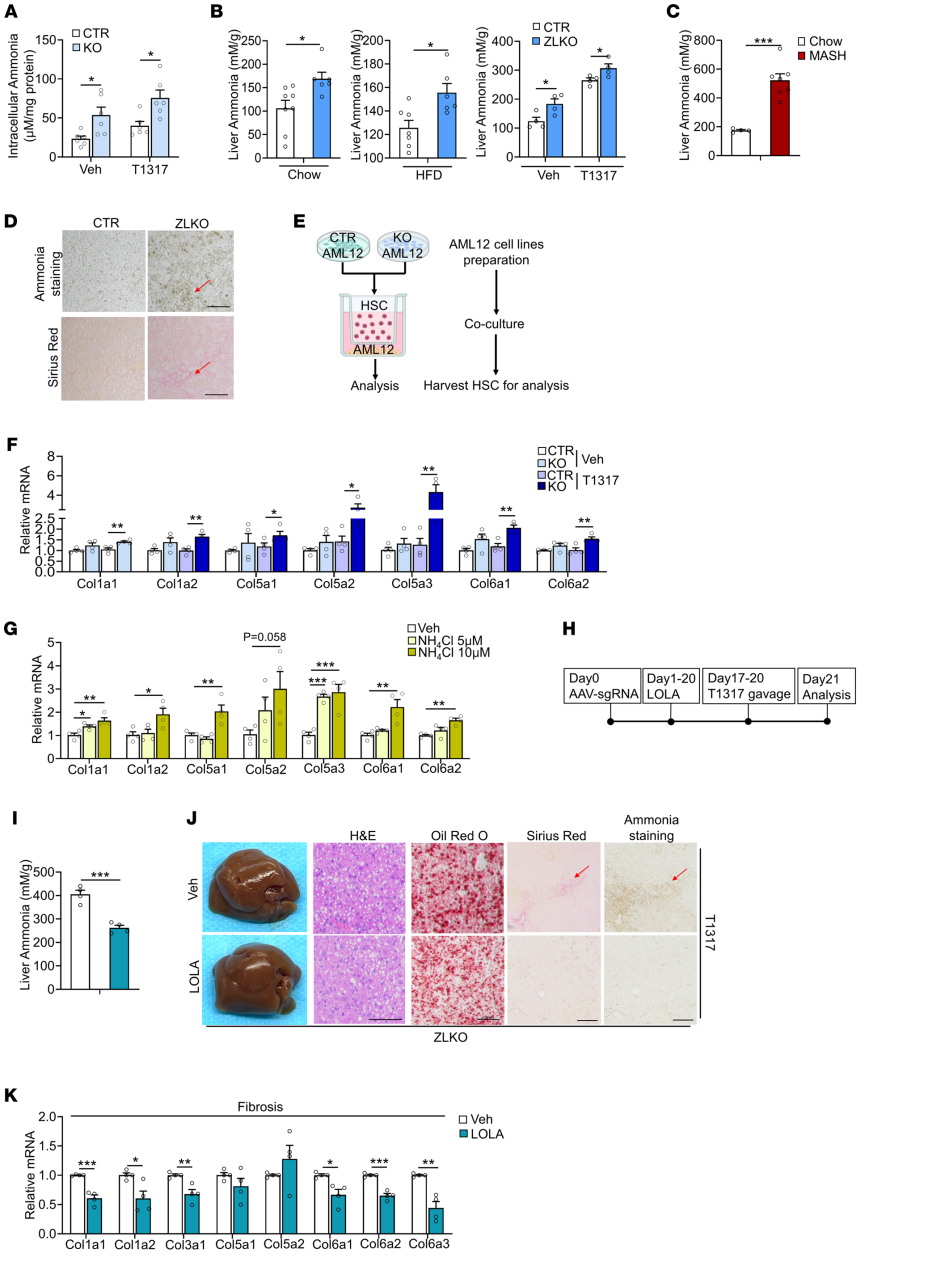
IDH1 induces ammonia accumulation in the liver, which triggers HSC activation to drive hepatic fibrogenesis. (**A**) Ammonia levels were measured in CTR and KO AML12 cells (*n* = 6) after vehicle (Veh) or T1317 (5 μM) treatment for 24 hours. (**B**) Ammonia levels were measured in livers of CTR and ZLKO mice after being fed chow diet (left panel: CTR, *n* = 8, ZLKO, *n* = 6), HFD (middle: CTR, *n* = 7, ZLKO, *n* = 6), or oral gavage of vehicle or T1317 (25 mg/kg/d) for 4 days (right: CTR, *n* = 4, ZLKO, *n* = 4). (**C**) Ammonia levels were measured in livers of mice fed a chow diet (*n* = 4) or CDA-HFD (MASH diet, *n* = 7) for 6 months. (**D**) Ammonia staining and Sirius red staining of livers from CTR and ZLKO mice treated with T1317 (scale bars: 100 μm). (**E**) Schematic diagram showing the strategy of coculture of CTR and KO AML12 cells with HSCs. (**F**) qPCR analysis of genes involved in fibrosis in cocultured HSCs (*n* = 4). (**G**) qPCR analysis of fibrotic genes in HSCs (*n* = 4) treated with Veh or 5 or 10 μM NH_4_Cl for 24 hours. (**H**) Diagram of the study design. (**I**) Ammonia levels were measured in livers of ZLKO mice intraperitoneally injected daily with vehicle (Veh, *n* = 4) or 2 g/kg/d LOLA (*n* = 4) for 17 days and then combined with oral administration of T1317 (25 mg/kg/d) for 4 days. (**J**) General morphology, H&E staining, Oil Red O staining, Sirius red staining, and ammonia staining (scale bars: 100 μm). (**K**) qPCR analysis of hepatic genes involved in fibrosis. The red arrows in **D** and **J** indicate the area where collagen and ammonia accumulated. Data are presented as mean ± SEM. *P < 0.05, **P < 0.01, ***P < 0.001 by 2-tailed unpaired Student’s *t* test (**A**–**C**, **F**, **I**, and **K**) and by 1-way ANOVA with Dunnett’s test (**G**).

**Figure 9:**
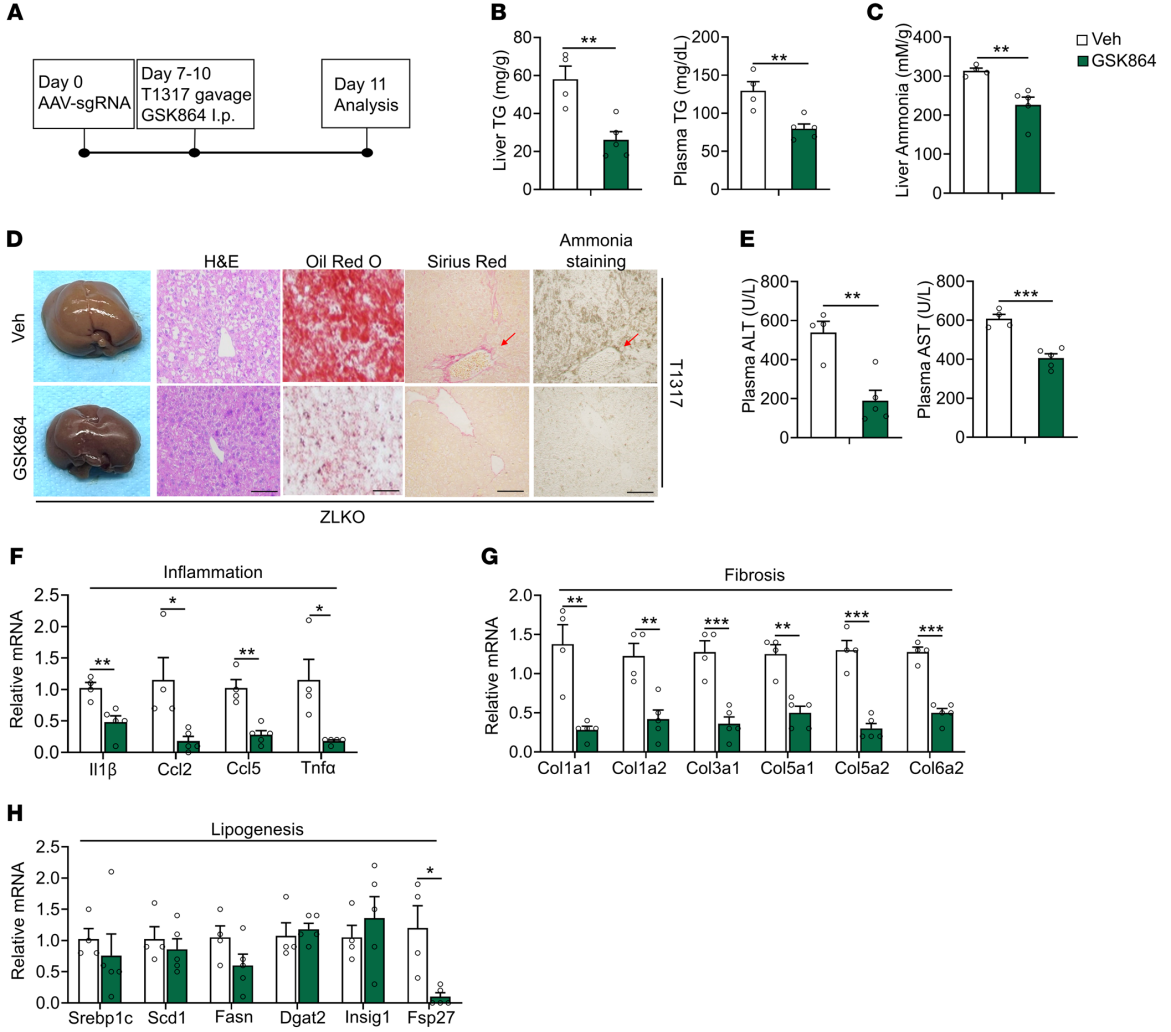
IDH1 inhibition blocks minor intron splicing deficiency–induced MASH progression. (**A**) Diagram of the study design. (**B** and **C**) Liver and plasma TG content (**B**) and hepatic ammonia content (**C**) in ZLKO mice intraperitoneally injected with Veh (*n* = 4) or GSK864 (75 mg/kg/d, *n* = 5) combined with oral administration of T1317 (25 mg/kg/d) for 4 days. (**D**) General morphology, H&E staining, Oil Red O staining, Sirius red staining, and ammonia staining (scale bars: 100 μm). (**E**) Plasma ALT and AST levels. (**F**–**H**) qPCR analysis of hepatic genes involved in inflammation (**F**), fibrosis (**G**), and lipogenesis (**H**). The red arrows in **D** indicate the area where collagen and ammonia accumulated. Data are presented as mean ± SEM. *P < 0.05, **P < 0.01, ***P < 0.001 by 2-tailed unpaired Student’s *t* test (**B**, **C**, and **E**–**H**).

MASH progression is accompanied by increased fibrosis. However, how enhanced de novo lipogenesis triggers hepatic fibrogenesis is still controversial. The reductive carboxylation–mediated lipogenesis that occurs upon minor intron splicing deficiency may reshape the local liver microenvironment to induce hepatic stellate cell activation. Glutamine contains 2 amino groups that efficiently transport ammonia in circulation to the liver for clearance by the urea cycle. The conversion of glutamine to α-KG for reductive carboxylation may release a significant amount of ammonia and increase the urea cycle flux. It is possible that overwhelming ammonia accumulation in the liver is beyond the ZLKO mouse liver detoxification capacity, thus leading to MASH progression. Here, we found that the ammonia concentration was increased in minor intron splicing–deficient hepatocytes and further augmented upon LXR activation (Figure 8A). Furthermore, mice with a loss of minor intron splicing activity presented significantly elevated ammonia content in the liver under normal, obese, and LXR-activated conditions (Figure 8B). Notably, hepatic ammonia content drastically increased in mice in the MASH stage (Figure 8C). Staining of ammonia by Nessler reagent supported the increase in ammonia deposition in the livers of ZLKO mice upon LXR activation, and more importantly, the ammonia-stained area was strongly associated with collagen deposition, as indicated by Sirius red staining (Figure 8D). Hepatic stellate cells (HSCs) largely contribute to collagen production in the liver. Therefore, we performed a coculture experiment using Transwells, which allowed ammonia secreted from minor intron splicing–defective hepatocytes to influence cocultured HSCs (Figure 8E). The results revealed that the secreted product of *Zrsr1* and *Zrsr2* double-inactivated hepatocytes induced HSC collagen gene expression upon T1317 treatment (Figure 8F). Furthermore, ammonium chloride treatment dose-dependently induced collagen gene expression in HSCs (Figure 8G). L-ornithine-l-aspartate (LOLA) is a reagent capable of lowering hepatic ammonia by enhancing the urea cycle (43). We pretreated ZLKO mice with LOLA or the control for 17 days and subsequently combined with the activation of LXR via T1317 treatment (Figure 8H). LOLA treatment specifically attenuated hepatic ammonia content without affecting body weight, liver weight, liver or plasma triglyceride levels, or cholesterol content in the ZLKO mice (Figure 8I and Supplemental Figure 7, B–E). As a consequence, hepatic fibrosis was reduced, whereas liver steatosis, inflammation, and injury were not affected (Figure 8J and Supplemental Figure 7F). Expression of collagen genes decreased upon LOLA treatment in ZLKO mice, suggesting a decrease in hepatic HSC activation, while lipogenesis- and inflammation-associated gene expression was not influenced (Figure 8K and Supplemental Figure 7, G and H). Idh1 has been shown to be the critical enzyme that regulates reductive carboxylation flux and ammonia production in the livers of minor intron splicing–deficient mice. Thus, we further investigated whether inhibition of IDH1 is sufficient to block minor intron splicing deficiency–induced MASH progression. We intraperitoneally injected the IDH1 inhibitor GSK864 or the control into ZLKO mice and treated them with T1317 (Figure 9A). Body weight and liver weight of the GSK864-treated ZLKO mice substantially decreased, and liver triglyceride, plasma triglyceride, and cholesterol content was also drastically reduced (Figure 9B and Supplemental Figure 7, I–K), suggesting that reductive carboxylation*-*mediated de novo lipogenesis in the liver was suppressed. More importantly, hepatic ammonia levels in ZLKO mice were reduced after GSK864 treatment, probably due to the reversal of glutamate deamination (Figure 9C). As a result, hepatic steatosis, fibrosis, and ammonia accumulation were largely diminished in the livers of IDH1 activity–inhibited ZLKO mice (Figure 9D). Therefore, liver injury, as indicated by ALT and AST levels, was attenuated (Figure 9E). Notably, expression of hepatic inflammation- and fibrosis-associated genes was profoundly suppressed, supporting the amelioration of minor intron splicing deficiency–induced MASH progression after treatment with GSK864 (Figure 9, F and G). Expression of lipogenic markers and SREBP1c processing were not altered; however, the lipid marker *Fsp27* was significantly reduced (Figure 9H and Supplemental Figure 7L). These results led to the conclusion that the increase in IDH1 activity upon minor intron splicing deficiency enhanced hepatic ammonia production by inducing reductive carboxylation flux of glutamine, which initiated HSC activation and hepatic fibrogenesis. Inhibition of IDH1 activity efficiently reduced the minor intron splicing inactivation–induced MASH phenotype upon LXR activation.

### Restoration of minor intron splicing activity or targeting of the IDH1-ammonia axis in the liver ameliorates MASH progression.

**Figure 10:**
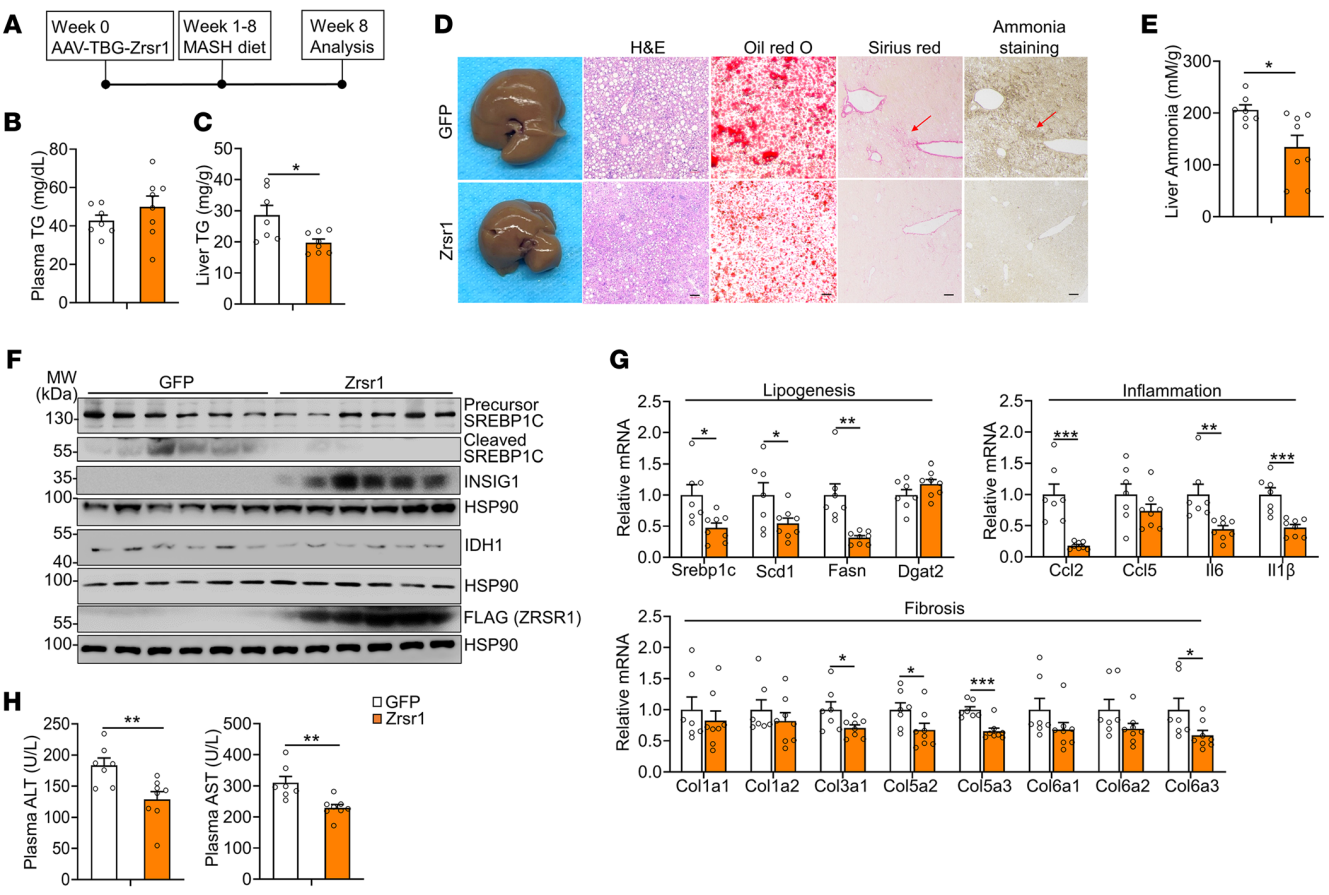
Reactivating minor intron splicing ameliorates MASH progression. (**A**) Diagram of the study design. (**B** and **C**) Plasma (**B**) and liver (**C**) TG content of mice injected with AAV-TBG-GFP (GFP, *n* = 7) or AAV-TBG-Zrsr1 (Zrsr1, *n* = 8) after CDA-HFD (MASH diet) feeding for 8 weeks. (**D**) General morphology, H&E staining, Oil Red O staining, Sirius red staining, and ammonia staining (scale bars: 100 μm). (**E** and **F**) Liver ammonia levels (**E**) and immunoblotting of liver lysates (**F**). (**G**) qPCR analysis of hepatic genes involved in lipogenesis, inflammation, and fibrosis. (**H**) Plasma ALT and AST levels. The red arrows in **D** indicate the area where collagen and ammonia accumulated. Data are presented as mean ± SEM. *P < 0.05, **P < 0.01, ***P < 0.001 by 2-tailed unpaired Student’s *t* test (**B**, **C**, **E**, **G**, and **H**).

Our previous results demonstrated that downregulation of Zrsr1 and Zrsr2 in the liver leads to minor intron retention, which triggers activation of the SREBP1c/IDH1/ammonia axis to induce MASH progression. To explore whether intervention with minor intron splicing factors or the IDH1/ammonia axis blocks MASH progression, we overexpressed Zrsr1 in hepatocytes of mice via AAV under the liver-specific thyroxine-binding globulin (TBG) promoter and subjected the mice to MASH diet feeding (Figure 10A). Hepatic overexpression of Zrsr1 reversed the minor intron retention of Insig1 and Insig2 in the livers of mice with MASH (Supplemental Figure 8, A and B). Although Zrsr1 overexpression in the livers of the mice did not affect body weight, the glucose level was slightly reduced (Supplemental Figure 8, C and D). Notably, liver weight gain and hepatic triglyceride accumulation were reversed in hepatic Zrsr1-overexpressing mice, but hepatic cholesterol levels remained unchanged, but hepatic cholesterol, plasma triglyceride, and plasma cholesterol levels were unaltered (Figure 10, B and C, and Supplemental Figure 8, E and F). After MASH diet feeding, the livers of the Zrsr1-overexpressing mice appeared to be smaller and smoother in morphology. Liver histology, Oil Red O staining, and Sirius red staining suggested that the overexpression of *Zrsr1* in the liver impeded MASH progression, as indicated by the blockade of steatosis, inflammation, fibrosis, and ammonia accumulation (Figure 10, D and E). The cleavage of the SREBP1c precursor, reduction in *Insig1* expression, and increase in *Idh1* expression due to minor intron splicing inactivation in MASH were significantly reversed by *Zrsr1* overexpression (Figure 10F and Supplemental Figure 8G). Consistent with these findings, expression of genes associated with lipogenesis, inflammation, and fibrosis was substantially suppressed, indicating that the MASH phenotypes were extensively relieved (Figure 10G). Additionally, liver damage, as indicated by ALT and AST levels, was reduced (Figure 10H). These results suggest that overexpression of *Zrsr1*, which reactivated minor intron splicing activity, prevented the progression of MASH.

**Figure 11:**
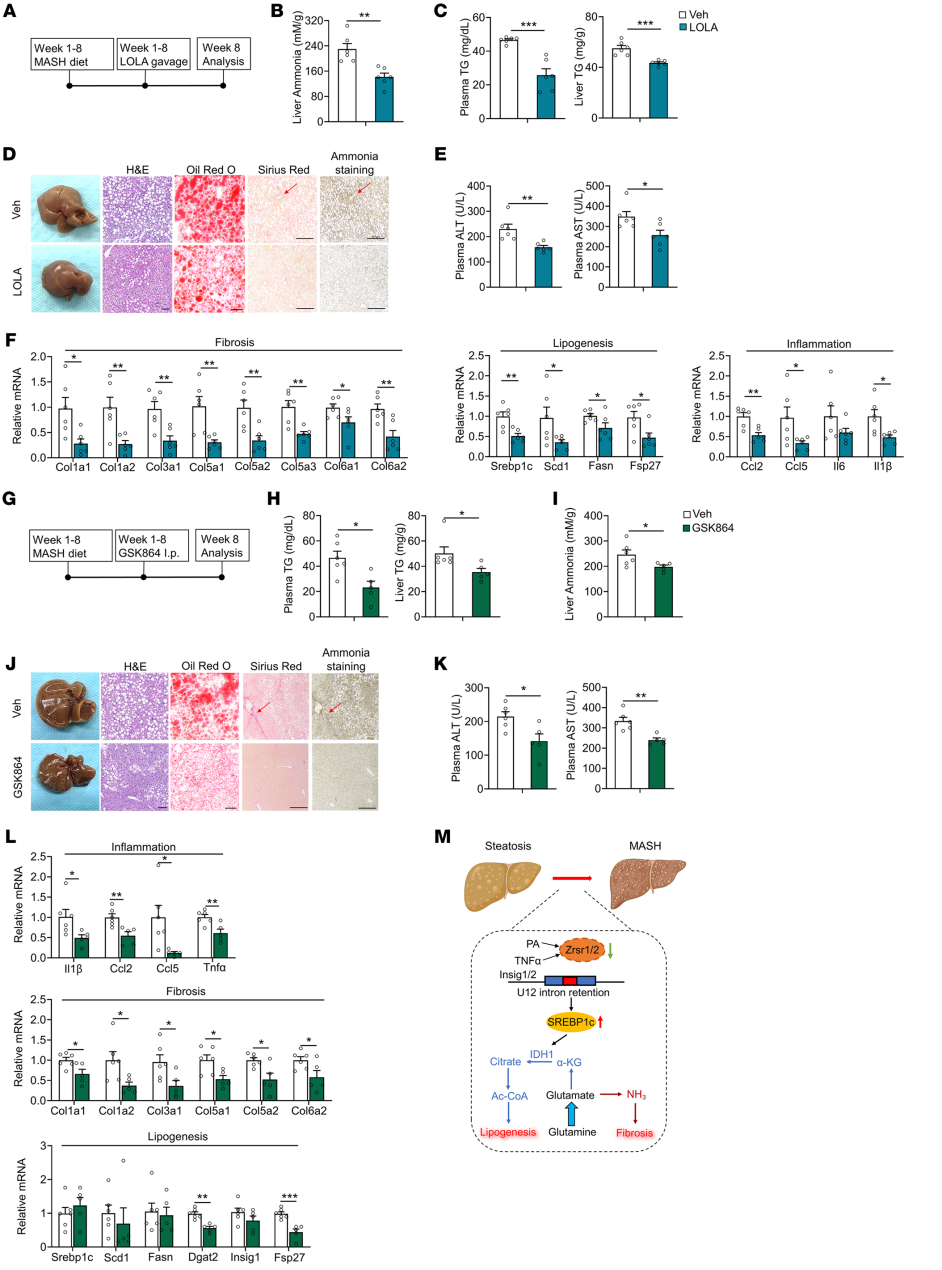
Drug administration targeting the IDH1/ammonia axis ameliorates MASH progression. (**A**) Diagram of the study design. (**B**) Hepatic ammonia levels in WT mice administrated saline (*n* = 6) or 2 g/kg/d LOLA (*n* = 6) daily through oral gavage, with both groups fed a MASH diet for 8 weeks. (**C**) Plasma (left) and liver (right) TG content. (**D**) General morphology, H&E staining, Oil Red O staining, Sirius red staining, and ammonia staining (scale bars: 100 μm). (**E**) Plasma ALT and AST levels. (**F**) qPCR analysis of hepatic genes involved in fibrosis (left panel), lipogenesis (middle), and inflammation (right). (**G**) Diagram of the study design. (**H**) Plasma (left panel) and liver (right) TG content in WT mice intraperitoneally injected daily with vehicle (Veh, *n* = 6) or 75 mg/kg/d GSK864 (*n* = 5), with both groups fed a MASH diet for 8 weeks. (**I**) Hepatic ammonia content. (**J**) General morphology, H&E staining, Oil Red O staining, Sirius red staining, and ammonia staining (scale bars: 100 μm). (**K**) Plasma ALT and AST levels. (**L**) qPCR analysis of hepatic genes involved in inflammation (top panel), fibrosis (middle), and lipogenesis (bottom). (**M**) Schematic illustration of the mechanism of the MASH progression by which disruption of minor intron splicing caused by the reduction of Zrsr1 and Zrsr2 triggers SREBP1c-dependent IDH1-mediated glutamine reductive carboxylation flux for de novo lipogenesis, thus activating HSCs via excessive ammonia production. The red arrows in **D** and **J** indicate the area where collagen and ammonia accumulated. Data are presented as mean ± SEM. *P < 0.05, **P < 0.01, ***P < 0.001 by 2-tailed unpaired Student’s *t* test (**B**, **C**, **E**, **F**, **H**, **I**, **K**, and **L**).

Next, we aimed to investigate the therapeutic potential of targeting the IDH1/ammonia axis. Initially, we treated the mice with LOLA to suppress ammonia accumulation during MASH diet feeding (Figure 11A). Upon being harvested, body weights of the LOLA-treated mice significantly decreased, whereas there was no difference in liver weight (Supplemental Figure 8H). The hepatic ammonia content was largely reduced in the mice after LOLA treatment, along with extensively decreased liver triglyceride and cholesterol content, while the plasma triglyceride level was also suppressed. The plasma cholesterol level was unchanged (Figure 11, B and C, and Supplemental Figure 8I). Liver histology presented improved hepatic steatosis, collagen deposition, and ammonia accumulation (Figure 11D) in the mice treated with LOLA, while liver injury, as indicated by ALT and AST levels, was reduced (Figure 11E). Additionally, hepatic fibrosis-, lipogenesis-, and inflammation-related gene expression was suppressed (Figure 11F). These results indicated that reducing hepatic ammonia levels effectively ameliorated MASH progression. Then, we further investigated the potential therapeutic effect of targeting IDH1. We administered the IDH1 inhibitor GSK864 in a MASH mouse model (Figure 11G). In mice fed a MASH diet, GSK864 treatment significantly decreased body weight and suppressed triglyceride levels in both liver and plasma and ammonia content in liver, but did not affect liver weight or change plasma or hepatic cholesterol levels (Figure 11, H and I,and Supplemental Figure 8, J and K). Notably, the MASH phenotypes — including lipid and ammonia accumulation, collagen deposition in the liver, and liver injury — were substantially relieved upon GSK864 treatment (Figure 11, J and K). Moreover, hepatic inflammation- and fibrosis-related gene expression was extensively reduced. Consistent with previous results, GSK864 treatment significantly decreased expression of the lipid marker *Fsp27* but did not alter lipogenic gene expression (Figure 11L). Taken together, these results strongly support that targeting the IDH1/ammonia axis is a promising strategy for MASH therapy.

## Discussion

Hepatic de novo lipogenesis is a fundamental metabolic process that is greatly elevated during MASLD progression. However, little is known regarding the preference for carbon sources in the liver during activation of de novo lipogenesis in the MASH stage and its role in facilitating MASH progression. In this study, we revealed that hepatic minor intron splicing activity was substantially attenuated during MASH development and triggered SREBP1c-mediated de novo lipogenesis. Interestingly, in hepatic minor intron splicing–inactivated mice, amino acids, especially glutamine, were used as lipogenic carbons. Intriguingly, glutamine was reductively carboxylated into citrate to support de novo lipogenesis. This metabolic rewiring may be a critical checkpoint for the MASH transition (Figure 11M). Several lines of evidence support this notion. First, expression of the minor intron splicing factors *Zrsr1* and *Zrsr2* in the liver was drastically attenuated in mice with MASH and patients with MASH. In addition, it was synergistically downregulated by fatty acids and inflammatory cytokines, which are important pathogenic factors of MASH. A decrease in *Zrsr1* and *Zrsr2* resulted in compromised minor intron splicing activity. Second, mice with hepatic *Zrsr1* and *Zrsr2* depletion exhibited spontaneous hepatic steatosis and fibrosis phenotypes that were independent of dietary conditions. These two MASH signatures were further exacerbated upon HFD-induced insulin resistance and LXR activation. Notably, the minor introns of both *Insig1* and *Insig2* were retained; this resulted in the formation of prematurely terminated INSIG1 and INSIG2, thereby decreasing the anchoring of SREBP1c to the ER and inducing more extensive SREBP1c proteolytic activation. Interestingly, the decrease in WT *Insig1* transcript due to minor intron retention was more profound than that of *Insig2*, indicating that the dysfunction of Insig1 may be more important than Insig2 for proteolytic activation of SREBP1c under MASH conditions. Importantly, we demonstrated that elevated SREBP1c activity was sufficient to drive the MASH phenotype under LXR activation, although SREBP2-mediated cholesterol metabolism was not substantially affected. Third, inactive minor intron splicing upon LXR activation led to an increase in amino acid degradation instead of glycolysis. Additionally, metabolic flux analyses revealed an increase in glutamine usage for lipid synthesis, suggesting that glutamine substantially replaced glucose as a dominant carbon source at the MASH stage. A recent study demonstrated that amino acids from dietary protein are a major carbon supplier for hepatic de novo lipogenesis, especially during MASLD progression (44). This discovery supports our findings, and we further revealed that minor intron retention was the key driver that led to a significant shift in carbon source utilization, from glucose to glutamine, under MASH condition. This point was further supported by the increase in glutaminolysis in patients with MASH reported previously (45). Interestingly, we revealed that glutamine-derived lipogenic carbon was largely incorporated into monounsaturated fatty acids, suggesting that glutaminolysis at the MASH stage was specific for monounsaturated fatty acid synthesis. Unsaturation of fatty acids was enhanced in MASH, as previously reported (46). An increase in the amount of monounsaturated fatty acids may promote MASH/HCC progression by inhibiting ferroptosis (47). A previous report showed that ammonia accumulation induces de novo lipogenesis by activating SREBP1c in the liver (48). Here, we demonstrated that highly activated de novo lipogenesis caused by disrupted minor intron splicing generated ammonia accompanied by glutamine reductive carboxylation flux to facilitate stellate cell activation and exacerbate the fibrosis phenotype, which may have formed a positive feedback loop for MASH progression. However, the detailed mechanisms underlying the changes caused by minor intron retention in the preference for glutamine as a major carbon source and the exclusive generation of monounsaturated fatty acids in livers deficient in minor intron splicing need further investigation. Interestingly, increased SREBP1c activity in minor intron splicing–deficient hepatocytes induces *Idh1* expression, which catalyzes the reductive carboxylation flux of cytosolic glutamine under certain conditions, such as hypoxia (25). This unique metabolic pathway is also frequently observed in cancer cells. In this study, we revealed that this reductive carboxylation route was activated by disrupted minor intron splicing during MASH progression. Enhanced *Idh1* levels were observed in the livers of ZLKO mice, and these changes were reversed by *Zrsr1* overexpression. Inhibition of IDH1 activity substantially blocked induction of de novo lipogenesis in hepatocytes and hepatic steatosis, inflammation, and especially fibrosis in mice with defective minor intron splicing. Finally, overexpression of *Zrsr1* restored minor intron splicing activity and reversed aberrant de novo lipogenesis in MASH, thereby blocking MASH progression. More importantly, targeting the IDH1/ammonia axis via clearance of ammonia accumulated in the liver and inhibition of IDH1 activity profoundly suppressed MASH progression, thus providing convincing evidence that IDH1 activation–induced metabolic reprogramming could be a promising target for MASH therapy.

Taken together, our results suggest that disruption of minor intron splicing is a highly pivotal pathogenic factor for MASH progression. The effect of minor intron splicing inactivation may synergize with obesity-induced insulin resistance and LXR activation to trigger MASH pathogenesis. Notably, we revealed that minor intron splicing defects were induced by MASH pathogenic factors, which indicates that inactivated minor intron splicing may be a critical checkpoint for irreversible liver damage in the MASH stage. Prevention of minor intron splicing inactivation and blockade of IDH1/ammonia-driven metabolic reprogramming during MASH progression may serve as a promising strategy for MASH therapy.

## Methods

Further information is provided in Supplemental Methods.

### Sex as a biological variable.

Our study examined male mice because male animals exhibited less variability in phenotype. However, the findings are expected to be relevant for both male and female animals.

### Animal studies.

C57BL/6J mice were maintained under 12-hour light/12-hour dark cycles and fed regular rodent chow, HFD (D12492, Research Diets), low-fat diet (LFD) (D12450J, Research Diets), or CDA-HFD (MASH diet) (A06071309, Research Diets). WT C57BL/6J mice were purchased from Shanghai Lingchang Biotechnology, and *Cas9*-knock-in mice were purchased from the The Jackson Laboratory (JAX stock 024858). For the generation of hepatic *Zrsr1* and *Zrsr2* double-deficient mice, we designed 2 sgRNAs flanking the *Zrsr1* gene, which contains a single exon, and the *Zrsr2* gene within exon 1 and exon 2 via a CRISPR design web tool (49). Each sgRNA targeting *Zrsr1* and *Zrsr2* was cloned downstream of the U6 promoter, and 2 tandem U6-sgRNA cassettes were constructed into the AAV vector. *Zrsr1* and *Zrsr2* double-deficient mice were generated by transducing *Cas9*-transgenic mice with a recombinant AAV vector expressing 2 sgRNAs targeting *Zrsr1* and *Zrsr2*. For AAV transduction, we injected approximately 1 × 10^11^ genome copies of AAV vectors per mouse via the tail vein. The AAV8 serotype was used for relative liver enrichment (50). For the oral gavage experiment, 2-month-old mice were orally administered a dose of 25 mg/kg T1317 (Selleck, S7076) or 20 mg/kg SR9238 (MCE, HY-101442) dissolved in sunflower oil or sunflower oil alone for 4 days. For the ammonia clearance experiment, the mice were intraperitoneally injected with 2 g/kg LOLA (MilliporeSigma, O7125) or saline every day for 17 days and then orally administered 25 mg/kg/d T1317 for 4 days. For GSK864 treatment, 75 mg/kg GSK864 (Selleck, S7994) was intraperitoneally injected into the mice before oral administration of 25 mg/kg T1317. For the LOLA and GSK864 treatment in a MASH model, 2 g/kg LOLA was administered through oral gavage and 75 mg/kg GSK864 was injected intraperitoneally on a daily basis during MASH diet feeding. More detailed information is provided in Supplemental Methods.

### Statistics.

All statistical analyses were performed via GraphPad Prism 9. Statistical differences were evaluated via 2-tailed unpaired Student’s *t* tests for comparisons between 2 groups or ANOVA and appropriate post hoc analyses for comparisons of more than 2 groups. A *P* value of less than 0.05 was considered to indicate statistical significance. The statistical methods and corresponding *P* values for the data shown in each panel are included in the figure legends.

### Study approval.

All animal studies were performed according to procedures approved by the University Committee on the Use and Care of Animals at Shanghai Jiao Tong University. Animal care was performed in accordance with institutional guidelines. Human liver biopsy samples from patients with MASH and healthy liver transplant donors were obtained from Shanghai Renji Hospital. All research was conducted in accordance with both the Declaration of Helsinki and the Declaration of Istanbul. All research was approved by the Institutional Review Board of Renji Hospital affiliated to Shanghai Jiao Tong University School of Medicine (IRB reference KY2020-190), and written consent was given by all the participants after the nature and possible consequences of the studies were explained.

### Data availability.

The RNA-Seq data files have been deposited in the Gene Expression Omnibus database (GEO GSE252030 and GSE272322). Values for all data points in graphs are reported in the Supporting Data Values file. The data generated in this study are available upon request.

## Author contributions

YF, XP, HS, XL, YZ, JT, YL, DC, WL, JZ, and XYZ conceived the project and designed the research. YF performed the majority of the studies. XP, HS, YL, DC, and WL performed some animal and cell experiments. YF, JM, MH, YM, and XYZ analyzed the data and wrote the manuscript.

## Supplementary Material

Supplemental tables and

Supplemental data

Unedited blot and gel images

Supporting data values

## Version 1 03/18/2025

In-Press Preview

## Version 2 05/15/2025

Electronic publication
