# Supplementary material for: Disrupted minor intron splicing activates reductive carboxylation-mediated lipogenesis to drive metabolic dysfunction–associated steatotic liver disease progression
Source: J Clin Invest. 2025 Mar 18;135(10):e186478. doi: 10.1172/JCI186478 (PMC12077890; doi:10.1172/JCI186478)
Supplement: Supplemental tables and [file jci-135-186478-s230.pdf]

Supplemental information

Materials and Methods

Genotyping

Whole genomic DNA was derived from the tail or cell after lysing with 50 mM NaOH at 98°C for 30 min and neutralized with 1M Tris-HCl (pH 8.0). This crude DNA was then used for PCR using 2X Rapid Taq Master Mix (Vazyme, P222-01), according to the manufacturer's instructions and following PCR conditions: one cycle of 95°C for 3 min, 35 cycles of 95°C for 15 s, 60°C for 15 s, 72°C for 60 s, and one cycle of 72°C for 5 min. Primers for Zrsr1 KO; forward, cacggcagaccgcgattcctg; reverse, ggaagttgcagtgctttcctt; For Zrsr2 KO; forward, gagtgggcgaccgaggacct; reverse, ggagcaattccacatccagagc. The PCR products were subsequently separated using 1 % agarose gel electrophoresis for 30 min.

Cell lines

Primary hepatocytes were isolated from C57BL/6J mice via collagenase type II as previously described (1). Hepatocytes were maintained in DMEM containing 10% FBS and 1% penicillin–streptomycin at 37°C and 5% CO_2. Adenovirus infection was performed on the same day as isolation. After 24 h, the cells were treated with vehicle (DMSO), T0901317 (5 μM) or SR9238 (10 μM) (Sigma, SM1510) for 24 h. AML12 cells were purchased from the American Type Culture Collection (ATCC) and cultured in DMEM/F-12 (1:1) supplemented with 10% FBS, 1% penicillin, streptomycin, dexamethasone and ITS. HEK293T cells were

cultured in DMEM supplemented with 10% BCS and 1% penicillin–streptomycin. Immortalized murine hepatic stellate cells (HSCs) were obtained from Dr. Xuelian Xiong at Zhongshan Hospital, Fudan University in Shanghai, and cultured in DMEM supplemented with 10% FBS and 1% penicillin–streptomycin. The cell culture experiments were performed in triplicate and repeated at least three times.

Liver histology

Tissues were dissected and fixed in 10% formalin overnight at 4°C and subjected to paraffin embedding and H&E staining. Liver slices were fixed in O.C.T. (G6059, Servicebio) and then stained with Oil Red O. Sirius red staining was performed as previously described (2). Paraffin sections were subjected to ammonia staining with Nessler´s reagent (Sigma, 1090280100) as previously described (3).

Metabolic analyses

For the glucose tolerance test (GTT), the mice were fasted overnight (16 h) and injected intraperitoneally (IP) with a glucose solution at a dose of 1.0 g/kg body weight. For the insulin tolerance test (ITT), the mice were fasted for 4 h and IP injected with insulin at a dose of 1 U/kg body weight. Blood glucose concentrations were measured before and at 20, 45, 90 and 120 min after glucose or insulin injection. Liver triglycerides and cholesterol were extracted and measured as previously described (4). Plasma insulin concentrations were

measured via an ELISA kit (CrystalChem, 90096). Plasma triglycerides, NEFAs, β-hydroxybutyrate, alanine transaminase (ALT), aspartate transaminase (AST) and cholesterol were measured via commercial kits. The hydroxyproline level in the liver was measured via metabolomics. Liver and cellular ammonia contents were measured via an ammonia assay kit (Sigma, MAK310). Liver α-ketoglutarate (α-KG) was measured via an α-ketoglutarate assay kit (Cayman, 701350). Widely targeted metabolomics of liver tissues was performed by MetWare Biotechnology. Metabolites of liver tissues were detected based on the AB Sciex QTRAP 6500 LC–MS/MS platform. Enrichment analysis was performed via Metaboanalyst (www.metaboanalyst.ca).

Gene expression analyses

Mouse and human livers were extracted and immediately frozen in liquid nitrogen. Total RNA was extracted from tissues or hepatocytes via the TRIzol method following the manufacturer’s instructions. For RT–qPCR, 2 μg of RNA was reverse transcribed via HiScript II Q RT SuperMix (Vazyme, R222-01), followed by qPCR via SYBR Green. Relative mRNA expression was normalized to the expression of AP0 or 18S. The primers used for gene expression are listed in Table S3.

Immunoblotting analysis

Mouse liver tissue was extracted and immediately frozen in liquid nitrogen. Tissue lysates were prepared by homogenization in buffer containing 50 mM Tris

(pH=7.6), 130 mM NaCl, 5 mM NaF, 25 mM β-glycerophosphoate, 1 mM sodium orthovanadate, 10% glycerol, 1% Triton X-100, 1 mM DTT, 1 mM PMSF and the protease inhibitor cocktail. Total cell lysates were prepared in lysis buffer containing 50 mM Tris-HCl (pH=7.8), 137 mM NaCl, 10 mM NaF, 1 mM EDTA, 1% Triton X-100, 10% glycerol, and a protease inhibitor cocktail (Bimake, B14002) after three freeze–thaw cycles. The ZRSR1 customized antibody was developed by PTM BIO. Briefly, the antibody was produced from rabbits immunized with a HIS-tagged protein corresponding to the C-terminal region (amino acids 200-420) of the murine ZRSR1 protein. The antibody serum was subsequently purified using Protein A and antigen affinity chromatography. The other commercial antibodies used were ZRSR2 (PA5-41797) from Invitrogen, SREBP-1 (sc-13551) and INSIG1 (sc-390504) from Santa Cruz Biotechnology. HSP90 (13171-1-AP), FLAG (20543-1-AP), IDH1 (12322-1-AP), NF-κB p65 (65107451-1-AP), and JNK (66210-1-Ig) were obtained from Proteintech. Phospho-AMPK (2535S), AMPK (2532), phospho-NF-κB P65 (3033), phospho-JNK (4668) and cleaved caspase3 (9661) were obtained from Cell Signaling Technology.

RNA-Seq Data Analysis

Total RNA was denatured and enriched by oligo (dT)-attached magnetic beads to obtain mRNA. mRNAs were fragmented for cDNA synthesis. cDNAs were then subjected to End Repair, Add A and Adaptor Ligation, and PCR amplification. The RNA library was qualified and then sequenced on DNBseq-T7 using High-

throughput Sequencing Kit (PE 150). The fastq files were subjected to quality control via FastQC followed by alignment against the mouse reference genome (mm38) via the aligner HISAT2 (5). HTSeq program was employed to count the number of reads mapped to the reference obtained from NCBI for coding genes and from the IAOD website for major/minor intron regions (6, 7). The fold change of the counts of major/minor intron reads was calculated to evaluate the level of minor and major intron retention in MASH and ZLKO mice. The percentage of major/minor retained introns is calculated by dividing the number of major/minor retained introns by all major/minor introns in major/minor intron-containing genes. In total, 657 minor introns found within 606 genes, and 211,005 major introns found within 32,396 genes, were taken into consideration for the calculation. DESeq2 was used for differential expression analysis (8). Transcripts exhibiting significant differences of more than 1.4-fold in expression in the ZLKO group compared with the CTR group were selected for further analysis. Gene enrichment analysis was performed via DAVID (https://david.ncifcrf.gov/). The StringTie method was applied to reconstitute the Insig1 and Insig2 transcripts (9).

In vitro lipogenesis assay

AML12 cells were incubated in Krebs Ringer buffer supplemented with 0.1% fatty acid-free BSA. Each well was incubated with 0.2 μCi/mL [U-^{14}C]acetate (PerkinElmer, NEC085H001MC) or [U-^{14}C]glucose (PerkinElmer, NEC042X050UC) for 2 h in the presence of vehicle or T0901317 alone or in combination with T0901317 and GSK864 (MCE, HY-19540). The cells were

lysed, and lipids were extracted with hexane-isopropanol solution (hexane: isopropanol (v:v) =3:2), evaporated and resuspended in toluene. Radioactivity was measured in scintillation fluid.

Metabolic flux analysis

AML12 cells were seeded in 6-well plates with unlabeled DMEM/F12 medium, and the media were removed when the cells had grown to 60% confluence. The cells were subsequently washed twice with PBS. The medium was then replaced with glutamine-free DMEM/F12 medium supplemented with 2.5 mM [U-^{13}C]glutamine (Sigma, 605166), 10% dialyzed FBS, 1% penicillin streptomycin, dexamethasone and ITS. After 24 h of labeling, the cells were treated with vehicle, T0901317 alone or T0901317 combined with GSK864 for 24 h. Afterward, the cells were washed with PBS. For glutamine-to-fatty acid flux analysis, the cells were collected in a 1.5-mL centrifuge tube. Fatty acids were extracted with 1 mL of 90% MeOH and 0.3 M KOH. After vortexing for 10 s, the solution was transferred to a 4 mL glass bottle and heated at 80°C for 1 h. Samples were collected by adding 100 μL of formic acid and 1 mL of hexane, vortexing and transferring to HPLC glass bottles, followed by evaporation to dryness in a vacuum concentrator. The dry extracts were reconstituted in 200 μL of ACN:MeOH (1:1, v/v) per million cells and stored at −20°C prior to LC–MS analysis. LC–MS analysis was performed via a Vanquish UHPLC system (Thermo Fisher Scientific) and an Acquity UPLC BEH C18 column (2.1×100 mm, 1.75 μm; Waters). For glutamine-to-citrate flux analysis, the cells were placed on

ice and extracted with 40 μl of MeOH:ACN:H_2O (2:2:1, v/v/v) mixture. The cells were scraped from the plate, transferred to a 1.5-mL centrifuge tube and incubated on ice for 10 minutes. The samples were vortexed for 30 seconds and centrifuged at 14000 rpm for 20 minutes at 4°C. The supernatants were transferred to a new 1.5-mL tube and centrifuged again at 14000 rpm for 20 minutes. The supernatants were ultimately transferred to HPLC glass bottles. LC separation was achieved via a Vanquish UHPLC system (Thermo Fisher Scientific) and a HILIC column (2.1×150 mm, 5 μm, HILICON). The raw data were converted to mzXML format via MSConver and processed via EI-MAVEN for peak detection, extraction, alignment and integration. Natural isotope abundance correction was applied via METLAB. The enrichment of lipogenic acetyl-CoA was calculated with FAMetA software (www.fameta.es) (10).

Co-culture of AML12 cells and HSCs

AML12 cells in glutamine-free DMEM/F12 medium supplemented with FBS, PS, dexamethasone and ITS were seeded in 12-well plates. At the same time, HSCs in glutamine-free DMEM supplemented with FBS and PS were seeded into 24 mm tissue culture transwell inserts (Corning, 3412) and incubated for 24 h. When the AML12 cells were 70% confluent, the cells were treated with vehicle or T0901317, and the HSCs containing inserts were transferred to AML12 cell-seeded plates. The cells were co-cultured for 24 h at 37°C, after which the HSCs were collected for analysis of gene expression. The experiments were performed in triplicate.

FIGURES

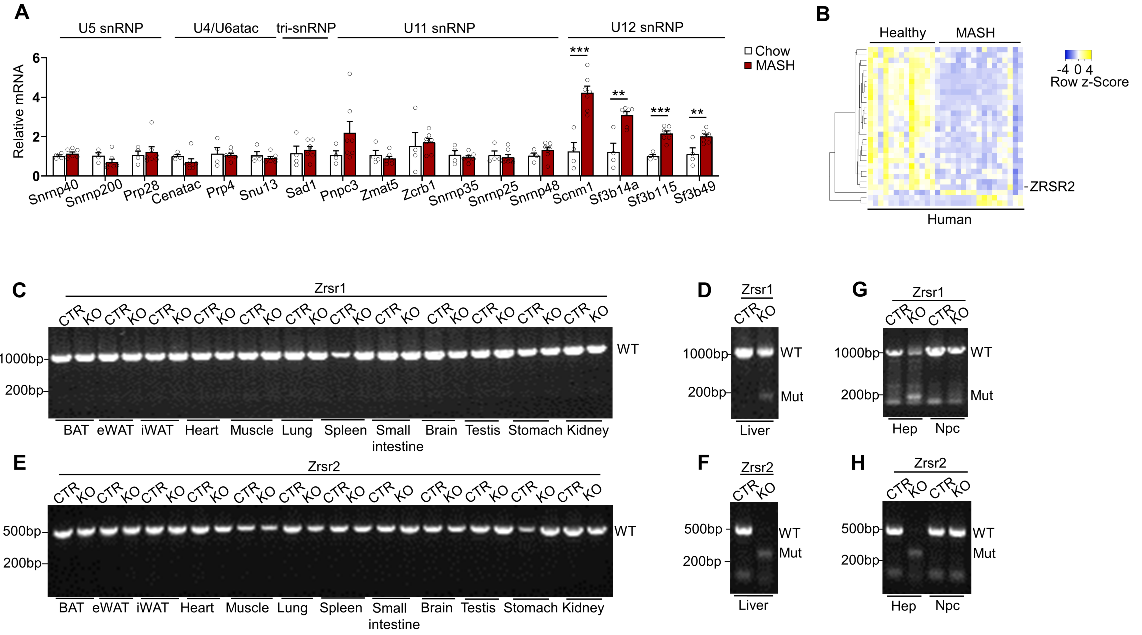

Figure S1. Hepatic expression of minor intron splicing factors and snRNAs under MASH condition and the genotyping of Zrsr1 and Zrsr2 double-deficient mice.

(**A**) QPCR analysis of minor intron splicing factors and snRNAs in the livers of mice fed chow-diet (*n*=4) or CDA-HFD (MASH diet, *n*=7) for 6 months. (**B**) Heatmap showing the gene expression of RNA binding proteins (RBPs), including ZRSR2, in the livers of healthy individuals and MASH patients (GSE126848). (**C**-**F**) *Zrsr1* (**C**-**D**) and *Zrsr2* (**E**-**F**) genotyping of BAT, eWAT, iWAT, heart, muscle, lung, spleen, small intestine, brain, testis, stomach, kidney (**C**, **E**) and liver (**D**, **F**) from AAV-control- (CTR) and AAV-sgRNA-injected Cas9-Tg mice (ZLKO). (**G**-**H**) *Zrsr1* (**G**) and *Zrsr2* (**H**) genotyping of isolated hepatocytes (Hep) and hepatic nonparenchymal cells (Npc) from the livers of CTR and ZLKO mice. The data are presented as the mean ± SEM. **p < 0.01, ***p < 0.001 by two-tailed unpaired Student’s t-test (A).

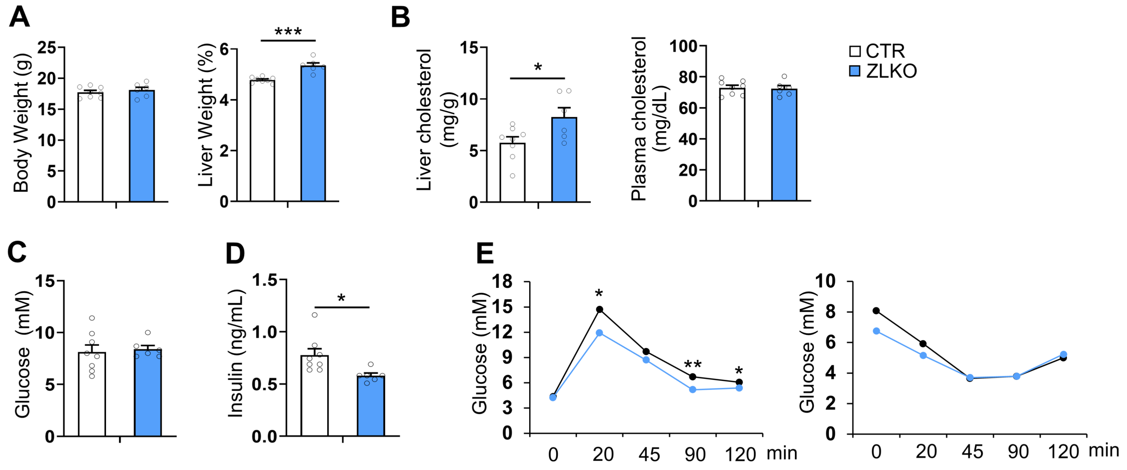

Figure S2. Metabolic phenotypes of Zrsr1 and Zrsr2 double-deficient mice after chow-diet feeding.

(**A**) Body weights (left) and percentage of liver weights (right) of CTR (*n*=8) and ZLKO (*n*=6) mice after chow diet feeding for 1 week. (**B**) Liver (left) and plasma (right) cholesterol contents in CTR and ZLKO mice on chow diet. (**C**-**D**) Plasma glucose (**C**) and insulin (**D**) levels in liver tissues from CTR and ZLKO mice. (**E**) GTT and ITT assay in the CTR (*n*=8) and ZLKO (*n*=6) mice after chow diet feeding. The data are presented as the mean ± SEM. *p<0.05, **p < 0.01, ***p < 0.001 by two-tailed unpaired Student's t-test (A-D), by two-way ANOVA with multiple comparisons (E).

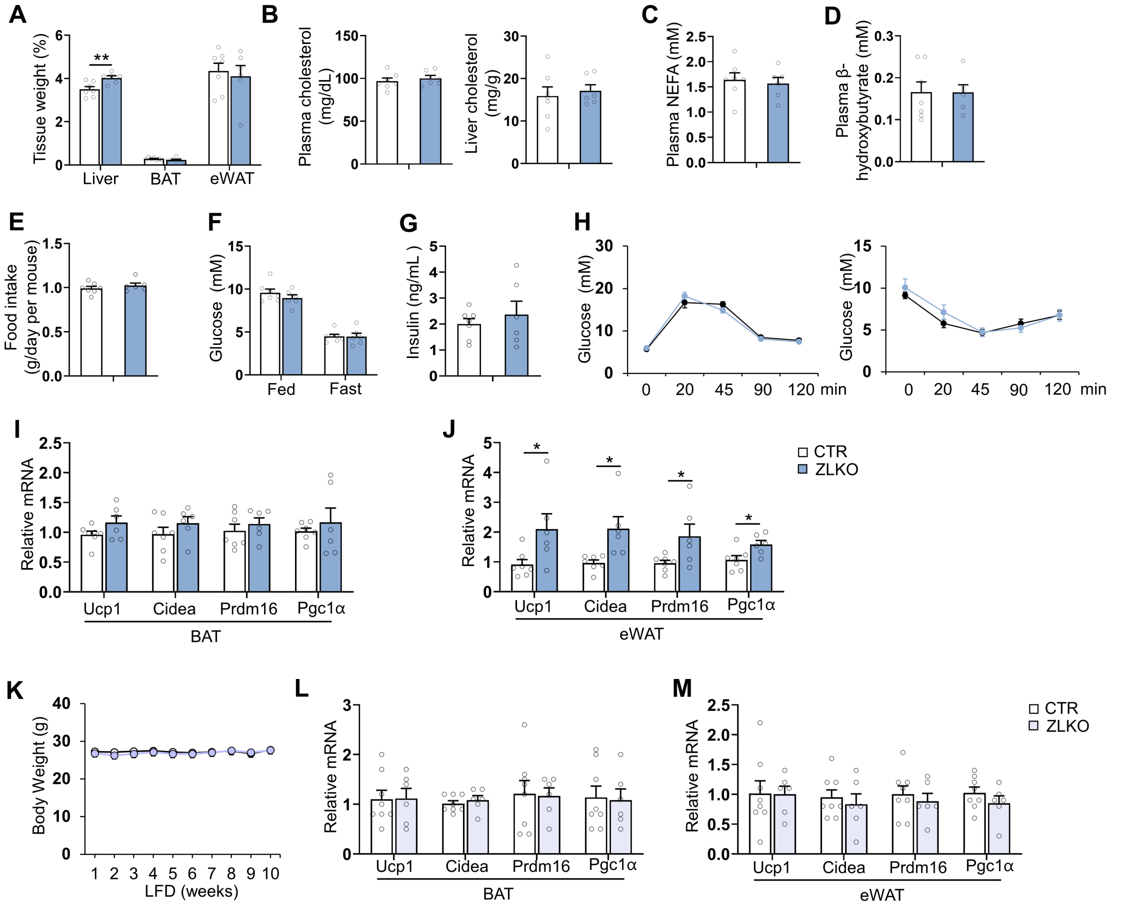

Figure S3. Metabolic and thermogenic phenotypes of Zrsr1 and Zrsr2 double-deficient mice in obese and lean conditions.

(**A**) Percentage of tissue weights of CTR (*n*=7) and ZLKO (*n*=6) mice fed high-fat diet (HFD) for 10 weeks. (**B**) Plasma (left) and liver (right) cholesterol contents in CTR and ZLKO mice after HFD feeding. (**C**-**D**) Plasma non-esterified fatty acid (NEFA, **C**) and β-hydroxybutyrate levels (**D**) in CTR and ZLKO mice after HFD feeding. (**E**) Food intake of CTR and ZLKO mice after HFD feeding. (**F**-**G**) Plasma glucose (**F**) and insulin (**G**) levels in the livers of CTR and ZLKO mice fed HFD. (**H**) GTT and ITT assays in the CTR and ZLKO mice after HFD feeding. (**I**-**J**) QPCR analysis of the expression of thermogenic genes in the BAT (**I**) and eWAT (**J**) of CTR and ZLKO mice after HFD feeding. (**K**) Body weights curve of CTR (*n*=8) and ZLKO (*n*=6) mice fed low-fat diet (LFD) for 10 weeks. (**L**-**M**) QPCR analysis of the expression of thermogenic genes in the BAT (**L**) and

eWAT (**M**) of CTR and ZLKO mice after LFD feeding. The data are presented as the mean ± SEM. *$p<0.05$, **$p < 0.01$ by two-tailed unpaired Student's t-test (A-G, I-J, and L-M), by two-way ANOVA with multiple comparisons (H and K).

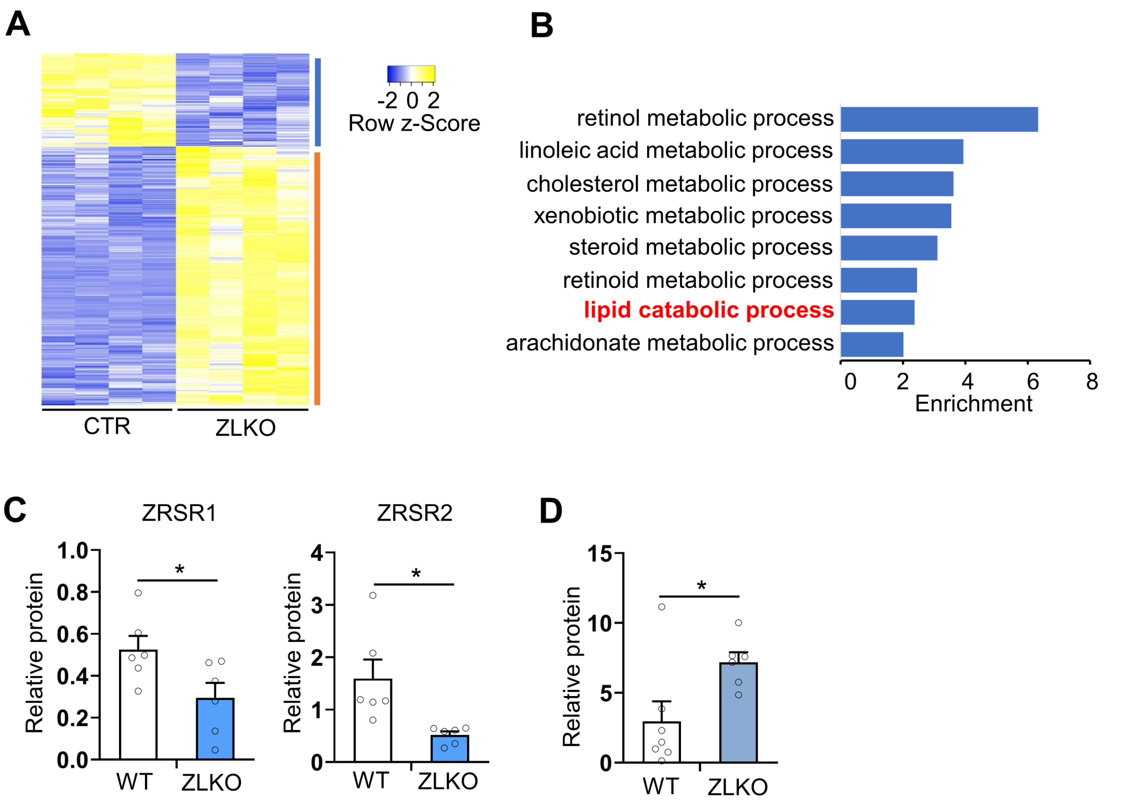

Figure S4. The expression profile and enriched pathways of differential expressed genes in ZLKO mice compared to CTR after chow-diet feeding and the quantification of ZRSR1 and ZRSR2 and cleaved SREBP1c levels. (**A**) Heatmap showing significant upregulated and downregulated >1.4-fold genes in the livers of ZLKO (*n*=4) livers compared to those in CTR (*n*=4) after chow-diet feeding for 1 week. (**B**) Gene Ontology analysis showing enriched pathways associated with genes downregulated in ZLKO mice on chow diet. (**C**) The relative density of the western blot bands of ZRSR1 and ZRSR2 that is normalized to HSP90, related to Figure 2F. (**D**) The relative density of the western blot bands of cleaved SREBP1c that is normalized to HSP90, related to Figure 2I. The data are presented as the mean ± SEM. *p<0.05 by two-tailed unpaired Student's t-test (C-D).

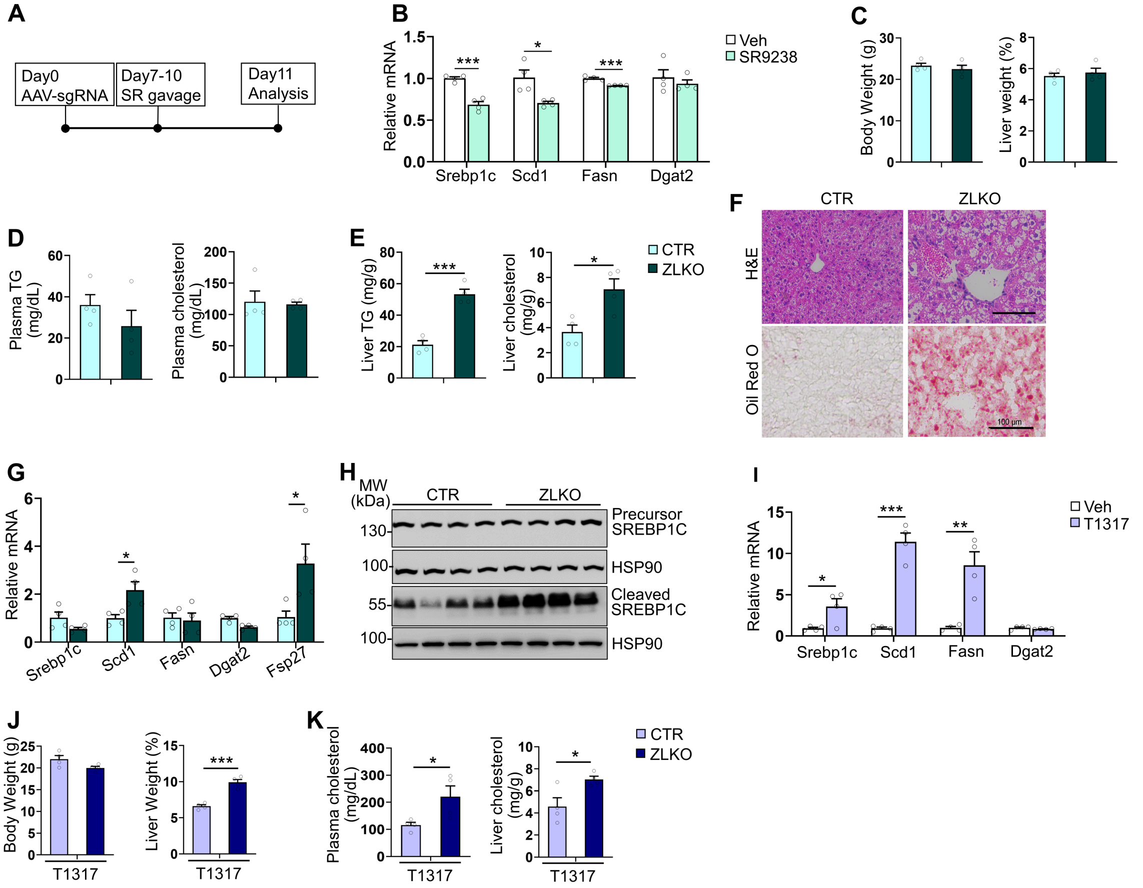

Figure S5. Metabolic phenotypes of Zrsr1 and Zrsr2 double-deficient mice upon LXR suppression or activation.

(**A**) Diagram of the study design. (**B**) QPCR analysis of lipogenesis-associated genes in the livers of wild-type mice receiving oral gavage of vehicle (Veh, *n*=4) or 20 mg/kg/day SR9238 (SR, *n*=4) for 4 days. (**C**) Body weights and percentage of liver weights of CTR (*n*=4) and ZLKO (*n*=4) mice after SR treatment for 4 days. (**D-E**) Plasma (**D**) and liver (**E**) triglyceride (TG) and cholesterol contents of CTR and ZLKO mice after SR treatment. (**F**) Hematoxylin and eosin (H&E) staining and Oil Red O staining (scale bar=100 μm) of livers from CTR and ZLKO mice after SR treatment. (**G**) QPCR analysis of lipogenesis-associated genes in the livers of CTR and ZLKO mice after SR treatment. (**H**) Western blot analyses of liver lysates from CTR and ZLKO mice after SR treatment. (**I**) QPCR analysis of lipogenesis-associated genes in the livers of wild type mice receiving oral gavage of vehicle (Veh, *n*=4) or 25 mg/kg/day T0901317 (T1317, *n*=4) for 4 days.

(**J**) Body weights and percentages of liver weights of CTR (*n*=4) and ZLKO (*n*=4) mice after T1317 treatment. (**K**) Plasma (left) and liver (right) cholesterol contents of CTR and ZLKO mice after T1317 treatment. The data are presented as the mean ± SEM. *$p<0.05$, **$p < 0.01$, ***$p < 0.001$ by two-tailed unpaired Student's t-test (B-E, G, and I-K).

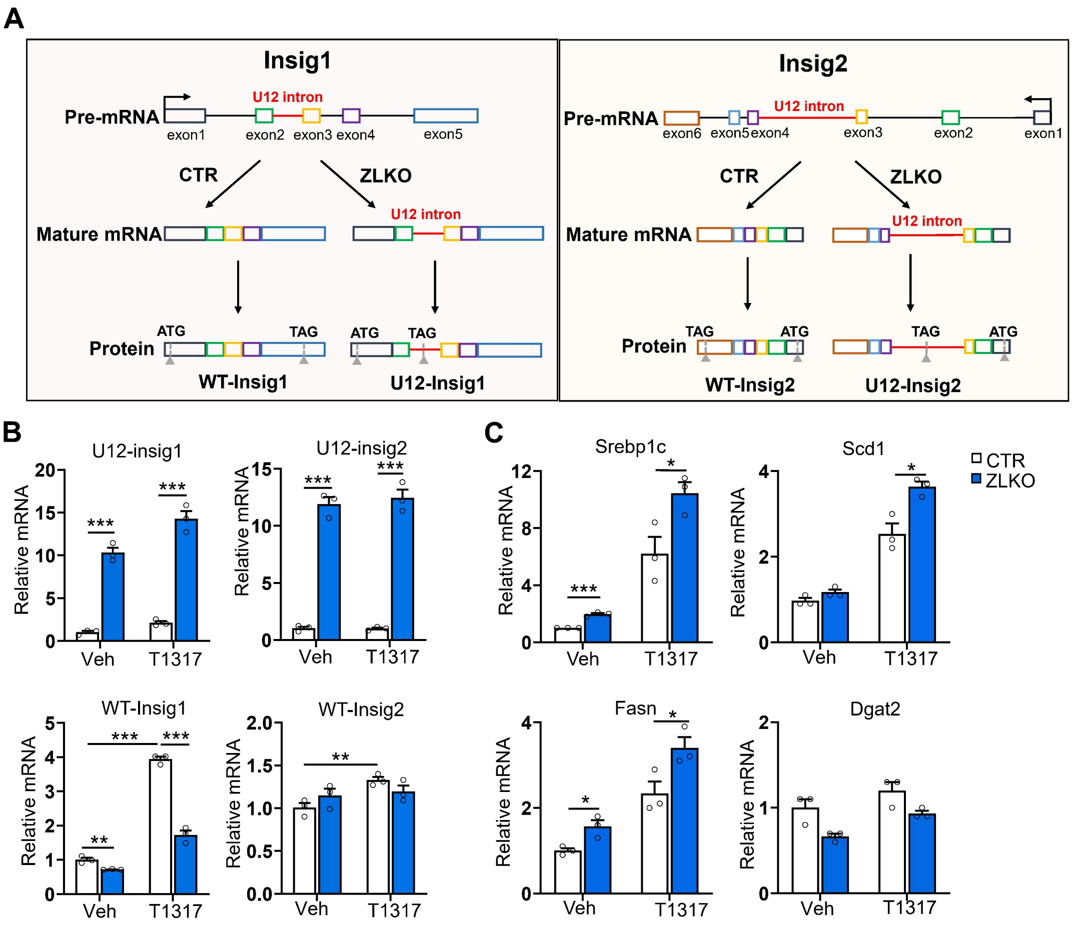

Figure S6. A diagram depicting the minor intron retention of *Insig1* and *Insig2* and depletion of Zrsr1 and Zrsr2 in primary hepatocytes induces *de novo* lipogenic gene expression and minor intron retention of *Insig1* and *Insig2*.

(**A**) Diagram depicting the minor intron retention of *Insig1* and *Insig2* genes in the livers of ZLKO mice results in the early termination of translation of Insig1 and Insig2. (**B**) QPCR analysis of minor (U12) intron expression of Insig1 and Insig2 and the mRNA levels of wild-type (WT) Insig1 and Insig2 in primary hepatocytes (*n*=3) isolated from CTR and ZLKO livers treated with vehicle (Veh) or T0901317 (T1317, 5 μM) for 24 h. (**C**) QPCR analysis of lipogenic genes in primary hepatocytes (*n*=3) isolated from CTR and ZLKO livers treated with Veh or T1317 (5 μM) for 24 h. The data in B-C are presented as the means ± SEM. *p<0.05, **p < 0.01, ***p < 0.001, two-tailed unpaired Student's t-test.

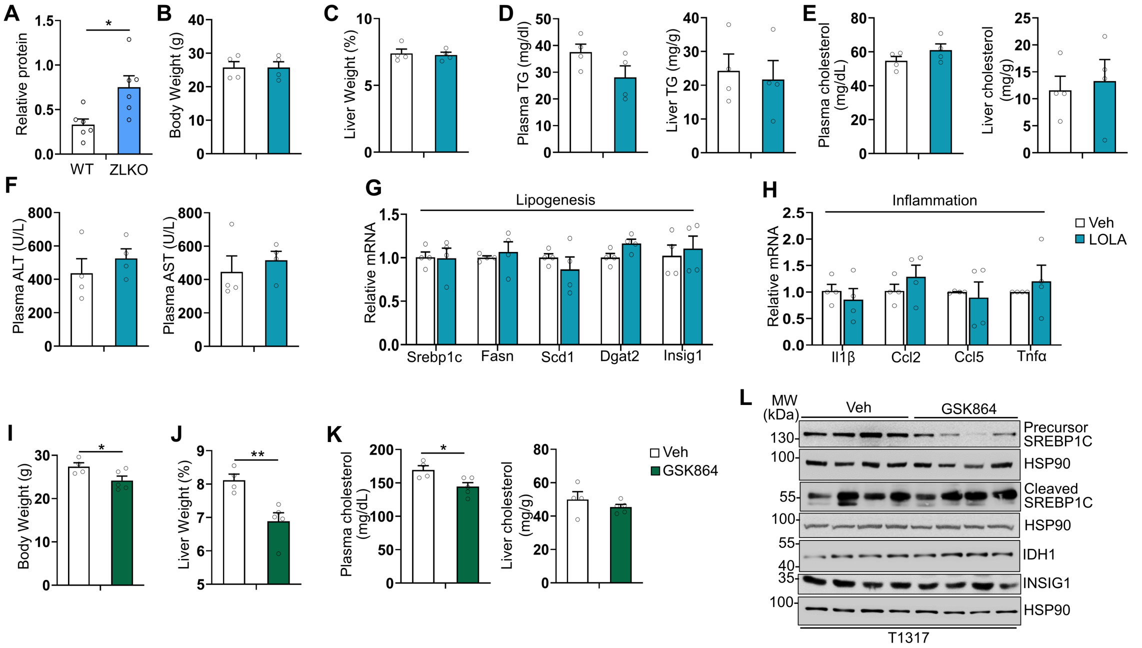

Figure S7. The quantification of IDH1 level and metabolic phenotypes after clearance of hepatic ammonia and inhibition of IDH1 activity in Zrsr1 and Zrsr2 double-deficient mice upon LXR activation.

(**A**) The relative density of the western blot bands of IDH1 that is normalized to HSP90, related to Figure 4O. (**B**-**C**) Body weights (**B**) and percentage of liver weights (**C**) of ZLKO mice daily injected with saline (*n*=4) or 2 g/kg L-ornithine-aspartate (LOLA, *n*=4) for 17 days and then combined with an oral administration of T0901317 (T1317, 25mg/kg/day) for 4 days. (**D**-**E**) Plasma and liver triglyceride (TG) (**D**) and cholesterol (**E**) levels in Veh- and LOLA-treated ZLKO mice combined with T1317 treatment. (**F**) Plasma ALT and AST levels in Veh- and LOLA-treated ZLKO mice combined with T1317 treatment. (**G**-**H**) QPCR analysis of hepatic genes involved in lipogenesis and inflammation in Veh- and LOLA-treated ZLKO mice combined with T1317 treatment. (**I**-**J**) Body weights (**I**) and percentage of liver weights (**J**) of ZLKO mice intraperitoneally injected with Veh (*n*=4) or GSK864 (75 mg/kg/day, *n*=5) combined with an oral administration of T1317 (25 mg/kg/day) for 4 days. (**K**) Plasma and liver cholesterol content in Veh- and GSK864-treated ZLKO mice combined with T1317 treatment. (**L**) Immunoblotting of liver lysates from Veh- and GSK864-treated ZLKO mice

combined with T1317 treatment. The data are presented as the means ± SEM. *$p<0.05$, **$p < 0.01$ by two-tailed unpaired Student's t-test (A-K).

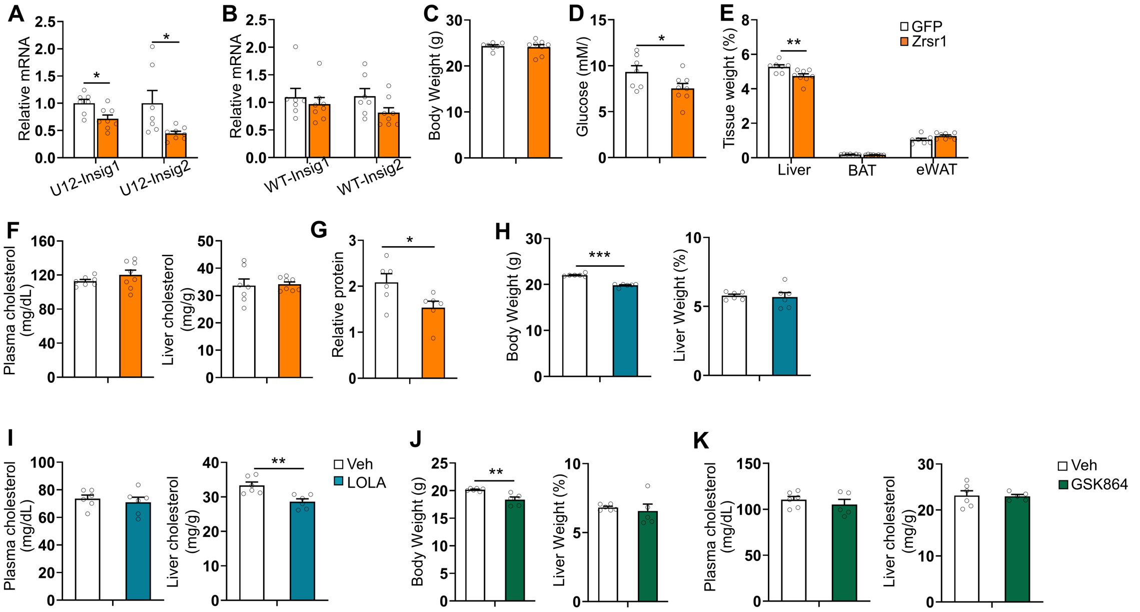

Figure S8. Metabolic phenotypes in mice after restoring minor intron splicing activity or suppressing IDH1-ammonia axis upon MASH diet feeding and the quantification of IDH1 level

(**A**-**B**) QPCR analysis of minor (U12) introns in Insig1 and Insig2 (**A**) and the mRNA levels of wild type (WT) Insig1 and Insig2 (**B**) in the livers of mice injected with AAV-TBG-GFP (GFP, *n*=7) or AAV-TBG-Zrsr1 (Zrsr1, *n*=8) after CDA-HFD (MASH diet) feeding for 8 weeks. (**C**-**E**) Body weights (**C**), blood glucose level (**D**) and percentage of tissue weights (**E**) were measured in GFP- and Zrsr1-expressing mice. (**G**) The relative density of the western blot bands of IDH1 that is normalized to HSP90, related to Figure 6F. (**F**) Plasma (left) and liver (right) cholesterol content in GFP- and Zrsr1-expressing mice. (**H**) Body weights (left) and percentage of liver weights (right) in wild type mice daily gavaged with saline (*n*=6) or 2 g/kg L-ornithine-aspartate (LOLA, *n*=6) and both fed on MASH diet for 8 weeks. (**I**) Plasma (left) and liver (right) cholesterol content in Veh- and LOLA-treated wild type mice after MASH diet feeding. (**J**) Body weights (left) and percentage of liver weights (right) in wild type mice daily intraperitoneally injected with vehicle (Veh, *n*=6) or 75 mg/kg GSK864 (*n*=5) and both fed on MASH diet for 8 weeks. (**K**) Plasma (left) and liver (right) cholesterol content in Veh- and GSK864-treated wild type mice after MASH diet feeding. The data are presented

as the mean ± SEM. *$p<0.05$, **$p < 0.01$, ***$p < 0.001$, two-tailed unpaired Student's t-test (A-K).

References

1. Lin J, Wu PH, Tarr PT, Lindenberg KS, St-Pierre J, Zhang CY, et al. Defects in adaptive energy metabolism with CNS-linked hyperactivity in PGC-1alpha null mice. *Cell.* 2004;119(1):121-35.
2. Ma D, Molusky MM, Song J, Hu CR, Fang F, Rui C, et al. Autophagy deficiency by hepatic FIP200 deletion uncouples steatosis from liver injury in NAFLD. *Molecular endocrinology.* 2013;27(10):1643-54.
3. Gutiérrez-de-Juan V, López de Davalillo S, Fernández-Ramos D, Barbier-Torres L, Zubiete-Franco I, Fernández-Tussy P, et al. A morphological method for ammonia detection in liver. *PLoS One.* 2017;12(3):e0173914.
4. Li S, Liu C, Li N, Hao T, Han T, Hill DE, et al. Genome-wide coactivation analysis of PGC-1alpha identifies BAF60a as a regulator of hepatic lipid metabolism. *Cell Metab.* 2008;8(2):105-17.
5. Dobin A, Davis CA, Schlesinger F, Drenkow J, Zaleski C, Jha S, et al. STAR: ultrafast universal RNA-seq aligner. *Bioinformatics.* 2013;29(1):15-21.
6. Anders S, Pyl PT, and Huber W. HTSeq--a Python framework to work with high-throughput sequencing data. *Bioinformatics.* 2015;31(2):166-9.
7. Moyer DC, Larue GE, Hershberger CE, Roy SW, and Padgett RA. Comprehensive database and evolutionary dynamics of U12-type introns. *Nucleic Acids Res.* 2020;48(13):7066-78.

8. Love MI, Huber W, and Anders S. Moderated estimation of fold change and dispersion for RNA-seq data with DESeq2. *Genome biology.* 2014;15(12):550.

9. Pertea M, Pertea GM, Antonescu CM, Chang TC, Mendell JT, and Salzberg SL. StringTie enables improved reconstruction of a transcriptome from RNA-seq reads. *Nat Biotechnol.* 2015;33(3):290-5.

10. Alcoriza-Balaguer MI, García-Cañaveras JC, Benet M, Juan-Vidal O, and Lahoz A. FAMetA: a mass isotopologue-based tool for the comprehensive analysis of fatty acid metabolism. *Brief Bioinform.* 2023;24(2).

Table S1. Clinical characteristics of the healthy individuals and MASH patients

Healthy individuals#	Gender	Age(year)	NAS
1	Male	26	0
2	Female	30	0
3	Female	29	0
4	Female	34	0
5	Female	31	0
6	Male	27	0
7	Female	29	0
8	Male	31	0

MASH Patients #	Gender	Age (year)	ALT (U/L)	AST (U/L)	TG (mmol/L)	TCHO (mmol/L)	NAS
1	Female	61	42	25	2.57	6.08	5
2	Female	58	59	42	6.07	6	6
3	Female	65	24	22	3.97	4.26	6
4	Male	27	194	89	5.16	7.33	4
5	Male	33	26	10	1.25	4.17	5
6	Male	38	22	20	1.57	4.63	4

Table S2. U12 intron reads in U12 intron-containing genes of CTR and ZLKO mice

GENE ID	NAME	CTR1	CTR2	CTR3	CTR4	ZLKO1	ZLKO2	ZLKO3	ZLKO4
ENSMUSG00000047879	Usp14	0	0	0	0	24	24	22	20
ENSMUSG00000062234	Gak	0	0	0	0	25	27	31	30
ENSMUSG00000032220	Myo1e	0	0	0	0	111	97	124	94
ENSMUSG00000063358	Mapk1	0	0	0	0	151	107	156	141
ENSMUSG00000031242	Chmp1b2	0	0	0	0	160	114	162	135
ENSMUSG00000024231	Cul2	0	0	0	0	327	250	372	351
ENSMUSG00000055067	Smyd3	0	0	0	0	36	29	47	39
ENSMUSG00000039100	Marchf6	0	0	0	0	19	22	15	25
ENSMUSG00000051177	Plcb1	0	0	0	0	8	13	8	9
ENSMUSG00000004865	Srpk1	0	0	0	0	81	43	80	76
ENSMUSG00000029387	Gtf2h3	0	0	0	0	38	22	40	27
ENSMUSG00000040264	Gbp2b	0	0	0	0	8	6	9	12
ENSMUSG00000001173	Ocrl	0	0	0	0	14	9	7	11
ENSMUSG00000024095	Hnrnpll	0	0	0	0	35	22	17	26
ENSMUSG00000022443	Myh9	0	0	0	0	6	5	10	8
ENSMUSG00000038095	Sbno1	0	0	0	0	16	9	18	11
ENSMUSG00000026187	Xrcc5	0	0	0	0	10	7	12	6
ENSMUSG00000038417	Fig4	0	0	0	0	6	6	7	3
ENSMUSG00000030982	Vps35l	0	0	0	0	61	45	59	28
ENSMUSG00000059273	Zc3h4	0	0	0	0	5	3	7	6
ENSMUSG00000028318	Polr1e	0	0	0	0	6	4	8	4
ENSMUSG00000079317	Trappc2	0	0	0	0	5	8	5	10
ENSMUSG00000005899	Smpd4	0	0	0	0	18	7	15	17
ENSMUSG00000042590	Ipo11	0	0	0	0	4	5	2	5
ENSMUSG00000009470	Tnpo1	0	0	0	0	13	13	12	24
ENSMUSG00000033396	Spg11	0	0	0	0	22	9	13	15
ENSMUSG00000044447	Dock5	0	0	0	0	6	10	9	4
ENSMUSG00000035704	Alg8	0	0	0	0	1	2	2	1
ENSMUSG00000031095	Cul4b	0	0	0	0	2	4	2	4
ENSMUSG00000040325	Dcaf1	0	0	0	0	2	2	4	2
ENSMUSG00000033382	Trappc8	0	0	0	0	9	3	7	6
ENSMUSG00000079362	Gm43302	0	0	0	0	16	6	17	11
ENSMUSG00000026585	Kifap3	0	0	0	0	3	3	1	2
ENSMUSG00000046873	Mbtps2	0	0	0	0	11	6	13	5
ENSMUSG00000002413	Braf	0	0	0	0	5	4	8	11
ENSMUSG00000020895	Tmem107	0	0	0	0	7	2	5	4
ENSMUSG00000000028	Cdc45	0	0	0	0	4	4	3	1
ENSMUSG00000025188	Hps1	0	0	0	0	8	9	4	3
ENSMUSG00000073792	Alg6	0	0	0	0	8	3	5	3
ENSMUSG00000052798	Nup107	0	0	0	0	5	9	11	3
ENSMUSG00000022960	Donson	0	0	0	0	1	2	1	3
ENSMUSG00000073733	Cplane2	0	0	0	0	1	2	4	2
ENSMUSG00000060681	Slc9a6	0	0	0	0	9	3	3	9
ENSMUSG00000029407	Uso1	0	0	0	0	2	6	8	13
ENSMUSG00000022558	Mroh1	0	0	0	0	6	1	3	3
ENSMUSG00000017765	Slc12a4	0	0	0	0	9	3	2	5
ENSMUSG00000015968	Cacna1d	0	0	0	0	0	1	1	1
ENSMUSG00000022537	Tmem44	0	0	0	0	1	1	0	1
ENSMUSG00000063870	Chd4	0	0	0	0	9	8	22	4
ENSMUSG00000010825	Grid2ip	0	0	0	0	2	2	1	0
ENSMUSG00000034438	Gbp8	0	0	0	0	2	0	2	1
ENSMUSG00000041879	Ipo9	0	0	0	0	1	0	2	1
ENSMUSG00000079363	Gbp4	0	0	0	0	1	2	1	0
ENSMUSG00000024066	Xdh	0	1	0	0	142	88	129	112
ENSMUSG00000026353	Ubxn4	1	1	2	1	446	320	472	448
ENSMUSG00000028270	Gbp2	0	1	0	0	85	68	69	52

ENSMUSG00000028653	Trit1	1	3	2	2	518	356	671	596
ENSMUSG00000024066	Xdh	0	0	0	1	56	53	75	60
ENSMUSG00000039456	Morc3	1	1	1	1	251	160	315	250
ENSMUSG00000063558	Aox1	0	0	1	0	59	39	55	41
ENSMUSG00000040282	Cdin1	0	0	1	0	47	41	56	43
ENSMUSG00000017756	Slc12a7	0	3	1	2	285	174	303	295
ENSMUSG00000021629	Slc30a5	0	0	0	2	89	50	90	106
ENSMUSG00000005262	Ufd1	0	2	2	5	383	263	425	433
ENSMUSG00000026491	Ahctf1	1	0	1	0	88	80	74	82
ENSMUSG00000031696	Vps35	2	0	0	1	113	77	128	117
ENSMUSG00000026349	Ccnt2	6	1	0	1	249	179	324	305
ENSMUSG00000066900	Suds3	1	1	0	1	78	55	112	116
ENSMUSG00000062169	Cnih4	1	0	1	0	46	58	70	65
ENSMUSG00000016477	E2f3	1	0	0	1	65	50	58	65
ENSMUSG00000025240	Sacm1l	3	2	0	1	179	129	189	175
ENSMUSG00000026155	Smap1	0	1	1	0	48	42	65	65
ENSMUSG00000029016	Clcn6	0	0	1	0	44	25	28	12
ENSMUSG00000048170	Mcmbp	0	0	1	0	25	18	25	40
ENSMUSG00000031691	Tnpo2	2	0	2	0	118	71	121	103
ENSMUSG00000035770	Dync1li2	1	0	0	1	50	44	63	47
ENSMUSG00000039159	Ube2h	5	5	2	5	448	332	479	467
ENSMUSG00000027671	Actl6a	3	0	0	0	75	51	92	78
ENSMUSG00000042747	Krtcap2	2	3	0	0	112	108	140	86
ENSMUSG00000029104	Htt	0	0	1	0	21	19	24	24
ENSMUSG00000054237	Fra10ac1	3	0	4	3	212	196	251	214
ENSMUSG00000002748	Baz1b	1	4	1	1	146	124	149	181
ENSMUSG00000062627	Mysm1	0	0	0	1	16	12	28	29
ENSMUSG00000026470	Stx6	1	0	0	1	57	34	46	31
ENSMUSG00000044308	Ubr3	0	2	0	1	63	36	84	64
ENSMUSG00000030619	Eed	1	0	0	1	36	30	46	51
ENSMUSG00000017421	Zfp207	3	4	6	4	331	293	369	388
ENSMUSG00000017756	Slc12a7	2	4	6	3	359	224	291	314
ENSMUSG00000005373	Mlxipl	10	11	11	10	938	600	909	715
ENSMUSG00000040253	Gbp7	2	0	1	0	69	55	55	44
ENSMUSG00000026585	Kifap3	0	0	1	0	15	22	20	16
ENSMUSG00000030704	Rab6a	2	7	7	6	378	304	426	344
ENSMUSG00000046985	Tapt1	0	7	2	3	192	137	230	223
ENSMUSG00000036208	Nepro	0	0	1	0	18	11	15	21
ENSMUSG00000024833	Pola2	1	0	0	1	39	27	37	23
ENSMUSG00000018433	Nol11	0	0	0	3	37	39	52	58
ENSMUSG00000020017	Hal	47	39	24	31	2044	2825	2041	1479
ENSMUSG00000067150	Xpo5	0	1	0	0	14	10	16	18
ENSMUSG00000037720	Tmem33	11	7	8	5	477	334	481	462
ENSMUSG00000028089	Chd1l	0	1	0	2	55	35	46	32
ENSMUSG00000014547	Wdfy2	0	0	1	0	5	6	17	28
ENSMUSG00000032624	Eml4	0	1	2	0	38	26	52	49
ENSMUSG00000037461	Ints7	1	0	0	1	39	24	27	20
ENSMUSG00000027828	Ssr3	2	0	5	4	159	112	168	156
ENSMUSG00000020922	Lsm12	1	1	1	0	32	35	48	47
ENSMUSG00000034480	Diaph2	0	1	1	0	32	23	21	30
ENSMUSG00000032594	Ip6k1	10	13	6	2	456	280	488	392
ENSMUSG00000035954	Dock4	1	0	0	0	10	12	12	17
ENSMUSG00000021952	Xpo4	0	1	1	3	51	52	68	80
ENSMUSG00000079562	Maea	1	4	0	2	91	76	97	87
ENSMUSG00000058301	Upf1	0	0	2	1	30	26	43	51
ENSMUSG00000035673	Sbno2	0	1	1	1	31	39	42	37
ENSMUSG00000000374	Trappc10	0	1	0	0	7	14	14	12
ENSMUSG00000027981	Rnpc3	0	0	1	0	13	6	17	11

ENSMUSG00000034525	Ice1	2	1	1	1	44	59	58	67
ENSMUSG00000022369	Mtbp	0	0	0	1	12	10	8	15
ENSMUSG00000105096	Gbp10	1	0	0	0	10	10	19	6
ENSMUSG00000003721	Insig2	50	15	50	63	1615	1607	2604	2169
ENSMUSG00000001380	Hars1	0	2	0	1	26	33	32	43
ENSMUSG00000019988	Nedd1	5	6	1	3	174	128	187	179
ENSMUSG00000020780	Srp68	0	3	2	4	97	77	103	123
ENSMUSG00000032743	Katnip	0	0	1	1	21	19	23	25
ENSMUSG00000010936	Vac14	1	0	1	1	40	23	35	30
ENSMUSG00000018999	Slc35b4	1	0	1	1	38	17	33	38
ENSMUSG00000018442	Derl2	6	8	11	11	449	285	429	344
ENSMUSG00000028268	Gbp3	1	0	0	0	9	13	11	8
ENSMUSG00000064037	Gpn1	1	1	1	1	42	30	54	38
ENSMUSG00000052459	Atp6v1a	1	0	1	1	39	20	39	24
ENSMUSG00000059208	Hnrnpm	11	12	24	8	563	517	606	545
ENSMUSG00000030527	Crtc3	0	1	1	0	24	15	28	13
ENSMUSG00000028798	Eif3i	3	4	2	1	90	76	119	111
ENSMUSG00000018442	Derl2	5	7	2	4	218	102	215	174
ENSMUSG00000041769	Ppp2r2d	13	5	7	5	306	147	389	339
ENSMUSG00000026496	Parp1	1	1	0	1	26	26	33	32
ENSMUSG00000060681	Slc9a6	2	0	0	1	22	26	28	40
ENSMUSG00000030471	Zdhhc13	3	6	8	7	201	197	253	250
ENSMUSG00000022476	Polr3h	0	1	1	0	16	20	21	17
ENSMUSG00000028894	Inpp5b	1	4	1	1	51	62	78	68
ENSMUSG00000039456	Morc3	1	0	1	0	12	12	25	25
ENSMUSG00000038002	Cramp1	0	1	0	0	5	7	16	9
ENSMUSG00000019984	Med23	0	3	2	6	107	95	95	104
ENSMUSG00000013663	Pten	7	7	7	5	229	165	280	267
ENSMUSG00000010936	Vac14	0	0	0	1	12	11	5	8
ENSMUSG00000032435	Dync1li1	0	0	3	2	33	42	51	41
ENSMUSG00000037343	Taf2	4	2	4	1	102	68	104	92
ENSMUSG00000030739	Myh14	0	0	0	2	18	18	16	14
ENSMUSG00000014859	E2f4	1	0	0	0	7	4	10	12
ENSMUSG00000038949	Cnst	1	10	5	8	208	150	228	204
ENSMUSG00000055239	Kcmf1	4	1	1	2	75	54	69	64
ENSMUSG00000021258	Ccnk	2	1	2	1	56	43	55	42
ENSMUSG00000032870	Smap2	1	1	1	0	24	20	30	21
ENSMUSG00000016541	Atxn10	1	3	4	2	70	59	77	109
ENSMUSG00000028576	Ift74	1	0	1	0	17	12	17	15
ENSMUSG00000036099	Vezt	1	2	0	3	54	40	50	39
ENSMUSG00000021027	Ralgapa1	1	0	0	2	19	17	26	28
ENSMUSG00000073643	Wdfy1	4	3	2	1	75	46	113	64
ENSMUSG00000028187	Rpf1	13	11	10	7	323	259	326	306
ENSMUSG00000010392	Gosr1	11	12	12	10	373	231	373	345
ENSMUSG00000041528	Rnf123	4	4	1	1	79	63	90	61
ENSMUSG00000028447	Dctn3	3	3	7	5	119	123	148	132
ENSMUSG00000015757	Ppil4	0	3	3	4	84	60	68	77
ENSMUSG00000029686	Cul1	2	1	2	5	59	72	77	80
ENSMUSG00000020536	Llgl1	0	1	1	1	11	24	29	22
ENSMUSG00000028894	Inpp5b	0	1	0	1	13	20	13	11
ENSMUSG00000003161	Sri	6	8	4	8	176	179	219	160
ENSMUSG00000028330	Ncbp1	5	4	3	1	96	59	109	102
ENSMUSG00000032743	Katnip	2	1	1	2	55	31	51	30
ENSMUSG00000031232	Magt1	5	9	10	4	176	139	218	225
ENSMUSG00000040044	Orc3	3	6	5	2	116	83	99	122
ENSMUSG00000023764	Sfi1	3	3	3	4	77	70	111	83
ENSMUSG00000022020	Naa16	0	1	2	1	20	20	29	35
ENSMUSG00000031446	Cul4a	12	27	22	12	516	338	508	492

ENSMUSG00000036270	Edc4	1	0	0	2	12	17	22	25
ENSMUSG00000019808	Adat2	0	1	1	1	20	20	17	18
ENSMUSG00000020925	Ccdc43	3	5	7	4	132	98	122	119
ENSMUSG00000040472	Rabggta	1	0	4	3	49	42	49	54
ENSMUSG00000036810	Cnep1r1	1	3	4	5	86	64	71	92
ENSMUSG00000000915	Hip1r	0	0	1	0	6	4	11	3
ENSMUSG00000026509	Capn2	3	0	0	1	33	21	26	15
ENSMUSG00000075704	Txnrd2	5	8	5	6	128	128	171	125
ENSMUSG00000021140	Pcnx	1	0	0	0	5	4	6	8
ENSMUSG00000022774	Ncbp2	1	2	2	0	32	23	37	22
ENSMUSG00000025133	Ints4	5	8	4	3	136	100	114	105
ENSMUSG00000028030	Tbck	2	1	2	3	42	36	47	56
ENSMUSG00000047554	Tmem41b	7	11	5	10	213	163	194	174
ENSMUSG00000026319	Relch	9	3	7	6	129	113	156	158
ENSMUSG00000028218	Cibar1	3	1	1	2	47	29	40	39
ENSMUSG00000031105	Slc25a14	0	0	0	1	5	6	5	6
ENSMUSG00000032754	Slc8b1	2	1	1	1	37	29	28	16
ENSMUSG00000058979	Hdhd5	13	8	17	17	279	270	339	306
ENSMUSG00000037674	Rfx7	1	0	0	2	15	15	18	17
ENSMUSG00000021076	Actr10	1	1	0	3	28	24	31	25
ENSMUSG00000030061	Uba3	7	5	2	4	102	60	108	114
ENSMUSG00000036270	Edc4	1	2	1	2	36	32	33	26
ENSMUSG00000064127	Med14	0	0	0	1	6	6	3	6
ENSMUSG00000040865	Ino80d	0	0	1	0	8	4	1	8
ENSMUSG00000060681	Slc9a6	0	0	0	1	10	1	4	6
ENSMUSG00000024942	Capn1	0	0	0	1	2	3	5	11
ENSMUSG00000045294	Insig1	32	42	42	34	1138	722	872	408
ENSMUSG00000070426	Rnf121	5	5	6	2	98	57	111	100
ENSMUSG00000071647	Eml3	0	2	1	1	15	17	30	18
ENSMUSG00000058486	Wdr91	0	1	0	1	7	10	15	8
ENSMUSG00000021222	Dcaf4	0	1	0	0	9	4	5	2
ENSMUSG00000022052	Ppp2r2a	0	3	0	0	14	13	16	16
ENSMUSG00000068921	Dap3	0	1	1	3	21	24	32	20
ENSMUSG00000041180	Hectd2	1	0	1	2	13	15	29	20
ENSMUSG00000003575	Crtc1	0	1	0	0	3	7	5	4
ENSMUSG00000031858	Mau2	0	0	1	0	8	3	5	3
ENSMUSG00000062604	Srpk2	6	7	6	9	144	113	138	132
ENSMUSG00000030751	Psma1	11	13	12	9	224	191	227	189
ENSMUSG00000024118	Tedc2	2	1	2	3	29	34	37	47
ENSMUSG00000031197	Vbp1	1	4	2	2	44	31	45	44
ENSMUSG00000027201	Myef2	0	0	1	0	7	0	6	5
ENSMUSG00000055912	Tmem150a	12	7	20	6	224	198	228	153
ENSMUSG00000020017	Hal	8	13	10	8	171	191	174	158
ENSMUSG00000022365	Derl1	8	5	5	9	132	95	121	127
ENSMUSG00000072825	Cep170b	1	1	0	0	5	7	13	10
ENSMUSG00000040565	Btaf1	1	2	3	1	31	24	31	36
ENSMUSG00000020537	Drg2	2	5	3	3	49	49	70	58
ENSMUSG00000003033	Ap1m1	6	6	6	4	91	82	99	110
ENSMUSG00000040738	Ints8	1	0	2	0	14	7	16	15
ENSMUSG00000033632	AW55491[illegible]	0	1	2	0	12	12	9	19
ENSMUSG00000022992	Kansl2	10	3	7	10	126	98	154	140
ENSMUSG00000026664	Phyh	17	15	17	27	306	305	344	352
ENSMUSG00000047248	C2cd3	1	0	2	2	16	20	23	24
ENSMUSG00000015013	Trappc2l	0	2	4	1	33	26	27	30
ENSMUSG00000027185	Nat10	7	3	4	4	73	73	95	56
ENSMUSG00000020782	Llgl2	2	1	2	1	25	24	24	23
ENSMUSG00000092021	Gbp11	1	1	1	1	15	13	19	17
ENSMUSG00000024319	Vps52	1	0	0	4	23	19	22	16

ENSMUSG00000025047	Pdcd11	0	0	0	1	3	7	4	2
ENSMUSG00000056536	Pign	4	4	2	5	60	46	70	57
ENSMUSG00000003099	Ppp5c	2	1	5	5	44	42	57	57
ENSMUSG00000045996	Polr2k	4	2	1	2	32	26	38	41
ENSMUSG00000006736	Tspan31	18	20	40	25	294	371	399	498
ENSMUSG00000020900	Myh10	0	1	0	0	6	6	1	2
ENSMUSG00000028096	Gpr89	3	2	8	5	72	55	82	60
ENSMUSG00000039844	Rapgef1	3	5	7	6	96	45	87	78
ENSMUSG00000035762	Tmem161b	12	10	13	21	189	173	236	213
ENSMUSG00000021936	Mapk8	3	4	2	9	76	60	57	61
ENSMUSG00000068114	Ccdc134	4	4	7	5	76	65	75	59
ENSMUSG00000022621	Rabl2	1	1	1	1	16	15	11	13
ENSMUSG00000029790	Cep41	1	1	0	0	7	5	8	7
ENSMUSG00000033486	Catsper2	0	2	0	0	7	5	7	8
ENSMUSG00000041096	Tspyl2	1	2	2	3	35	25	28	18
ENSMUSG00000033813	Tcea1	7	8	14	8	96	110	150	128
ENSMUSG00000032399	Rpl4	2	7	7	4	60	54	62	83
ENSMUSG00000062822	4833420G1	10	14	9	7	149	115	128	123
ENSMUSG00000034265	Zdhhc14	2	1	5	0	28	25	27	23
ENSMUSG00000024974	Smc3	5	3	3	3	45	42	42	48
ENSMUSG00000024319	Vps52	3	3	4	4	35	43	46	51
ENSMUSG00000031434	Morc4	1	0	0	1	12	7	2	4
ENSMUSG00000039356	Exosc2	2	1	3	5	33	32	38	33
ENSMUSG00000028010	Gar1	0	2	1	0	7	10	10	10
ENSMUSG00000079469	Pigb	0	0	3	4	15	22	32	16
ENSMUSG00000058325	Dock1	0	0	1	0	0	4	5	3
ENSMUSG00000021952	Xpo4	2	0	0	2	9	9	15	14
ENSMUSG00000030603	Psmc4	5	4	2	2	37	40	37	38
ENSMUSG00000005774	Rfx5	1	0	2	0	12	8	11	4
ENSMUSG00000031839	Hsbp1	7	12	3	6	68	77	94	86
ENSMUSG00000017188	Coa3	3	6	7	11	68	75	76	92
ENSMUSG00000031516	Dctn6	4	7	6	7	69	61	75	71
ENSMUSG00000027411	Vps16	0	0	3	1	12	10	12	10
ENSMUSG00000032328	Tmem30a	56	36	49	53	506	444	646	534
ENSMUSG00000030555	Ttc23	0	4	2	8	43	20	48	41
ENSMUSG00000001794	Capns1	2	4	5	1	31	33	38	28
ENSMUSG00000025157	Zdhhc16	3	3	5	1	32	24	39	35
ENSMUSG00000028999	Rint1	3	1	6	9	46	39	68	50
ENSMUSG00000002342	Tmem161a	2	4	5	7	68	24	52	47
ENSMUSG00000071037	Camkmt	3	1	1	1	16	18	18	11
ENSMUSG00000028677	Rnf220	1	0	0	1	2	6	4	9
ENSMUSG00000022999	Lmbr1l	32	32	24	16	295	267	271	257
ENSMUSG00000031201	Brcc3	4	12	10	16	91	86	131	129
ENSMUSG00000026048	Ercc5	9	7	13	11	113	79	119	104
ENSMUSG00000008475	Arpc5	23	13	13	16	187	118	200	168
ENSMUSG00000022570	Gfus	1	3	1	1	16	13	15	18
ENSMUSG00000061286	Exosc5	3	1	0	2	17	18	12	15
ENSMUSG00000019790	Stxbp5	2	0	2	0	11	9	13	8
ENSMUSG00000024944	Arl2	5	3	14	5	65	58	77	66
ENSMUSG00000042308	Setd1a	1	3	1	0	7	9	19	14
ENSMUSG00000036636	Clcn7	0	1	1	2	11	5	12	11
ENSMUSG00000029599	Ddx54	0	0	1	1	5	4	5	5
ENSMUSG00000030662	Ipo5	0	0	1	1	4	4	6	5
ENSMUSG00000032078	Zpr1	5	4	13	19	69	47	132	135
ENSMUSG00000024474	Ik	12	10	19	17	130	123	138	147
ENSMUSG00000020366	Mapk9	11	21	20	23	162	172	174	169
ENSMUSG00000022100	Xpo7	1	0	1	2	11	8	6	11
ENSMUSG00000063273	Naa15	1	0	1	0	3	6	5	4

ENSMUSG00000017376	Nlk	3	5	5	6	36	20	61	53
ENSMUSG00000022663	Atg3	21	26	26	38	224	222	248	291
ENSMUSG00000031820	Babam1	10	15	8	10	84	79	116	89
ENSMUSG00000011960	Ccnt1	4	2	2	1	14	9	25	29
ENSMUSG00000024581	Napg	1	2	1	0	7	9	10	8
ENSMUSG00000020412	Ascc2	0	1	2	2	11	11	11	9
ENSMUSG00000028099	Polr3c	5	0	3	0	17	9	23	18
ENSMUSG00000033031	Cip2a	1	0	1	1	9	3	8	5
ENSMUSG00000060181	Slc35e3	14	10	16	13	103	80	139	118
ENSMUSG00000020553	Pctp	22	12	14	19	187	115	127	126
ENSMUSG00000025395	Prim1	2	1	1	0	10	10	8	5
ENSMUSG00000030761	Myo7a	3	1	1	4	21	20	20	13
ENSMUSG00000024800	Rpp30	1	1	1	3	10	13	15	11
ENSMUSG00000034321	Exosc1	0	2	4	0	8	16	16	9
ENSMUSG00000025008	Tctn3	1	2	1	0	10	8	7	7
ENSMUSG00000020280	Pus10	8	17	11	13	100	78	92	121
ENSMUSG00000015341	Golga7	23	18	17	26	157	144	188	181
ENSMUSG00000034163	Zfc3h1	7	4	8	4	43	28	49	60
ENSMUSG00000032126	Hmbs	9	6	9	5	59	57	54	53
ENSMUSG00000035227	Spcs2	8	2	5	5	37	35	44	37
ENSMUSG00000028848	Gpn2	3	3	8	6	38	31	34	50
ENSMUSG00000001062	Vps9d1	6	3	5	9	46	40	38	49
ENSMUSG00000024740	Ddb1	2	5	4	4	19	36	30	27
ENSMUSG00000036372	Tmem258	8	9	18	19	107	102	105	89
ENSMUSG00000042055	Wdr11	13	15	12	12	85	101	109	93
ENSMUSG00000022538	Lsg1	2	3	4	5	32	18	24	26
ENSMUSG00000032754	Slc8b1	30	24	23	14	237	155	126	131
ENSMUSG00000038342	Mlxip	3	3	3	6	29	14	30	32
ENSMUSG00000038759	Nup205	2	1	2	0	9	13	5	8
ENSMUSG00000003068	Stk11	2	2	6	5	28	24	25	27
ENSMUSG00000027309	Dnaaf9	4	1	5	0	25	11	19	13
ENSMUSG00000036985	Zdhhc9	7	13	9	11	78	61	72	59
ENSMUSG00000032599	Ip6k2	59	38	22	66	343	317	359	223
ENSMUSG00000025137	Pcyt2	634	621	733	664	5062	4167	4512	4004
ENSMUSG00000024410	Rmc1	3	4	3	2	17	14	22	27
ENSMUSG00000001674	Ddx18	3	4	9	8	43	36	37	43
ENSMUSG00000000441	Raf1	56	74	67	54	500	355	469	331
ENSMUSG00000024785	Rcl1	99	101	50	82	580	466	588	552
ENSMUSG00000034480	Diaph2	4	6	3	7	29	30	36	36
ENSMUSG00000030091	Nup210	1	0	1	0	3	3	6	1
ENSMUSG00000078786	Actmap	0	0	1	2	3	5	2	9
ENSMUSG00000001289	Pfdn5	8	12	12	8	69	62	59	61
ENSMUSG00000044730	Airim	8	10	12	17	90	53	78	72
ENSMUSG00000029427	Zcchc8	1	1	2	1	5	3	10	13
ENSMUSG00000001604	Tcea3	50	38	25	35	288	257	197	172
ENSMUSG00000029038	Ssu72	49	69	68	64	380	352	412	389
ENSMUSG00000053436	Mapk14	174	127	167	213	945	822	1166	1098
ENSMUSG00000064294	Aox3	78	47	73	66	427	307	435	392
ENSMUSG00000040811	Eml2	2	5	4	4	23	17	27	19
ENSMUSG00000039233	Tbce	12	9	16	11	84	66	74	45
ENSMUSG00000043987	Cep164	1	1	1	2	6	9	8	5
ENSMUSG00000042532	Golga7b	0	1	1	0	5	2	2	2
ENSMUSG00000034075	Zdhhc5	3	0	1	0	6	5	2	9
ENSMUSG00000027411	Vps16	5	2	5	2	14	17	21	23
ENSMUSG00000014850	Msh3	4	2	7	2	18	14	20	28
ENSMUSG00000027387	Zc3h8	4	3	7	5	28	29	21	23
ENSMUSG00000006010	Odr4	7	8	11	16	61	43	62	54
ENSMUSG00000060579	Fhit	7	7	3	5	35	24	32	24

ENSMUSG00000029427	Zcchc8	7	4	16	2	51	18	31	51
ENSMUSG00000028647	Mycbp	5	3	6	5	18	28	24	28
ENSMUSG00000026048	Ercc5	0	2	1	4	9	10	10	6
ENSMUSG00000033124	Atg9a	3	0	0	1	6	4	3	7
ENSMUSG00000055128	Cgrrf1	7	1	5	3	24	12	21	20
ENSMUSG00000090553	Snrpe	2	1	3	2	13	9	8	7
ENSMUSG00000029363	Rfc5	1	3	2	1	11	11	6	4
ENSMUSG00000019143	Hars2	11	14	13	20	57	51	77	77
ENSMUSG00000039917	Rhbdd2	7	7	15	13	48	38	40	61
ENSMUSG00000004264	Phb2	8	10	12	11	33	46	42	52
ENSMUSG00000035845	Alg12	1	3	3	3	13	7	12	10
ENSMUSG00000051329	Nup160	2	0	0	3	4	2	8	7
ENSMUSG00000032946	Rasgrp2	3	6	2	5	26	15	12	14
ENSMUSG00000005982	Naa60	14	20	26	17	74	73	74	91
ENSMUSG00000057469	E2f6	39	20	22	19	90	84	109	119
ENSMUSG00000027649	Ctnnbl1	7	6	7	8	21	31	32	26
ENSMUSG00000037553	Zdhhc18	1	4	5	3	8	11	13	19
ENSMUSG00000091625	Lsm5	0	3	2	2	6	7	7	7
ENSMUSG00000002221	Paxip1	7	8	9	10	21	33	43	33
ENSMUSG00000020903	Stx8	4	5	4	8	29	16	17	18
ENSMUSG00000031774	Psme3ip1	44	44	38	45	160	131	173	173
ENSMUSG00000028676	Srsf10	17	24	22	17	74	55	80	88
ENSMUSG00000033808	Tmem87a	10	17	15	8	50	38	54	43
ENSMUSG00000028173	Wls	4	2	1	1	4	6	10	9
ENSMUSG00000047921	Trappc9	10	5	6	2	16	27	28	12
ENSMUSG00000057335	Cep170	2	2	2	1	8	4	4	9
ENSMUSG00000036323	Srp72	17	9	8	13	43	29	53	39
ENSMUSG00000027936	Crtc2	19	25	22	23	67	76	93	73
ENSMUSG00000028760	Eif4g3	9	11	6	5	24	22	22	33
ENSMUSG00000010175	Prox1	25	19	57	58	125	88	116	177
ENSMUSG00000060279	Ap2a1	42	44	43	42	114	122	173	135
ENSMUSG00000002957	Ap2a2	3	6	3	3	7	13	18	9
ENSMUSG00000032127	Vps11	22	22	19	16	73	37	78	57
ENSMUSG00000031826	Usp10	2	4	2	2	8	8	9	6
ENSMUSG00000027778	Ift80	5	3	9	5	22	16	17	13
ENSMUSG00000059540	Tcea2	2	1	2	0	3	5	4	3
ENSMUSG00000070426	Rnf121	1	1	4	1	4	5	5	7
ENSMUSG00000053565	Eif3k	4	5	1	1	10	4	11	8
ENSMUSG00000002221	Paxip1	26	37	43	37	109	106	109	103
ENSMUSG00000033632	AW554918	9	6	8	9	23	18	28	23
ENSMUSG00000024456	Diaph1	23	23	19	18	52	52	79	55
ENSMUSG00000000355	Mcts1	152	139	189	198	496	469	489	451
ENSMUSG00000039018	Mtg1	7	6	1	4	12	10	11	16
ENSMUSG00000027900	Dram2	5	5	4	7	19	11	13	13
ENSMUSG00000020949	Fkbp3	42	45	68	53	150	118	147	136
ENSMUSG00000028233	Tgs1	5	5	7	8	11	21	16	17
ENSMUSG00000018474	Chd3	1	2	1	1	4	2	3	4
ENSMUSG00000080268	Brms1	6	8	10	5	25	24	8	15
ENSMUSG00000025377	Tepsin	3	13	7	15	31	17	23	21
ENSMUSG00000020955	Ap4s1	25	24	28	34	64	57	76	71
ENSMUSG00000039199	Zdhhc1	15	15	19	14	43	32	27	47
ENSMUSG00000035798	Zdhhc17	4	7	6	4	12	14	14	9
ENSMUSG00000027222	Pex16	11	6	12	9	18	22	23	24
ENSMUSG00000038542	Pcid2	12	19	14	15	36	24	37	38
ENSMUSG00000022761	Lztr1	2	9	10	11	25	12	20	15
ENSMUSG00000015165	Hnrnpl	11	16	9	6	25	18	33	18
ENSMUSG00000002728	Naa20	9	11	13	10	27	23	22	23
ENSMUSG00000022707	Gbe1	24	29	26	24	65	50	62	47

ENSMUSG00000030718	Ppme1	10	15	16	20	32	30	40	30
ENSMUSG00000028969	Cdk5	22	15	25	25	49	48	47	43
ENSMUSG00000039130	Zc3hc1	9	8	8	7	13	18	19	17
ENSMUSG00000049044	Rapgef4	259	246	410	540	723	527	748	1041
ENSMUSG00000026755	Arpc5l	37	37	36	30	75	50	71	91
ENSMUSG00000036875	Dna2	13	16	16	13	25	29	26	31
ENSMUSG00000005103	Wdr1	22	25	29	19	51	36	51	43
ENSMUSG00000034175	Rhbdd3	29	20	20	19	56	38	36	33
ENSMUSG00000058240	Cryzl1	17	16	20	14	30	34	31	21
ENSMUSG00000020925	Ccdc43	19	16	13	23	35	30	24	33
ENSMUSG00000068039	Tcp1	64	87	109	78	113	127	176	152
ENSMUSG00000037475	Thoc2	5	4	2	3	6	5	7	5
ENSMUSG00000071078	Nr2c2ap	32	30	50	42	42	68	80	61
ENSMUSG00000037110	Ralgapa2	42	43	39	35	59	59	53	78
ENSMUSG00000096764	Gm21985	447	481	481	412	522	537	626	597
ENSMUSG00000053329	Gatd3a	192	207	170	173	148	154	144	170
ENSMUSG00000028563	Tm2d1	48	35	32	36	12	20	13	21
ENSMUSG00000031879	Ciao2b	58	62	57	43	16	18	14	22
ENSMUSG00000074476	Spc24	33	35	31	20	8	12	8	5
ENSMUSG00000029298	Gbp9	0	0	0	1	8	1	5	2
ENSMUSG00000096780	Tmem181b	1	0	0	0	3	2	3	0
ENSMUSG00000027552	E2f5	1	1	2	2	2	3	8	7
ENSMUSG00000022272	Myo10	0	0	0	0	1	3	2	0
ENSMUSG00000042851	Zc3h6	0	0	1	1	7	5	1	2
ENSMUSG00000022003	Slc25a30	4	6	13	21	34	28	147	91
ENSMUSG00000030323	Ift122	1	0	0	0	2	4	3	0
ENSMUSG00000029777	Gars	54	56	50	40	57	62	55	61
ENSMUSG00000021392	Nol8	0	0	0	0	0	1	3	4
ENSMUSG00000037341	Slc9a7	0	0	0	0	0	1	3	4
ENSMUSG00000038141	Tmem181a	1	1	1	1	1	2	5	4
ENSMUSG00000030323	Ift122	0	0	1	2	2	4	1	3
ENSMUSG00000039463	Slc9a8	0	1	1	0	7	2	1	9
ENSMUSG00000058626	Capn11	0	0	0	0	4	0	5	1
ENSMUSG00000071708	Sms	0	0	0	0	1	0	4	5
ENSMUSG00000002319	Ipo4	3	0	6	7	10	5	9	8
ENSMUSG00000001998	Ap4e1	0	0	0	0	0	1	4	2
ENSMUSG00000020143	Dock2	0	0	0	0	2	1	4	0
ENSMUSG00000086837	Gm16618	5	3	4	9	8	6	18	14
ENSMUSG00000031129	Slc9a9	6	6	4	6	5	8	11	9
ENSMUSG00000017765	Slc12a4	1	0	1	1	9	3	4	1
ENSMUSG00000001767	Crnkl1	0	1	2	1	2	4	5	1
ENSMUSG00000057181	5730455P1	0	0	0	0	0	1	1	3
ENSMUSG00000024914	Drap1	0	3	4	3	3	6	6	4
ENSMUSG00000002007	Srpk3	0	1	0	1	2	1	1	1
ENSMUSG00000071691	Gm960	1	1	0	1	2	2	1	1
ENSMUSG00000027490	E2f1	5	2	3	3	1	1	2	3
ENSMUSG00000042564	Fam227a	1	4	1	1	6	5	1	5
ENSMUSG00000078963	Hsbp1l1	8	0	3	0	14	6	11	3
ENSMUSG00000041992	Rapgef5	0	0	0	1	2	0	3	1
ENSMUSG00000024142	Mlst8	1	1	0	1	6	0	2	5
ENSMUSG00000024472	Dcp2	0	0	0	0	0	1	1	0
ENSMUSG00000039530	#N/A	0	0	0	0	0	1	0	1
ENSMUSG00000039748	Exo1	0	0	0	0	1	0	0	1
ENSMUSG00000071042	Rasgrp3	0	0	0	0	0	0	1	1
ENSMUSG00000079110	Capn3	0	0	0	0	1	0	1	0
ENSMUSG00000093880	Tmem181c	0	0	0	0	0	1	1	0
ENSMUSG00000006519	Cyba	0	0	0	0	0	2	2	0
ENSMUSG00000058070	Eml1	0	0	0	0	2	0	2	0

ENSMUSG00000071708	Sms	1	3	1	0	1	3	6	3
ENSMUSG00000037344	Slc12a9	0	1	1	0	0	4	4	1
ENSMUSG00000038384	Setd1b	0	3	2	2	5	1	4	4
ENSMUSG00000014353	Tmem87b	15	11	6	6	24	13	10	15
ENSMUSG00000037818	Abhd18	9	16	12	13	15	18	18	12
ENSMUSG00000074673	Ttll9	0	0	0	0	1	1	5	0
ENSMUSG00000012076	Brms1l	0	0	0	0	0	1	0	2
ENSMUSG00000018983	E2f2	0	0	0	0	0	1	2	0
ENSMUSG00000026207	Speg	0	0	0	0	0	1	2	0
ENSMUSG00000037475	Thoc2	0	0	0	0	0	2	0	1
ENSMUSG00000040473	Cfap69	0	0	0	0	0	0	2	4
ENSMUSG00000027130	Slc12a6	0	1	0	0	2	1	1	0
ENSMUSG00000053137	Mapk11	1	2	1	0	0	0	1	0
ENSMUSG00000053483	Usp21	17	18	24	22	22	22	23	25
ENSMUSG00000004667	Polr2e	1	4	2	7	6	4	9	5
ENSMUSG00000033809	Alg3	37	35	52	20	39	38	55	63
ENSMUSG00000022469	Rapgef3	0	0	0	0	0	1	3	0
ENSMUSG00000038122	Tbc1d32	0	0	0	0	0	0	3	1
ENSMUSG00000060166	Zdhhc8	0	1	0	0	1	1	1	0
ENSMUSG00000038860	Garnl3	1	0	0	1	3	1	2	0
ENSMUSG00000038593	Tctn1	2	5	0	3	4	5	3	4
ENSMUSG00000031832	Taf1c	1	1	1	0	0	3	5	1
ENSMUSG00000037341	Slc9a7	0	0	3	2	8	2	5	0
ENSMUSG00000062590	Armc9	0	1	1	0	2	2	11	0
ENSMUSG00000056598	Drc3	0	0	1	0	3	0	0	2
ENSMUSG00000024456	Diaph1	0	0	2	0	0	1	3	2
ENSMUSG00000038279	Nop2	0	2	0	3	1	1	4	5
ENSMUSG00000032010	Usp2	3	1	0	1	2	1	1	19
ENSMUSG00000010721	Lmbr1	0	0	0	0	1	0	0	0
ENSMUSG00000015968	Cacna1d	0	0	0	0	1	0	0	0
ENSMUSG00000020577	Tspan13	0	0	0	0	0	0	1	0
ENSMUSG00000020974	Pole2	0	0	0	0	0	1	0	0
ENSMUSG00000022021	Diaph3	0	0	0	0	0	0	0	1
ENSMUSG00000023033	Scn8a	0	0	0	0	0	0	0	1
ENSMUSG00000024388	Myo7b	0	0	0	0	1	0	0	0
ENSMUSG00000024500	Ppp2r2b	0	0	0	0	1	0	0	0
ENSMUSG00000031129	Slc9a9	0	0	0	0	0	0	1	0
ENSMUSG00000031142	Cacna1f	0	0	0	0	0	0	1	0
ENSMUSG00000040729	Cep126	0	0	0	0	1	0	0	0
ENSMUSG00000042564	Fam227a	0	0	0	0	0	0	1	0
ENSMUSG00000043051	Disc1	0	0	0	0	1	0	0	0
ENSMUSG00000050640	Tmem150c	0	0	0	0	1	0	0	0
ENSMUSG00000105504	Gbp5	0	0	0	0	0	1	0	0
ENSMUSG00000028524	Sgip1	0	0	0	0	0	0	0	2
ENSMUSG00000039943	Plcb4	0	0	0	0	0	0	2	0
ENSMUSG00000021256	Vash1	0	0	0	0	0	0	3	0
ENSMUSG00000022324	Matn2	0	1	0	0	0	1	3	0
ENSMUSG00000010066	Cacna2d2	0	1	0	0	0	0	0	0
ENSMUSG00000038242	Aox4	1	0	0	0	0	0	0	0
ENSMUSG00000061298	Agbl4	1	0	0	0	0	0	0	0
ENSMUSG00000097065	#N/A	1	0	0	0	0	0	0	0
ENSMUSG00000104082	Gm7115	0	0	0	1	0	0	0	0
ENSMUSG00000115137	Gm34794	1	0	0	0	0	0	0	0
ENSMUSG00000027347	Rasgrp1	3	0	0	0	0	0	0	0
ENSMUSG00000038563	Efl1	0	1	0	0	0	1	2	0
ENSMUSG00000031434	Morc4	5	2	0	3	6	5	3	1
ENSMUSG00000054408	Spcs3	27	36	16	19	37	23	37	21
ENSMUSG00000022100	Xpo7	2	0	1	0	2	2	0	1

ENSMUSG00000021256	Vash1	1	0	0	0	1	0	0	1
ENSMUSG00000099139	1110028F1	0	0	1	0	0	0	1	1
ENSMUSG00000041406	Firrm	0	0	1	1	1	1	0	1
ENSMUSG00000056598	Drc3	0	1	1	0	1	1	0	1
ENSMUSG00000001173	Ocrl	0	1	1	0	0	1	0	0
ENSMUSG00000084896	Gm11632	23	16	14	15	16	14	20	11
ENSMUSG00000046456	Tmem150b	0	0	2	0	0	0	3	1
ENSMUSG00000032549	Rab6b	8	19	18	11	13	12	14	11
ENSMUSG00000042447	Mios	1	1	0	1	2	1	0	1
ENSMUSG00000041685	Fcho2	18	12	16	16	11	14	21	22
ENSMUSG00000073094	Smim9	0	0	1	2	1	0	0	4
ENSMUSG00000074476	Spc24	7	10	18	7	17	12	8	11
ENSMUSG00000042320	Prox2	5	7	7	11	10	7	6	10
ENSMUSG00000009092	Derl3	0	1	0	0	2	0	0	0
ENSMUSG00000031951	Tmem231	0	0	1	0	0	0	2	0
ENSMUSG00000022610	Mapk12	1	0	1	0	2	0	1	0
ENSMUSG00000024122	Pdpk1	0	0	0	2	0	1	1	1
ENSMUSG00000070000	Fcho1	2	0	0	1	1	0	1	0
ENSMUSG00000038020	Rapgefl1	0	0	0	2	0	1	0	0
ENSMUSG00000027939	Nup210l	3	4	5	1	2	8	2	3
ENSMUSG00000020057	Dram1	2	2	3	1	3	0	3	1
ENSMUSG00000063458	Lrmda	1	1	3	0	2	0	2	0
ENSMUSG00000057594	Arl16	6	6	9	5	8	7	8	4
ENSMUSG00000045679	Slc66a3	5	2	0	0	7	0	0	1
ENSMUSG00000040061	Plcb2	0	0	0	1	1	0	0	0
ENSMUSG00000018378	Cuedc1	1	1	0	1	0	0	3	0
ENSMUSG00000075702	Selenom	1	0	1	1	1	1	1	0
ENSMUSG00000001027	Scn4a	0	0	0	0	0	0	0	0
ENSMUSG00000003309	Ap1m2	0	0	0	0	0	0	0	0
ENSMUSG00000004110	Cacna1e	0	0	0	0	0	0	0	0
ENSMUSG00000004110	Cacna1e	0	0	0	0	0	0	0	0
ENSMUSG00000004113	Cacna1b	0	0	0	0	0	0	0	0
ENSMUSG00000004113	Cacna1b	0	0	0	0	0	0	0	0
ENSMUSG00000004864	Mapk13	0	0	0	0	0	0	0	0
ENSMUSG00000005045	Chd5	0	0	0	0	0	0	0	0
ENSMUSG00000005883	Spo11	0	0	0	0	0	0	0	0
ENSMUSG00000007034	Slc44a4	0	0	0	0	0	0	0	0
ENSMUSG00000009596	Taf7l	0	0	0	0	0	0	0	0
ENSMUSG00000016995	Matn4	0	0	0	0	0	0	0	0
ENSMUSG00000017740	Slc12a5	0	0	0	0	0	0	0	0
ENSMUSG00000017740	Slc12a5	0	0	0	0	0	0	0	0
ENSMUSG00000018830	Myh11	0	0	0	0	0	0	0	0
ENSMUSG00000019518	Ap4m1	0	0	0	0	0	0	0	0
ENSMUSG00000020583	Matn3	0	0	0	0	0	0	0	0
ENSMUSG00000020866	Cacna1g	0	0	0	0	0	0	0	0
ENSMUSG00000020937	Plcd3	0	0	0	0	0	0	0	0
ENSMUSG00000021991	Cacna2d3	0	0	0	0	0	0	0	0
ENSMUSG00000022021	Diaph3	0	0	0	0	0	0	0	0
ENSMUSG00000022186	Oxct1	0	0	0	0	0	0	0	0
ENSMUSG00000022249	Ttc23l	0	0	0	0	0	0	0	0
ENSMUSG00000022288	4930447A1	0	0	0	0	0	0	0	0
ENSMUSG00000022416	Cacna1i	0	0	0	0	0	0	0	0
ENSMUSG00000022753	Tmem30c	0	0	0	0	0	0	0	0
ENSMUSG00000022829	Stxbp5l	0	0	0	0	0	0	0	0
ENSMUSG00000023999	Kif6	0	0	0	0	0	0	0	0
ENSMUSG00000024112	Cacna1h	0	0	0	0	0	0	0	0
ENSMUSG00000024210	Ip6k3	0	0	0	0	0	0	0	0
ENSMUSG00000024786	Majin	0	0	0	0	0	0	0	0

ENSMUSG00000026407	Cacna1s	0	0	0	0	0	0	0	0
ENSMUSG00000026407	Cacna1s	0	0	0	0	0	0	0	0
ENSMUSG00000027130	Slc12a6	0	0	0	0	0	0	0	0
ENSMUSG00000027867	Spag17	0	0	0	0	0	0	0	0
ENSMUSG00000028360	Slc44a5	0	0	0	0	0	0	0	0
ENSMUSG00000029055	Plch2	0	0	0	0	0	0	0	0
ENSMUSG00000029120	Ppp2r2c	0	0	0	0	0	0	0	0
ENSMUSG00000029712	Actl6b	0	0	0	0	0	0	0	0
ENSMUSG00000030230	Plcz1	0	0	0	0	0	0	0	0
ENSMUSG00000030589	Rasgrp4	0	0	0	0	0	0	0	0
ENSMUSG00000031129	Slc9a9	0	0	0	0	0	0	0	0
ENSMUSG00000031181	Ctag2	0	0	0	0	0	0	0	0
ENSMUSG00000031981	Capn9	0	0	0	0	0	0	0	0
ENSMUSG00000032511	Scn5a	0	0	0	0	0	0	0	0
ENSMUSG00000032586	Traip	0	0	0	0	0	0	0	0
ENSMUSG00000034063	4930590J0	0	0	0	0	0	0	0	0
ENSMUSG00000034115	Scn11a	0	0	0	0	0	0	0	0
ENSMUSG00000034533	Scn10a	0	0	0	0	0	0	0	0
ENSMUSG00000034656	Cacna1a	0	0	0	0	0	0	0	0
ENSMUSG00000034656	Cacna1a	0	0	0	0	0	0	0	0
ENSMUSG00000034810	Scn7a	0	0	0	0	0	0	0	0
ENSMUSG00000036834	Plch1	0	0	0	0	0	0	0	0
ENSMUSG00000037341	Slc9a7	0	0	0	0	0	0	0	0
ENSMUSG00000037568	Vash2	0	0	0	0	0	0	0	0
ENSMUSG00000037568	Vash2	0	0	0	0	0	0	0	0
ENSMUSG00000038242	Aox4	0	0	0	0	0	0	0	0
ENSMUSG00000038295	Atg9b	0	0	0	0	0	0	0	0
ENSMUSG00000038498	Catsper1	0	0	0	0	0	0	0	0
ENSMUSG00000038599	Capn8	0	0	0	0	0	0	0	0
ENSMUSG00000039716	Dock3	0	0	0	0	0	0	0	0
ENSMUSG00000039959	Hip1	0	0	0	0	0	0	0	0
ENSMUSG00000040118	Cacna2d1	0	0	0	0	0	0	0	0
ENSMUSG00000040533	Matn1	0	0	0	0	0	0	0	0
ENSMUSG00000040594	Ranbp17	0	0	0	0	0	0	0	0
ENSMUSG00000040728	Esrp1	0	0	0	0	0	0	0	0
ENSMUSG00000041460	Cacna2d4	0	0	0	0	0	0	0	0
ENSMUSG00000042269	Cibar2	0	0	0	0	0	0	0	0
ENSMUSG00000043410	Hfm1	0	0	0	0	0	0	0	0
ENSMUSG00000043705	Capn13	0	0	0	0	0	0	0	0
ENSMUSG00000046709	Mapk10	0	0	0	0	0	0	0	0
ENSMUSG00000046991	Wdr27	0	0	0	0	0	0	0	0
ENSMUSG00000049571	Cfap46	0	0	0	0	0	0	0	0
ENSMUSG00000051331	Cacna1c	0	0	0	0	0	0	0	0
ENSMUSG00000051331	Cacna1c	0	0	0	0	0	0	0	0
ENSMUSG00000053153	Spag16	0	0	0	0	0	0	0	0
ENSMUSG00000056215	Lrguk	0	0	0	0	0	0	0	0
ENSMUSG00000057182	Scn3a	0	0	0	0	0	0	0	0
ENSMUSG00000060212	Pcnx2	0	0	0	0	0	0	0	0
ENSMUSG00000060268	Armh1	0	0	0	0	0	0	0	0
ENSMUSG00000061462	Obscn	0	0	0	0	0	0	0	0
ENSMUSG00000064329	Scn1a	0	0	0	0	0	0	0	0
ENSMUSG00000068117	Mei1	0	0	0	0	0	0	0	0
ENSMUSG00000069189	Zdhhc11	0	0	0	0	0	0	0	0
ENSMUSG00000072295	C2cd6	0	0	0	0	0	0	0	0
ENSMUSG00000075316	Scn9a	0	0	0	0	0	0	0	0
ENSMUSG00000075318	Scn2a	0	0	0	0	0	0	0	0
ENSMUSG00000078490	Cfap74	0	0	0	0	0	0	0	0
ENSMUSG00000078998	Bpifa6	0	0	0	0	0	0	0	0

ENSMUSG00000079532	Ctag2l2	0	0	0	0	0	0	0	0
ENSMUSG00000079536	Ctag2l1	0	0	0	0	0	0	0	0
ENSMUSG00000079554	Aox2	0	0	0	0	0	0	0	0
ENSMUSG00000082590	Gm14943	0	0	0	0	0	0	0	0
ENSMUSG00000083981	Gm15363	0	0	0	0	0	0	0	0
ENSMUSG00000085330	Gm13417	0	0	0	0	0	0	0	0
ENSMUSG00000086487	Gm11638	0	0	0	0	0	0	0	0
ENSMUSG00000086631	Gm12784	0	0	0	0	0	0	0	0
ENSMUSG00000087341	0610040F(	0	0	0	0	0	0	0	0
ENSMUSG00000087374	Gm15457	0	0	0	0	0	0	0	0
ENSMUSG00000090817	Hsd3b9	0	0	0	0	0	0	0	0
ENSMUSG00000095388	Hsd3b8	0	0	0	0	0	0	0	0
ENSMUSG00000099419	1700001D(	0	0	0	0	0	0	0	0
ENSMUSG00000102123	Gm4319	0	0	0	0	0	0	0	0
ENSMUSG00000105317	Gbp2-ps	0	0	0	0	0	0	0	0
ENSMUSG00000107153	Gm38404	0	0	0	0	0	0	0	0
ENSMUSG00000110717	1700047G(	0	0	0	0	0	0	0	0
ENSMUSG00000111550	Gm40623	0	0	0	0	0	0	0	0
ENSMUSG00000114958	Gm48612	0	0	0	0	0	0	0	0
ENSMUSG00000115221	Gm49121	0	0	0	0	0	0	0	0
ENSMUSG00000040594	Ranbp17	0	0	0	0	1	1	1	1

Table S3. Primer sequences for qPCR

Primer	Forward（5'-3'）	Reverse（5'-3'）
mSREBP1c	ATCGGCGCGGAAGCTGTCGG	GGGAAGTCACTGTCTTGGTTG
mSREBP2	TCAGCACCGCTCCGCAGACGAG	TACCGTCTGCACCTGCTGCTGG
mChREBP	GGGACAAGATCCGGCTGAA	GCTCTTCCTCCGTTGCACAT
mScd1	GCTGGAGTACGTCTGGAGGAA	TCCCGAAGAGGCAGGTGTAG
mFasn	GGTTACACTGTGCTAGGTGTTG	TCCAGGCGCATGAGGCTCAGC
mDgat2	GCGCTACTTCCGAGACTACTT	GGGCCTTATGCCAGGAAACT
mFsp27	TCGACCTGTACAAGCTGAACCCT	AGGTGCCAAGCAGCATGTGACC
mZrsr1	CGGTGGAAGGTTGCAATTTG	CACCGCTACGGCGGACGCTACGGCG
mEhhadh	CAGATGAAGCACTCAAGCTTG	ACCTTGGCAATGGCTTCTGCA
mHmgcs2	GACATCAACTCCCTGTGCCTG	GATGTCAGTGTTGCCTGAATC
mCcl2	AGGTCCCTGTCATGCTTCTG	TCTGGACCCATTCCTTCTTG
mCcl5	TGCCCACGTCAAGGAGTATTT	TTCTCTGGGTTGGCACACACT
mIL6	AGTTGCCTTCTTGGGACTGA	TCCACGATTTCCCAGAGAAC
mIL1β	GAAATGCCACCTTTTGACAGTG	TGGATGCTCTCATCAGGACAG
mTNFα	CAGGCGGTGCCTATGTCTC	CGATCACCCCGAAGTTCAGTAG
mPparα	GCAGTGCCCTGAACATCGA	CGCCGAAAGAAGCCCTTAC
mPparγ	GAAAGACAACGGACAAATCACC	GGGGGTGATATGTTTGAACTTG
mLxra	AGGCTCAAGCCACTTCGGTGTC	AGAAGGAGCGCCTGTTACACTG
mCol1a1	AAGAGGCGAGAGAGGTTTCC	AGAACCATCAGCACCTTTGG
mCol1a2	GTAACTTCGTGCCTAGCAACA	CCTTTGTCAGAATACTGAGCAGC
mCol3a1	CTGTAACATGGAAACTGGGGAAA	CCATAGCTGAACTGAAAACCACC

mCol5a2	ACAGGTGAAGTGGGATTCTCA	CCATAGCACCCATTGGACCA
mCol5a3	CGGGGTACTCCTGGTCCTAC	GCATCCCTACTTCCCCCTTG
mCol6a1	CTGCTGCTACAAGCCTGCT	CCCCATAAGGTTTCAGCCTCA
mCol6a2	AAGGCCCCATTGGATTCCC	CTCCCTTCCGACCATCCGAT
mCol6a3	GCTGCGGAATCACTTTGTGC	CACCTTGACACCTTTCTGGGT
mFL-Insig1	CTAGTGCTCTTCTCATTTGGCG	AGGGATACAGTAAACCGACAACA
mFL-Insig2	AGAGTGGTCCAGTGTCATGC	CAGCCAGTGTGAGGGAAAAC
mInsig1-U12	TTTCTGTGCTACGTCCAGAGT	GGGTAGGTACCACCATCATGT
mInsig2-U12	AAGGACCTTGAGGGAGTTGC	TTGCAACGAAGCCATTTCCC
mIDH1	AGTCGCCCAAGGTTATGGC	TGCTTCTACCGTCTTACCATCT
mIDH2	ATTTTGTGGTAGATCGAGCTGG	CCTCCGGCAGGGAAGTTATAC
mAp0	GAAACTGCTGCCTCACATCCG	GCTGGCACAGTGACCTCACACG
18s	AGCCCCTGCCCTTTGTACACA	CGATCCGAGGGCCTCACTA
hZRSR2	AAACGAAAGAAACGTCGGCA	AGATCACACCACTGCACTCCAG
hU12-Insig1	GACTCAGTGATGGCAGGGAG	CCACAAGGGTTAAAAACATGGGT
hU12-Insig2	AGTGGTCAGGAGGGCTTAAA	CACAGTTTTGAGGTTCCTGGG
