# Supplementary material for: Disrupted minor intron splicing activates reductive carboxylation-mediated lipogenesis to drive metabolic dysfunction–associated steatotic liver disease progression
Source: J Clin Invest. 2025 Mar 18;135(10):e186478. doi: 10.1172/JCI186478 (PMC12077890; doi:10.1172/JCI186478)
Supplement: Supplemental data [file jci-135-186478-s231.pdf]

Supplemental information

Materials and Methods

Genotyping

Whole genomic DNA was derived from the tail or cell after lysing with 50 mM NaOH at 98°C for 30 min and neutralized with 1M Tris-HCl (pH 8.0). This crude DNA was then used for PCR using 2X Rapid Taq Master Mix (Vazyme, P222-01), according to the manufacturer's instructions and following PCR conditions: one cycle of 95°C for 3 min, 35 cycles of 95°C for 15 s, 60°C for 15 s, 72°C for 60 s, and one cycle of 72°C for 5 min. Primers for Zrsr1 KO; forward, cacggcagaccgcgattcctg; reverse, ggaagttgcagtgctttcctt; For Zrsr2 KO; forward, gagtgggcgaccgaggacct; reverse, ggagcaattccacatccagagc. The PCR products were subsequently separated using 1 % agarose gel electrophoresis for 30 min.

Cell lines

Primary hepatocytes were isolated from C57BL/6J mice via collagenase type II as previously described (1). Hepatocytes were maintained in DMEM containing 10% FBS and 1% penicillin–streptomycin at 37°C and 5% CO_2. Adenovirus infection was performed on the same day as isolation. After 24 h, the cells were treated with vehicle (DMSO), T0901317 (5 μM) or SR9238 (10 μM) (Sigma, SM1510) for 24 h. AML12 cells were purchased from the American Type Culture Collection (ATCC) and cultured in DMEM/F-12 (1:1) supplemented with 10% FBS, 1% penicillin, streptomycin, dexamethasone and ITS. HEK293T cells were

cultured in DMEM supplemented with 10% BCS and 1% penicillin–streptomycin. Immortalized murine hepatic stellate cells (HSCs) were obtained from Dr. Xuelian Xiong at Zhongshan Hospital, Fudan University in Shanghai, and cultured in DMEM supplemented with 10% FBS and 1% penicillin–streptomycin. The cell culture experiments were performed in triplicate and repeated at least three times.

Liver histology

Tissues were dissected and fixed in 10% formalin overnight at 4°C and subjected to paraffin embedding and H&E staining. Liver slices were fixed in O.C.T. (G6059, Servicebio) and then stained with Oil Red O. Sirius red staining was performed as previously described (2). Paraffin sections were subjected to ammonia staining with Nessler´s reagent (Sigma, 1090280100) as previously described (3).

Metabolic analyses

For the glucose tolerance test (GTT), the mice were fasted overnight (16 h) and injected intraperitoneally (IP) with a glucose solution at a dose of 1.0 g/kg body weight. For the insulin tolerance test (ITT), the mice were fasted for 4 h and IP injected with insulin at a dose of 1 U/kg body weight. Blood glucose concentrations were measured before and at 20, 45, 90 and 120 min after glucose or insulin injection. Liver triglycerides and cholesterol were extracted and measured as previously described (4). Plasma insulin concentrations were

measured via an ELISA kit (CrystalChem, 90096). Plasma triglycerides, NEFAs, β-hydroxybutyrate, alanine transaminase (ALT), aspartate transaminase (AST) and cholesterol were measured via commercial kits. The hydroxyproline level in the liver was measured via metabolomics. Liver and cellular ammonia contents were measured via an ammonia assay kit (Sigma, MAK310). Liver α-ketoglutarate (α-KG) was measured via an α-ketoglutarate assay kit (Cayman, 701350). Widely targeted metabolomics of liver tissues was performed by MetWare Biotechnology. Metabolites of liver tissues were detected based on the AB Sciex QTRAP 6500 LC–MS/MS platform. Enrichment analysis was performed via Metaboanalyst (www.metaboanalyst.ca).

Gene expression analyses

Mouse and human livers were extracted and immediately frozen in liquid nitrogen. Total RNA was extracted from tissues or hepatocytes via the TRIzol method following the manufacturer's instructions. For RT–qPCR, 2 μg of RNA was reverse transcribed via HiScript II Q RT SuperMix (Vazyme, R222-01), followed by qPCR via SYBR Green. Relative mRNA expression was normalized to the expression of AP0 or 18S. The primers used for gene expression are listed in Table S3.

Immunoblotting analysis

Mouse liver tissue was extracted and immediately frozen in liquid nitrogen. Tissue lysates were prepared by homogenization in buffer containing 50 mM Tris

(pH=7.6), 130 mM NaCl, 5 mM NaF, 25 mM β-glycerophosphoate, 1 mM sodium orthovanadate, 10% glycerol, 1% Triton X-100, 1 mM DTT, 1 mM PMSF and the protease inhibitor cocktail. Total cell lysates were prepared in lysis buffer containing 50 mM Tris-HCl (pH=7.8), 137 mM NaCl, 10 mM NaF, 1 mM EDTA, 1% Triton X-100, 10% glycerol, and a protease inhibitor cocktail (Bimake, B14002) after three freeze–thaw cycles. The ZRSR1 customized antibody was developed by PTM BIO. Briefly, the antibody was produced from rabbits immunized with a HIS-tagged protein corresponding to the C-terminal region (amino acids 200-420) of the murine ZRSR1 protein. The antibody serum was subsequently purified using Protein A and antigen affinity chromatography. The other commercial antibodies used were ZRSR2 (PA5-41797) from Invitrogen, SREBP-1 (sc-13551) and INSIG1 (sc-390504) from Santa Cruz Biotechnology. HSP90 (13171-1-AP), FLAG (20543-1-AP), IDH1 (12322-1-AP), NF-κB p65 (65107451-1-AP), and JNK (66210-1-Ig) were obtained from Proteintech. Phospho-AMPK (2535S), AMPK (2532), phospho-NF-κB P65 (3033), phospho-JNK (4668) and cleaved caspase3 (9661) were obtained from Cell Signaling Technology.

RNA-Seq Data Analysis

Total RNA was denatured and enriched by oligo (dT)-attached magnetic beads to obtain mRNA. mRNAs were fragmented for cDNA synthesis. cDNAs were then subjected to End Repair, Add A and Adaptor Ligation, and PCR amplification. The RNA library was qualified and then sequenced on DNBseq-T7 using High-

throughput Sequencing Kit (PE 150). The fastq files were subjected to quality control via FastQC followed by alignment against the mouse reference genome (mm38) via the aligner HISAT2 (5). HTSeq program was employed to count the number of reads mapped to the reference obtained from NCBI for coding genes and from the IAOD website for major/minor intron regions (6, 7). The fold change of the counts of major/minor intron reads was calculated to evaluate the level of minor and major intron retention in MASH and ZLKO mice. The percentage of major/minor retained introns is calculated by dividing the number of major/minor retained introns by all major/minor introns in major/minor intron-containing genes. In total, 657 minor introns found within 606 genes, and 211,005 major introns found within 32,396 genes, were taken into consideration for the calculation. DESeq2 was used for differential expression analysis (8). Transcripts exhibiting significant differences of more than 1.4-fold in expression in the ZLKO group compared with the CTR group were selected for further analysis. Gene enrichment analysis was performed via DAVID (https://david.ncifcrf.gov/). The StringTie method was applied to reconstitute the Insig1 and Insig2 transcripts (9).

In vitro lipogenesis assay

AML12 cells were incubated in Krebs Ringer buffer supplemented with 0.1% fatty acid-free BSA. Each well was incubated with 0.2 μCi/mL [U-^{14}C]acetate (PerkinElmer, NEC085H001MC) or [U-^{14}C]glucose (PerkinElmer, NEC042X050UC) for 2 h in the presence of vehicle or T0901317 alone or in combination with T0901317 and GSK864 (MCE, HY-19540). The cells were

lysed, and lipids were extracted with hexane-isopropanol solution (hexane: isopropanol (v:v) =3:2), evaporated and resuspended in toluene. Radioactivity was measured in scintillation fluid.

Metabolic flux analysis

AML12 cells were seeded in 6-well plates with unlabeled DMEM/F12 medium, and the media were removed when the cells had grown to 60% confluence. The cells were subsequently washed twice with PBS. The medium was then replaced with glutamine-free DMEM/F12 medium supplemented with 2.5 mM [U-^{13}C]glutamine (Sigma, 605166), 10% dialyzed FBS, 1% penicillin streptomycin, dexamethasone and ITS. After 24 h of labeling, the cells were treated with vehicle, T0901317 alone or T0901317 combined with GSK864 for 24 h. Afterward, the cells were washed with PBS. For glutamine-to-fatty acid flux analysis, the cells were collected in a 1.5-mL centrifuge tube. Fatty acids were extracted with 1 mL of 90% MeOH and 0.3 M KOH. After vortexing for 10 s, the solution was transferred to a 4 mL glass bottle and heated at 80°C for 1 h. Samples were collected by adding 100 μL of formic acid and 1 mL of hexane, vortexing and transferring to HPLC glass bottles, followed by evaporation to dryness in a vacuum concentrator. The dry extracts were reconstituted in 200 μL of ACN:MeOH (1:1, v/v) per million cells and stored at −20°C prior to LC–MS analysis. LC–MS analysis was performed via a Vanquish UHPLC system (Thermo Fisher Scientific) and an Acquity UPLC BEH C18 column (2.1×100 mm, 1.75 μm; Waters). For glutamine-to-citrate flux analysis, the cells were placed on

ice and extracted with 40 μl of MeOH:ACN:H_2O (2:2:1, v/v/v) mixture. The cells were scraped from the plate, transferred to a 1.5-mL centrifuge tube and incubated on ice for 10 minutes. The samples were vortexed for 30 seconds and centrifuged at 14000 rpm for 20 minutes at 4°C. The supernatants were transferred to a new 1.5-mL tube and centrifuged again at 14000 rpm for 20 minutes. The supernatants were ultimately transferred to HPLC glass bottles. LC separation was achieved via a Vanquish UHPLC system (Thermo Fisher Scientific) and a HILIC column (2.1×150 mm, 5 μm, HILICON). The raw data were converted to mzXML format via MSConver and processed via EI-MAVEN for peak detection, extraction, alignment and integration. Natural isotope abundance correction was applied via METLAB. The enrichment of lipogenic acetyl-CoA was calculated with FAMetA software (www.fameta.es) (10).

Co-culture of AML12 cells and HSCs

AML12 cells in glutamine-free DMEM/F12 medium supplemented with FBS, PS, dexamethasone and ITS were seeded in 12-well plates. At the same time, HSCs in glutamine-free DMEM supplemented with FBS and PS were seeded into 24 mm tissue culture transwell inserts (Corning, 3412) and incubated for 24 h. When the AML12 cells were 70% confluent, the cells were treated with vehicle or T0901317, and the HSCs containing inserts were transferred to AML12 cell-seeded plates. The cells were co-cultured for 24 h at 37°C, after which the HSCs were collected for analysis of gene expression. The experiments were performed in triplicate.

FIGURES

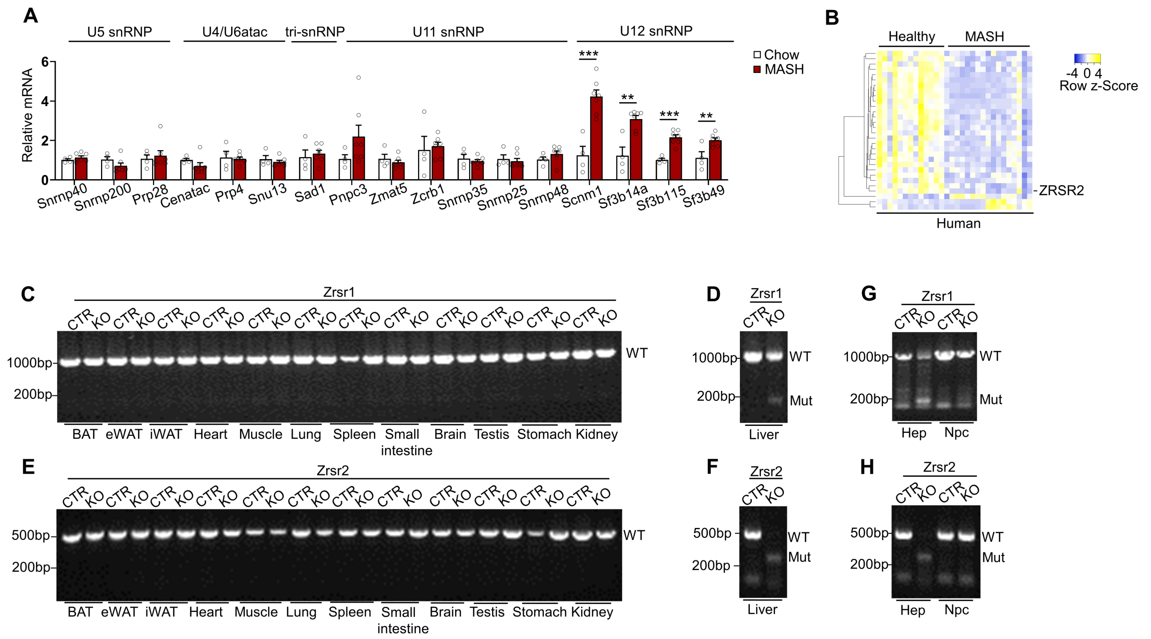

Figure S1. Hepatic expression of minor intron splicing factors and snRNAs under MASH condition and the genotyping of Zrsr1 and Zrsr2 double-deficient mice.

(**A**) QPCR analysis of minor intron splicing factors and snRNAs in the livers of mice fed chow-diet (*n*=4) or CDA-HFD (MASH diet, *n*=7) for 6 months. (**B**) Heatmap showing the gene expression of RNA binding proteins (RBPs), including ZRSR2, in the livers of healthy individuals and MASH patients (GSE126848). (**C**-**F**) *Zrsr1* (**C**-**D**) and *Zrsr2* (**E**-**F**) genotyping of BAT, eWAT, iWAT, heart, muscle, lung, spleen, small intestine, brain, testis, stomach, kidney (**C**, **E**) and liver (**D**, **F**) from AAV-control- (CTR) and AAV-sgRNA-injected Cas9-Tg mice (ZLKO). (**G**-**H**) *Zrsr1* (**G**) and *Zrsr2* (**H**) genotyping of isolated hepatocytes (Hep) and hepatic nonparenchymal cells (Npc) from the livers of CTR and ZLKO mice. The data are presented as the mean ± SEM. $^{**}p < 0.01$, $^{***}p < 0.001$ by two-tailed unpaired Student's t-test (A).

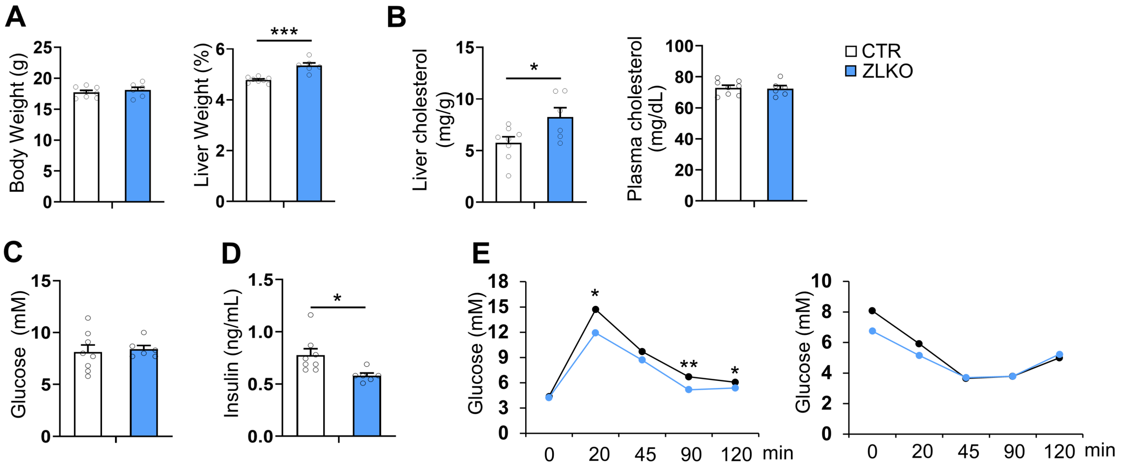

Figure S2. Metabolic phenotypes of Zrsr1 and Zrsr2 double-deficient mice after chow-diet feeding.

(**A**) Body weights (left) and percentage of liver weights (right) of CTR (*n*=8) and ZLKO (*n*=6) mice after chow diet feeding for 1 week. (**B**) Liver (left) and plasma (right) cholesterol contents in CTR and ZLKO mice on chow diet. (**C**-**D**) Plasma glucose (**C**) and insulin (**D**) levels in liver tissues from CTR and ZLKO mice. (**E**) GTT and ITT assay in the CTR (*n*=8) and ZLKO (*n*=6) mice after chow diet feeding. The data are presented as the mean ± SEM. *p<0.05, **p < 0.01, ***p < 0.001 by two-tailed unpaired Student's t-test (A-D), by two-way ANOVA with multiple comparisons (E).

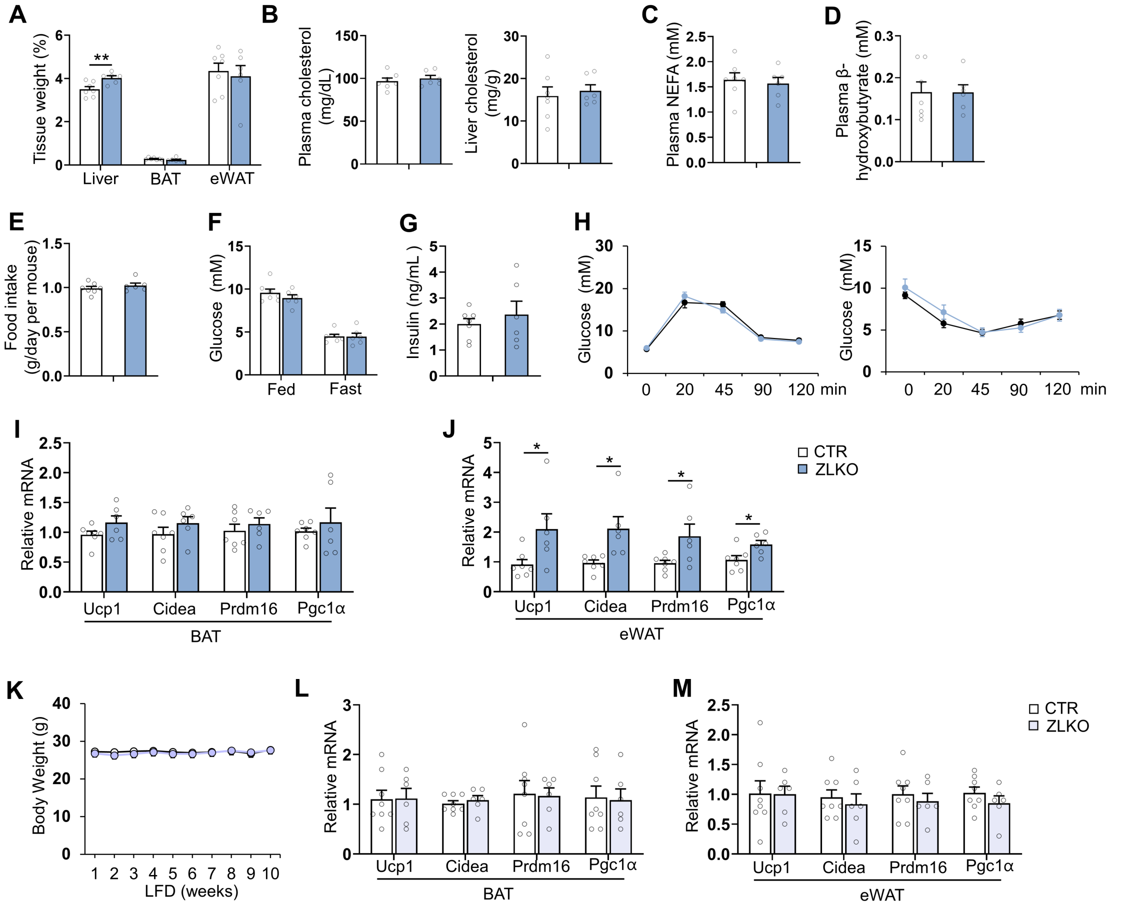

Figure S3. Metabolic and thermogenic phenotypes of Zrsr1 and Zrsr2 double-deficient mice in obese and lean conditions.

(**A**) Percentage of tissue weights of CTR (*n*=7) and ZLKO (*n*=6) mice fed high-fat diet (HFD) for 10 weeks. (**B**) Plasma (left) and liver (right) cholesterol contents in CTR and ZLKO mice after HFD feeding. (**C**-**D**) Plasma non-esterified fatty acid (NEFA, **C**) and β-hydroxybutyrate levels (**D**) in CTR and ZLKO mice after HFD feeding. (**E**) Food intake of CTR and ZLKO mice after HFD feeding. (**F**-**G**) Plasma glucose (**F**) and insulin (**G**) levels in the livers of CTR and ZLKO mice fed HFD. (**H**) GTT and ITT assays in the CTR and ZLKO mice after HFD feeding. (**I**-**J**) QPCR analysis of the expression of thermogenic genes in the BAT (**I**) and eWAT (**J**) of CTR and ZLKO mice after HFD feeding. (**K**) Body weights curve of CTR (*n*=8) and ZLKO (*n*=6) mice fed low-fat diet (LFD) for 10 weeks. (**L**-**M**) QPCR analysis of the expression of thermogenic genes in the BAT (**L**) and

eWAT (**M**) of CTR and ZLKO mice after LFD feeding. The data are presented as the mean ± SEM. *$p<0.05$, **$p < 0.01$ by two-tailed unpaired Student's t-test (A-G, I-J, and L-M), by two-way ANOVA with multiple comparisons (H and K).

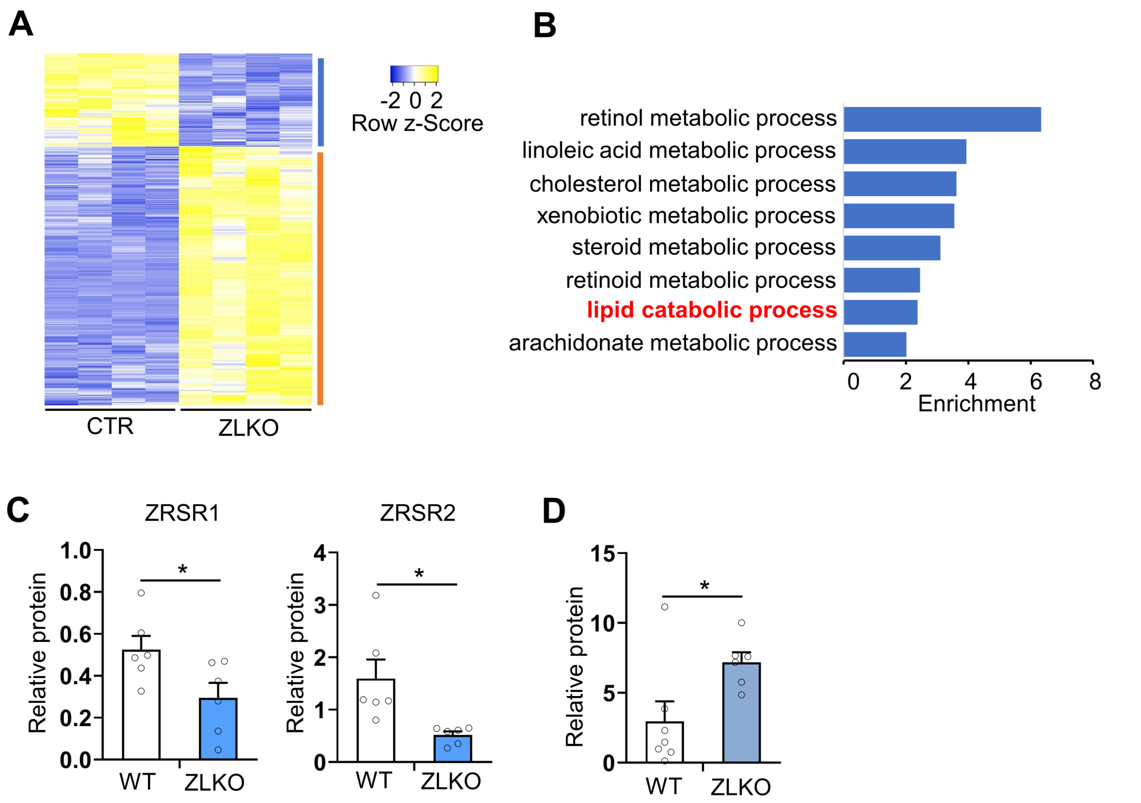

Figure S4. The expression profile and enriched pathways of differential expressed genes in ZLKO mice compared to CTR after chow-diet feeding and the quantification of ZRSR1 and ZRSR2 and cleaved SREBP1c levels. (**A**) Heatmap showing significant upregulated and downregulated >1.4-fold genes in the livers of ZLKO (*n*=4) livers compared to those in CTR (*n*=4) after chow-diet feeding for 1 week. (**B**) Gene Ontology analysis showing enriched pathways associated with genes downregulated in ZLKO mice on chow diet. (**C**) The relative density of the western blot bands of ZRSR1 and ZRSR2 that is normalized to HSP90, related to Figure 2F. (**D**) The relative density of the western blot bands of cleaved SREBP1c that is normalized to HSP90, related to Figure 2I. The data are presented as the mean ± SEM. *p<0.05 by two-tailed unpaired Student's t-test (C-D).

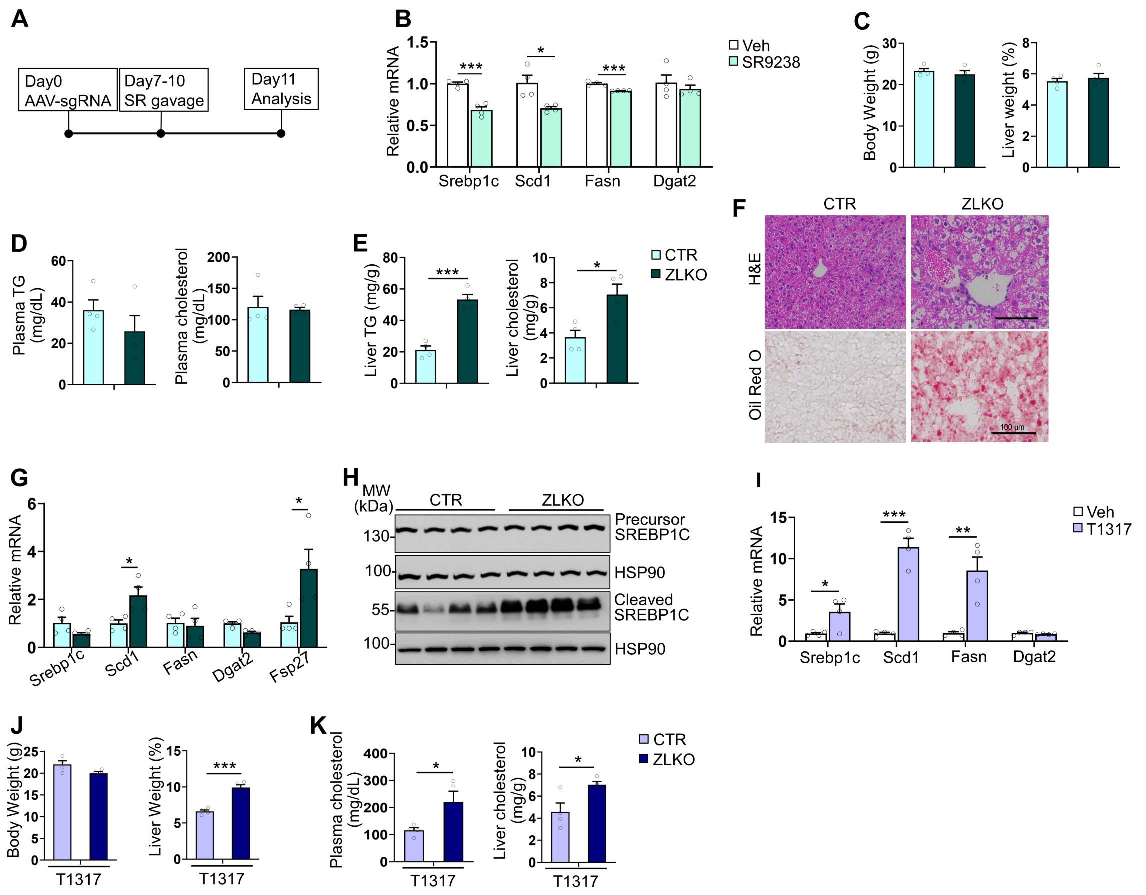

Figure S5. Metabolic phenotypes of Zrsr1 and Zrsr2 double-deficient mice upon LXR suppression or activation.

(**A**) Diagram of the study design. (**B**) QPCR analysis of lipogenesis-associated genes in the livers of wild-type mice receiving oral gavage of vehicle (Veh, *n*=4) or 20 mg/kg/day SR9238 (SR, *n*=4) for 4 days. (**C**) Body weights and percentage of liver weights of CTR (*n*=4) and ZLKO (*n*=4) mice after SR treatment for 4 days. (**D-E**) Plasma (**D**) and liver (**E**) triglyceride (TG) and cholesterol contents of CTR and ZLKO mice after SR treatment. (**F**) Hematoxylin and eosin (H&E) staining and Oil Red O staining (scale bar=100 μm) of livers from CTR and ZLKO mice after SR treatment. (**G**) QPCR analysis of lipogenesis-associated genes in the livers of CTR and ZLKO mice after SR treatment. (**H**) Western blot analyses of liver lysates from CTR and ZLKO mice after SR treatment. (**I**) QPCR analysis of lipogenesis-associated genes in the livers of wild type mice receiving oral gavage of vehicle (Veh, *n*=4) or 25 mg/kg/day T0901317 (T1317, *n*=4) for 4 days.

(**J**) Body weights and percentages of liver weights of CTR (*n*=4) and ZLKO (*n*=4) mice after T1317 treatment. (**K**) Plasma (left) and liver (right) cholesterol contents of CTR and ZLKO mice after T1317 treatment. The data are presented as the mean ± SEM. $*p<0.05$, $**p < 0.01$, $***p < 0.001$ by two-tailed unpaired Student's t-test (B-E, G, and I-K).

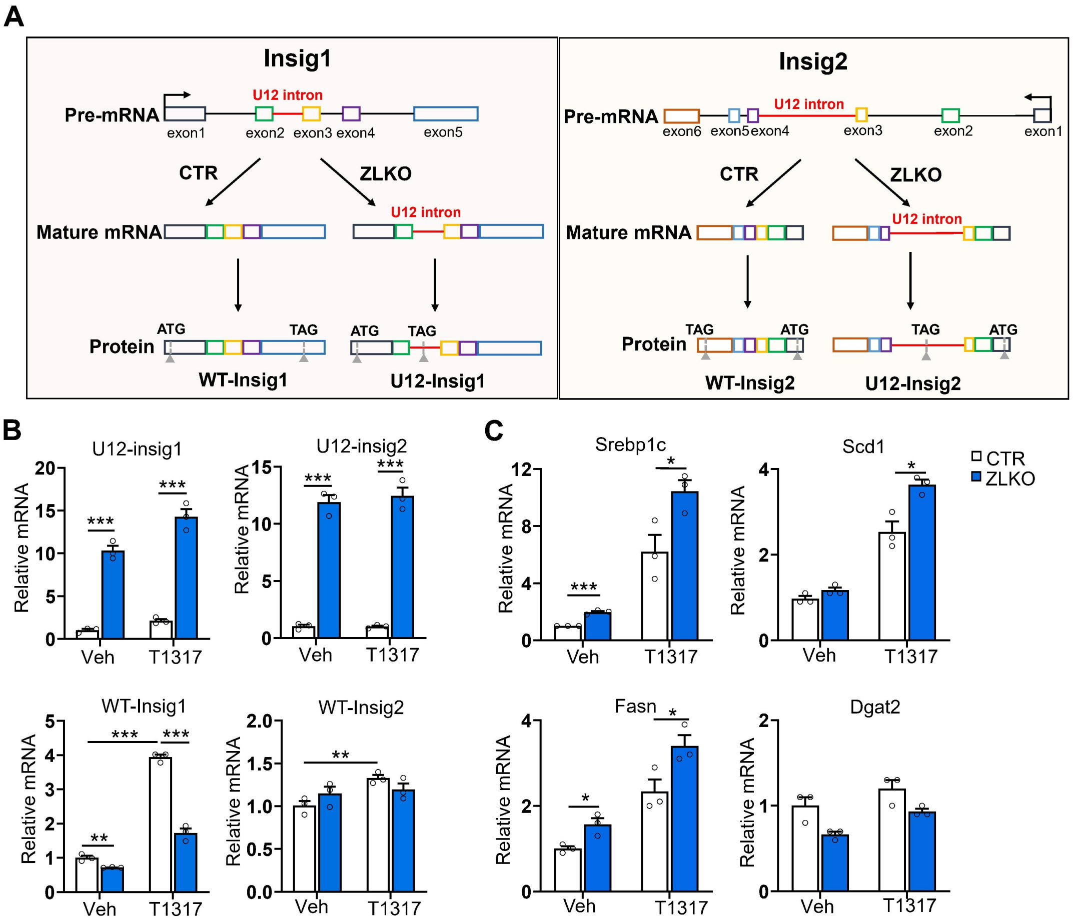

Figure S6. A diagram depicting the minor intron retention of *Insig1* and *Insig2* and depletion of Zrsr1 and Zrsr2 in primary hepatocytes induces *de novo* lipogenic gene expression and minor intron retention of *Insig1* and *Insig2*.

(**A**) Diagram depicting the minor intron retention of *Insig1* and *Insig2* genes in the livers of ZLKO mice results in the early termination of translation of Insig1 and Insig2. (**B**) QPCR analysis of minor (U12) intron expression of Insig1 and Insig2 and the mRNA levels of wild-type (WT) Insig1 and Insig2 in primary hepatocytes (n=3) isolated from CTR and ZLKO livers treated with vehicle (Veh) or T0901317 (T1317, 5 μM) for 24 h. (**C**) QPCR analysis of lipogenic genes in primary hepatocytes (n=3) isolated from CTR and ZLKO livers treated with Veh or T1317 (5 μM) for 24 h. The data in B-C are presented as the means ± SEM. *p<0.05, **p < 0.01, ***p < 0.001, two-tailed unpaired Student's t-test.

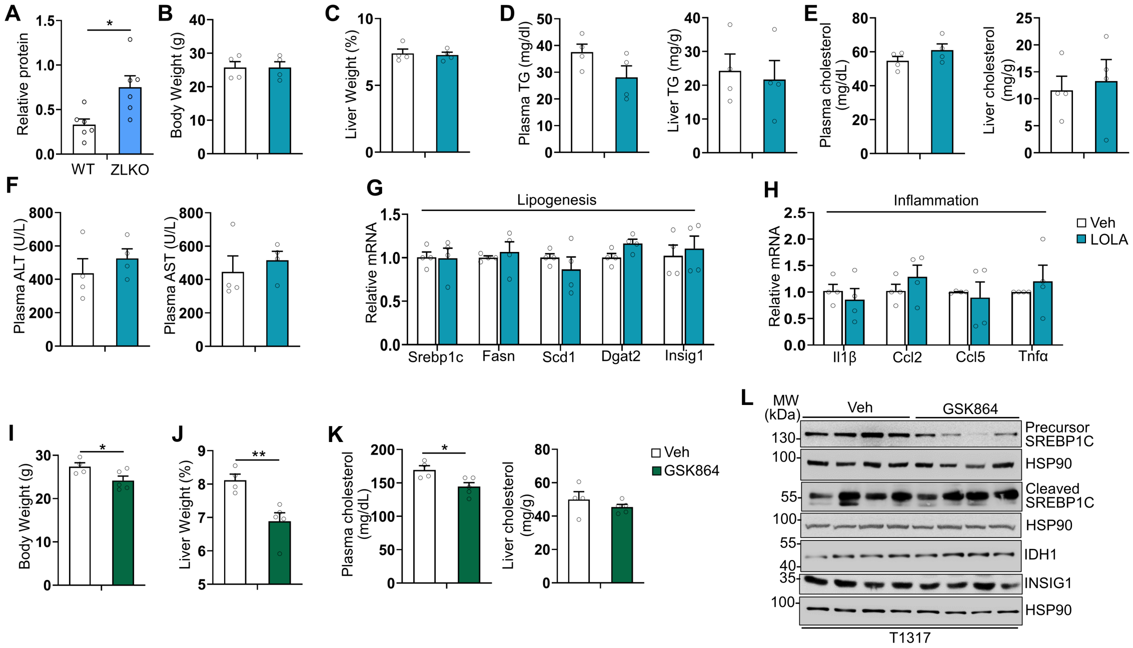

Figure S7. The quantification of IDH1 level and metabolic phenotypes after clearance of hepatic ammonia and inhibition of IDH1 activity in Zrsr1 and Zrsr2 double-deficient mice upon LXR activation.

(**A**) The relative density of the western blot bands of IDH1 that is normalized to HSP90, related to Figure 4O. (**B**-**C**) Body weights (**B**) and percentage of liver weights (**C**) of ZLKO mice daily injected with saline (*n*=4) or 2 g/kg L-ornithine-aspartate (LOLA, *n*=4) for 17 days and then combined with an oral administration of T0901317 (T1317, 25mg/kg/day) for 4 days. (**D**-**E**) Plasma and liver triglyceride (TG) (**D**) and cholesterol (**E**) levels in Veh- and LOLA-treated ZLKO mice combined with T1317 treatment. (**F**) Plasma ALT and AST levels in Veh- and LOLA-treated ZLKO mice combined with T1317 treatment. (**G**-**H**) QPCR analysis of hepatic genes involved in lipogenesis and inflammation in Veh- and LOLA-treated ZLKO mice combined with T1317 treatment. (**I**-**J**) Body weights (**I**) and percentage of liver weights (**J**) of ZLKO mice intraperitoneally injected with Veh (*n*=4) or GSK864 (75 mg/kg/day, *n*=5) combined with an oral administration of T1317 (25 mg/kg/day) for 4 days. (**K**) Plasma and liver cholesterol content in Veh- and GSK864-treated ZLKO mice combined with T1317 treatment. (**L**) Immunoblotting of liver lysates from Veh- and GSK864-treated ZLKO mice

combined with T1317 treatment. The data are presented as the means ± SEM. $^{*}p<0.05$, $^{**}p < 0.01$ by two-tailed unpaired Student's t-test (A-K).

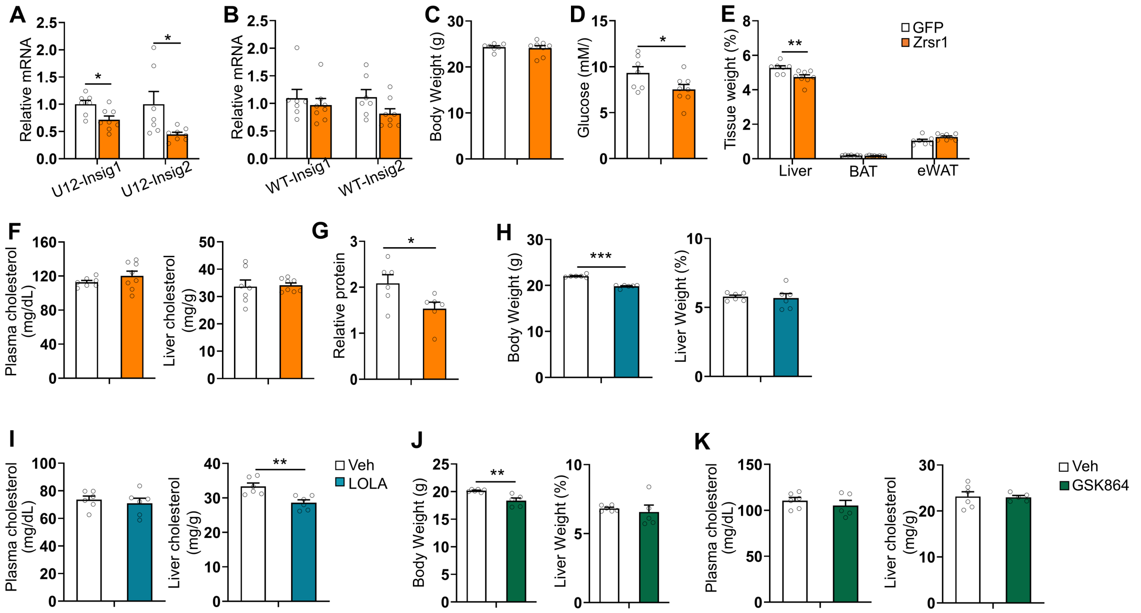

Figure S8. Metabolic phenotypes in mice after restoring minor intron splicing activity or suppressing IDH1-ammonia axis upon MASH diet feeding and the quantification of IDH1 level

(**A**-**B**) QPCR analysis of minor (U12) introns in Insig1 and Insig2 (**A**) and the mRNA levels of wild type (WT) Insig1 and Insig2 (**B**) in the livers of mice injected with AAV-TBG-GFP (GFP, *n*=7) or AAV-TBG-Zrsr1 (Zrsr1, *n*=8) after CDA-HFD (MASH diet) feeding for 8 weeks. (**C**-**E**) Body weights (**C**), blood glucose level (**D**) and percentage of tissue weights (**E**) were measured in GFP- and Zrsr1-expressing mice. (**G**) The relative density of the western blot bands of IDH1 that is normalized to HSP90, related to Figure 6F. (**F**) Plasma (left) and liver (right) cholesterol content in GFP- and Zrsr1-expressing mice. (**H**) Body weights (left) and percentage of liver weights (right) in wild type mice daily gavaged with saline (*n*=6) or 2 g/kg L-ornithine-aspartate (LOLA, *n*=6) and both fed on MASH diet for 8 weeks. (**I**) Plasma (left) and liver (right) cholesterol content in Veh- and LOLA-treated wild type mice after MASH diet feeding. (**J**) Body weights (left) and percentage of liver weights (right) in wild type mice daily intraperitoneally injected with vehicle (Veh, *n*=6) or 75 mg/kg GSK864 (*n*=5) and both fed on MASH diet for 8 weeks. (**K**) Plasma (left) and liver (right) cholesterol content in Veh- and GSK864-treated wild type mice after MASH diet feeding. The data are presented

as the mean ± SEM. *p<0.05, **p < 0.01, ***p < 0.001, two-tailed unpaired Student's t-test (A-K).

References

1. Lin J, Wu PH, Tarr PT, Lindenberg KS, St-Pierre J, Zhang CY, et al. Defects in adaptive energy metabolism with CNS-linked hyperactivity in PGC-1alpha null mice. *Cell.* 2004;119(1):121-35.

2. Ma D, Molusky MM, Song J, Hu CR, Fang F, Rui C, et al. Autophagy deficiency by hepatic FIP200 deletion uncouples steatosis from liver injury in NAFLD. *Molecular endocrinology.* 2013;27(10):1643-54.

3. Gutiérrez-de-Juan V, López de Davalillo S, Fernández-Ramos D, Barbier-Torres L, Zubiete-Franco I, Fernández-Tussy P, et al. A morphological method for ammonia detection in liver. *PLoS One.* 2017;12(3):e0173914.

4. Li S, Liu C, Li N, Hao T, Han T, Hill DE, et al. Genome-wide coactivation analysis of PGC-1alpha identifies BAF60a as a regulator of hepatic lipid metabolism. *Cell Metab.* 2008;8(2):105-17.

5. Dobin A, Davis CA, Schlesinger F, Drenkow J, Zaleski C, Jha S, et al. STAR: ultrafast universal RNA-seq aligner. *Bioinformatics.* 2013;29(1):15-21.

6. Anders S, Pyl PT, and Huber W. HTSeq--a Python framework to work with high-throughput sequencing data. *Bioinformatics.* 2015;31(2):166-9.

7. Moyer DC, Larue GE, Hershberger CE, Roy SW, and Padgett RA. Comprehensive database and evolutionary dynamics of U12-type introns. *Nucleic Acids Res.* 2020;48(13):7066-78.

8. Love MI, Huber W, and Anders S. Moderated estimation of fold change and dispersion for RNA-seq data with DESeq2. *Genome biology.* 2014;15(12):550.

9. Pertea M, Pertea GM, Antonescu CM, Chang TC, Mendell JT, and Salzberg SL. StringTie enables improved reconstruction of a transcriptome from RNA-seq reads. *Nat Biotechnol.* 2015;33(3):290-5.

10. Alcoriza-Balaguer MI, García-Cañaveras JC, Benet M, Juan-Vidal O, and Lahoz A. FAMetA: a mass isotopologue-based tool for the comprehensive analysis of fatty acid metabolism. *Brief Bioinform.* 2023;24(2).
