# Supplementary material for: Disrupted minor intron splicing activates reductive carboxylation-mediated lipogenesis to drive metabolic dysfunction–associated steatotic liver disease progression
Source: J Clin Invest. 2025 Mar 18;135(10):e186478. doi: 10.1172/JCI186478 (PMC12077890; doi:10.1172/JCI186478)

Figure 1D

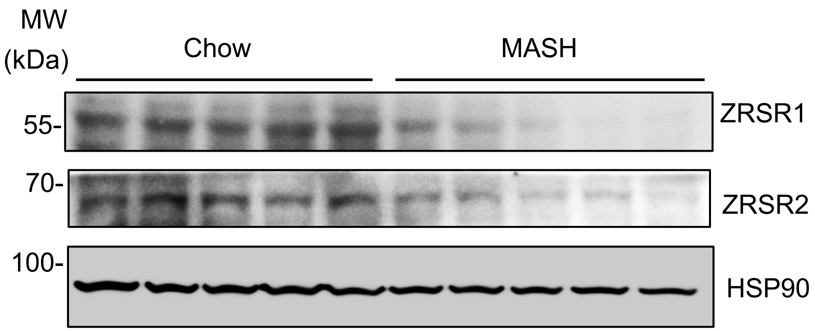

Full unedited blot for Figure 1D

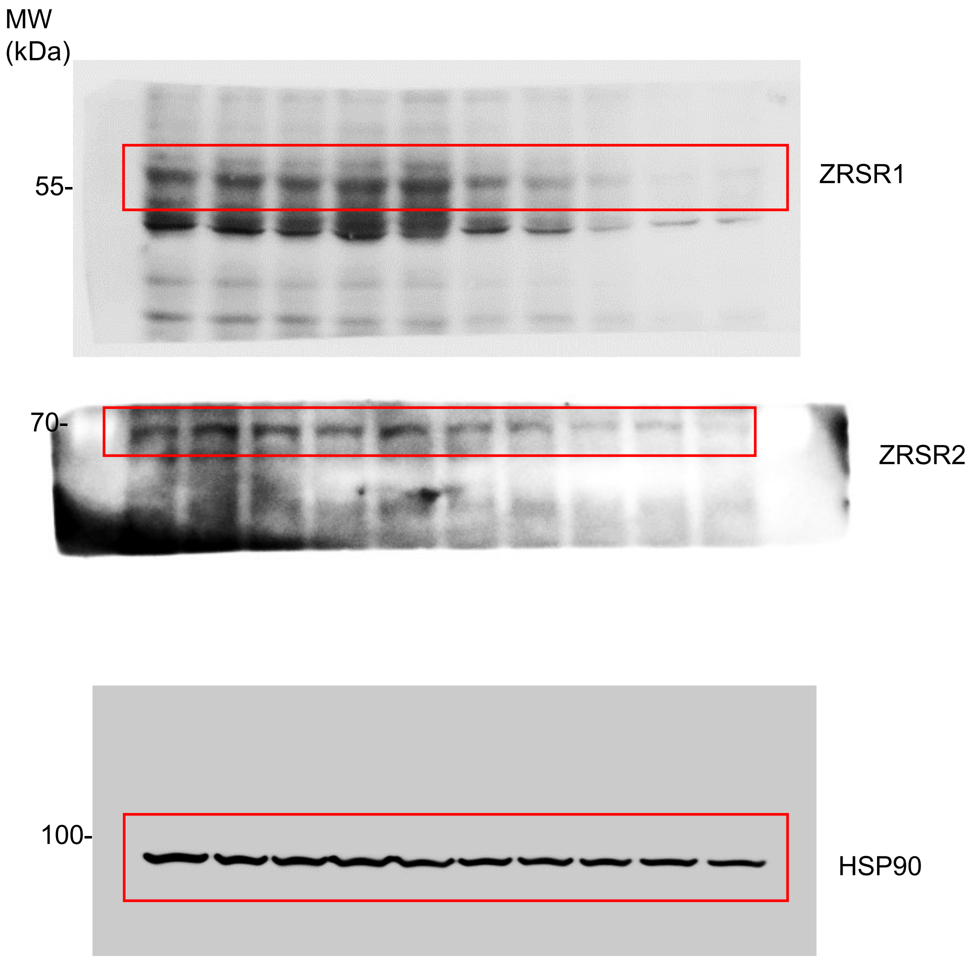

Figure 1G

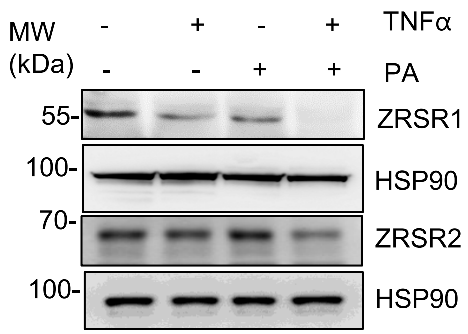

Full unedited blot for Figure 1G

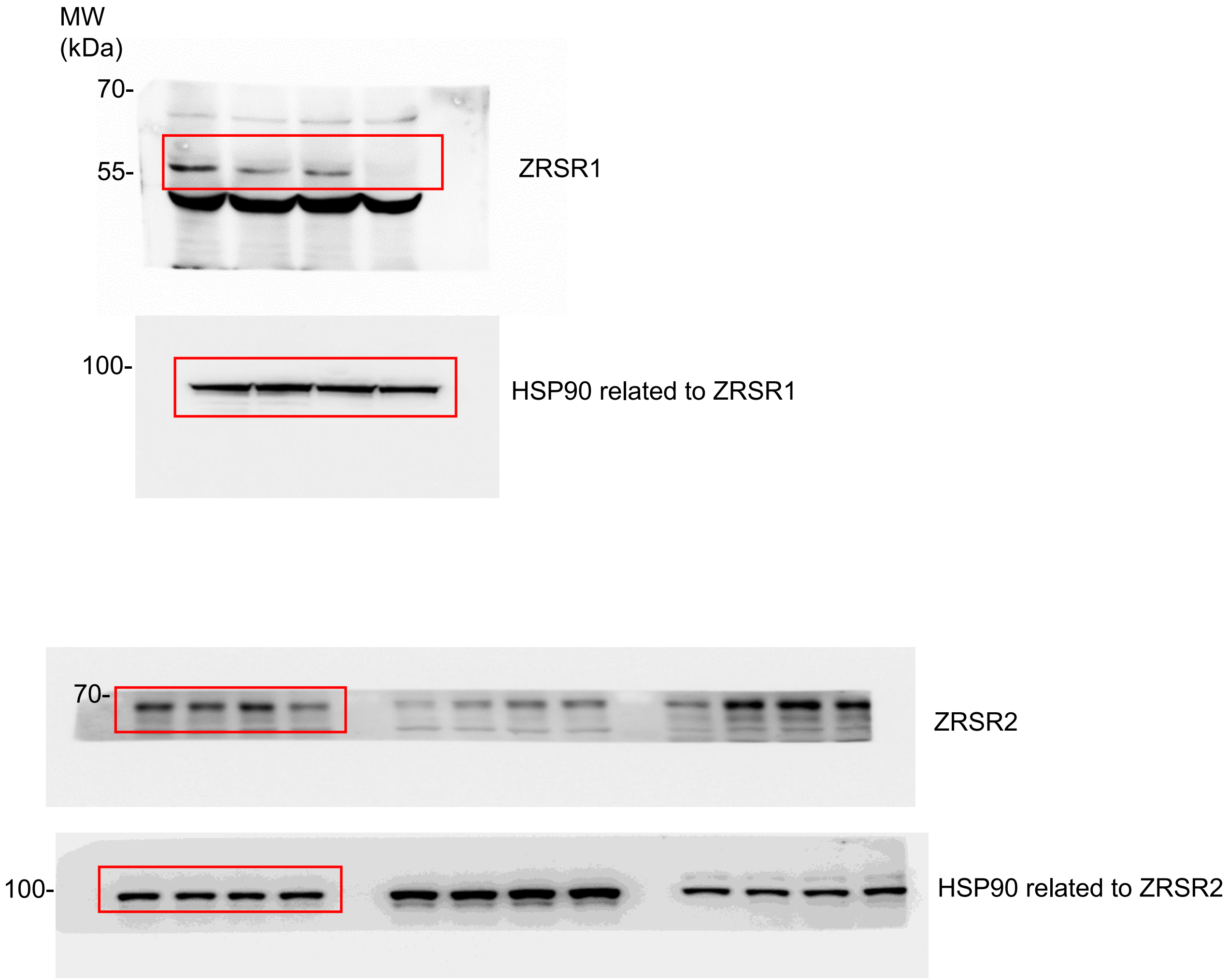

Figure 2F

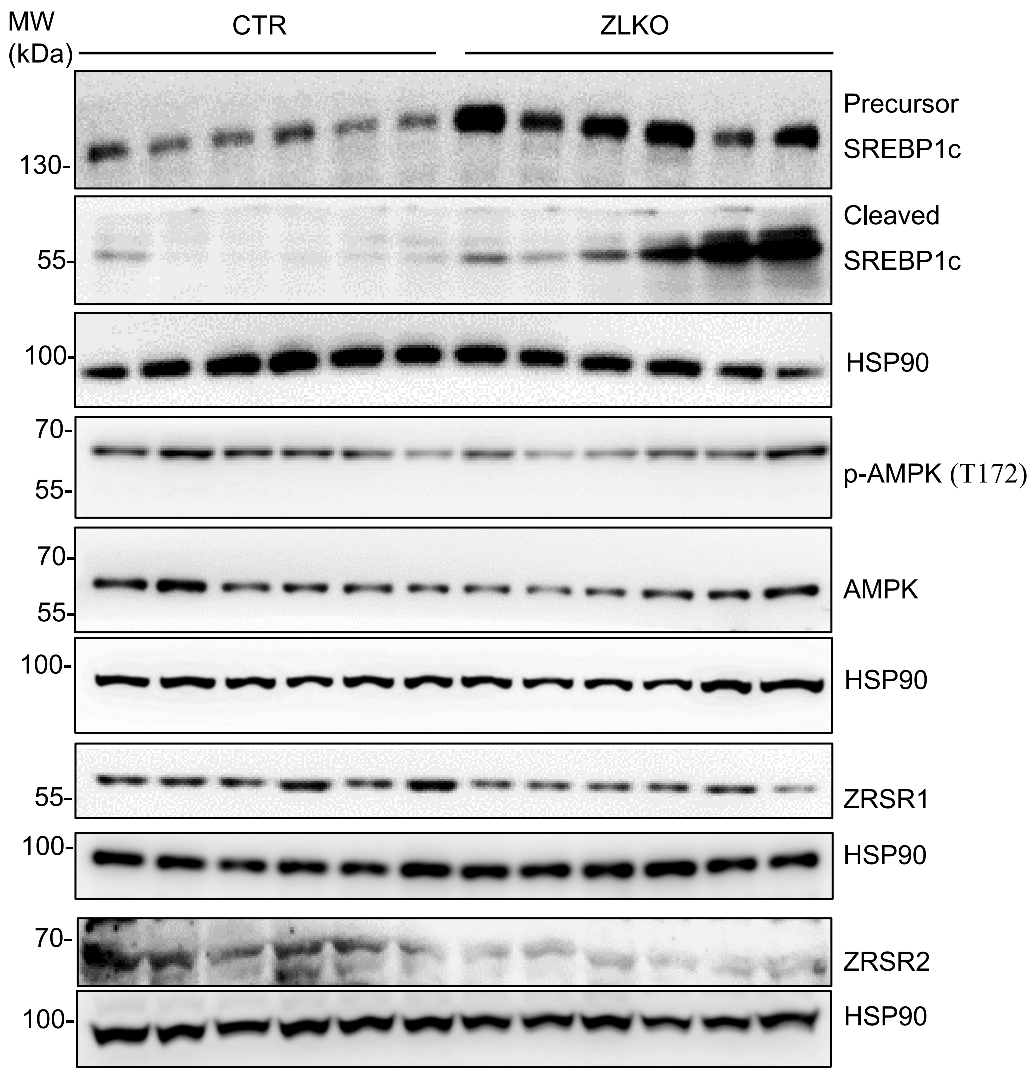

Full unedited blot for Figure 2F

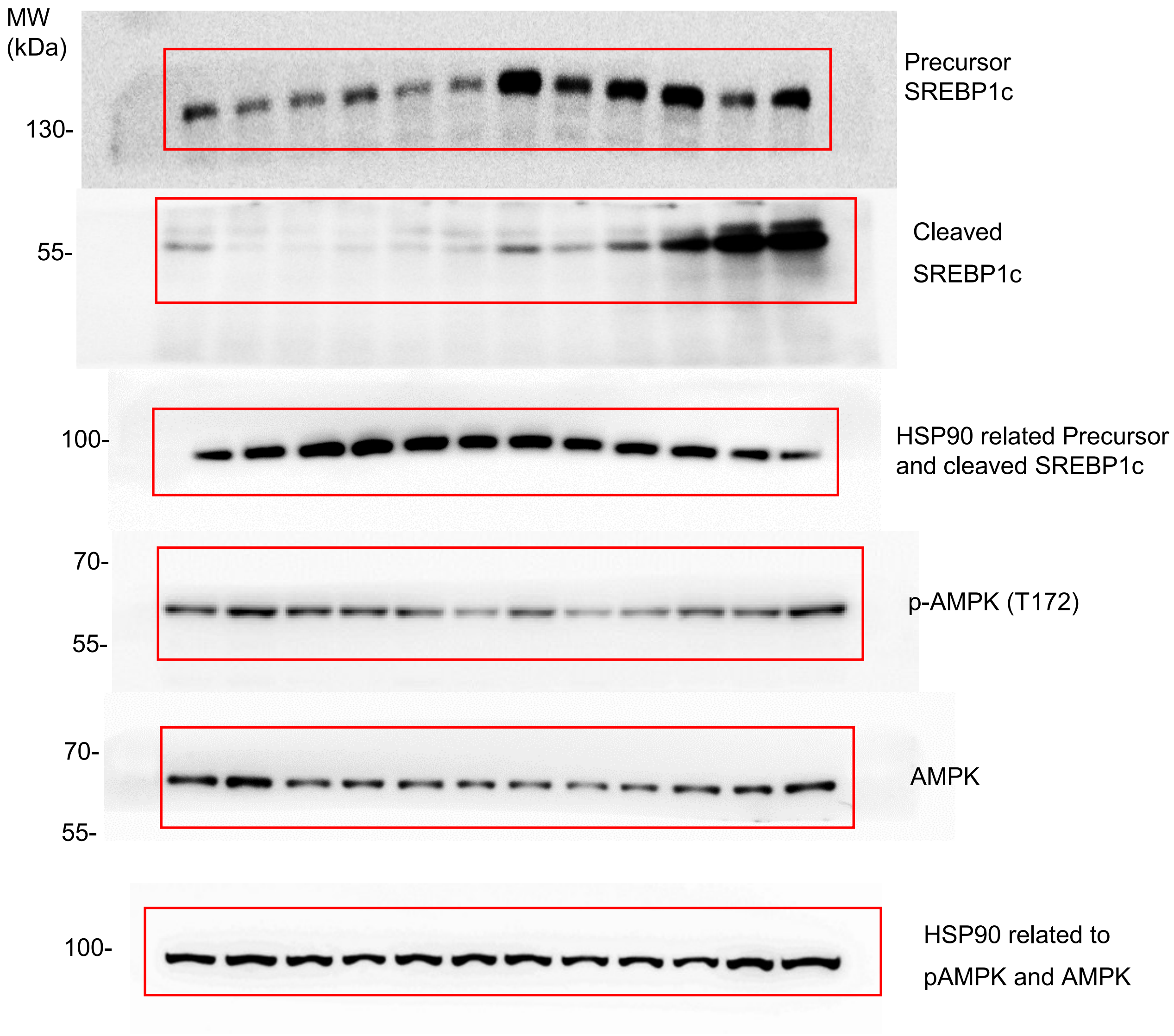

Figure 2F

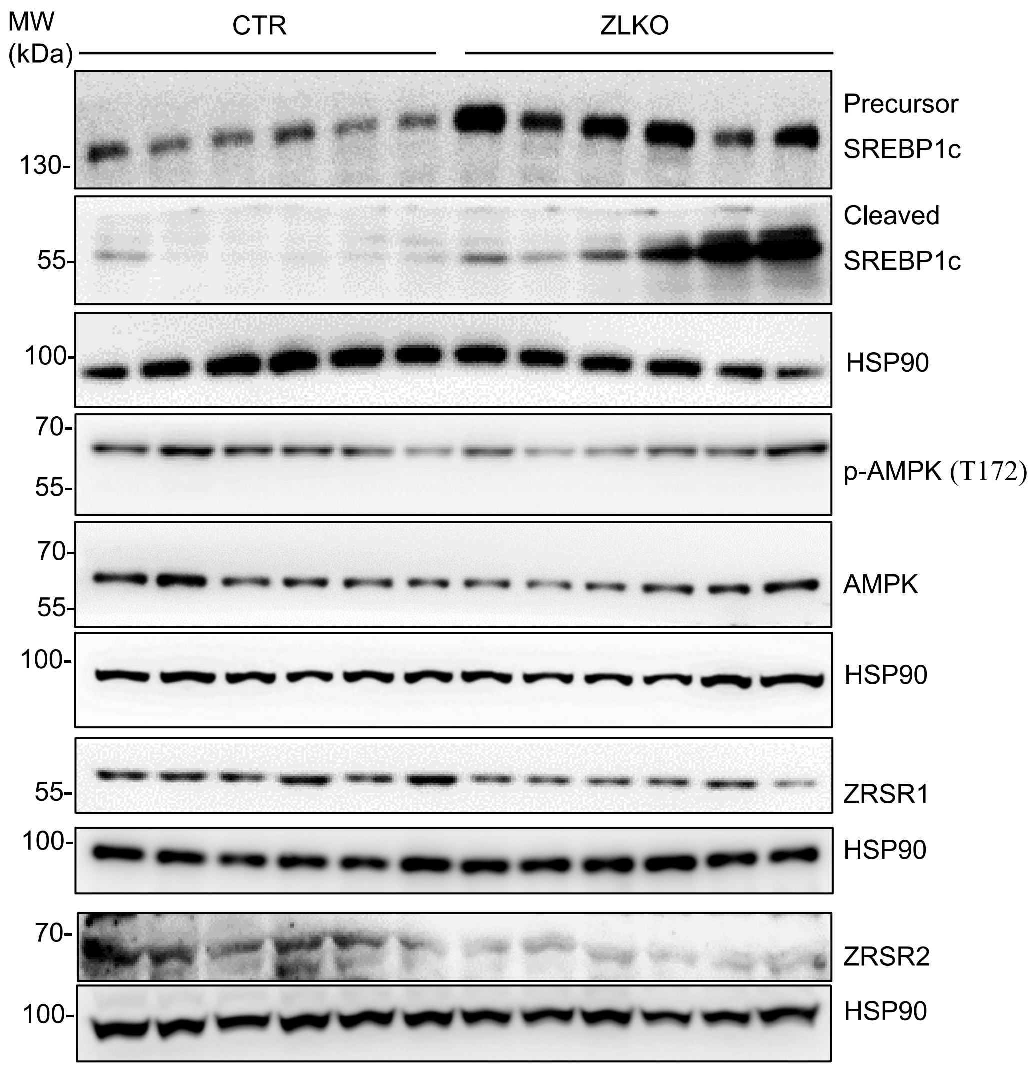

Full unedited blot for Figure 2F

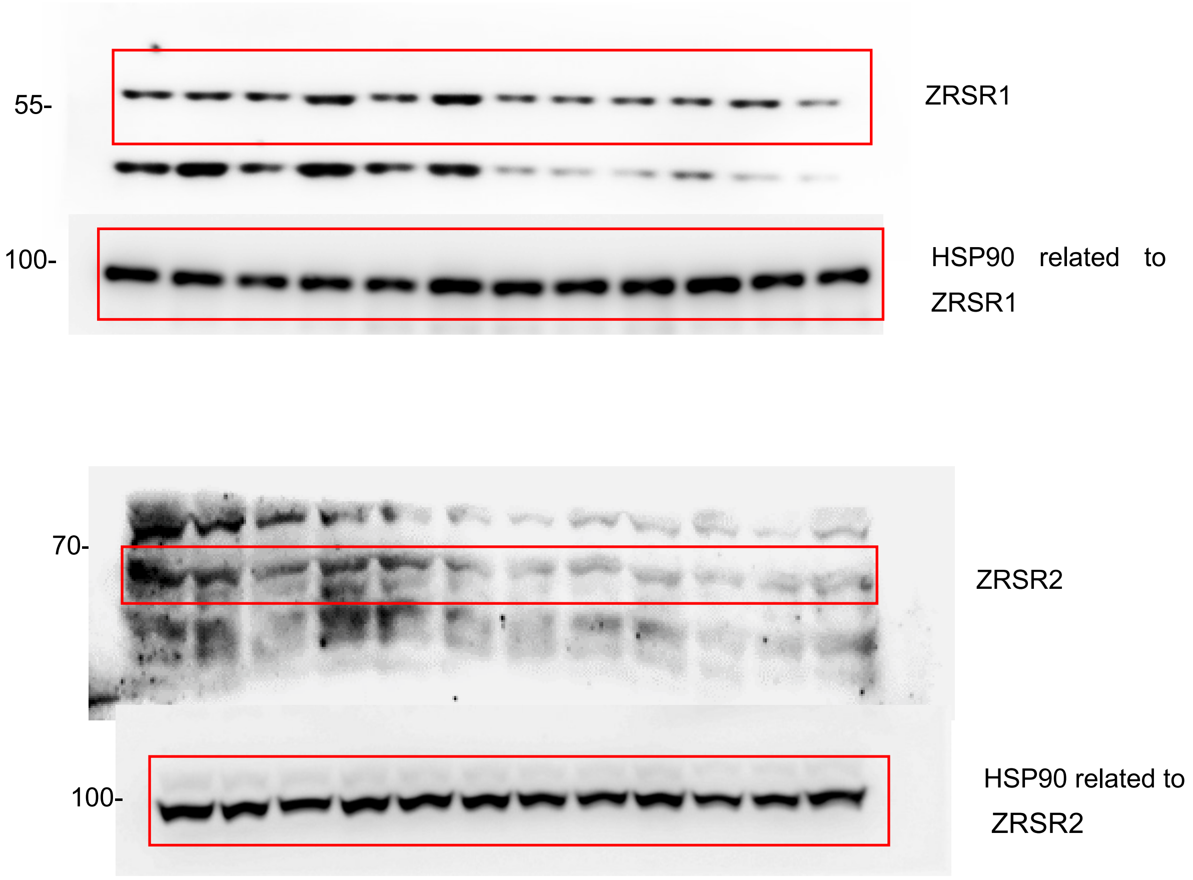

Figure 2I

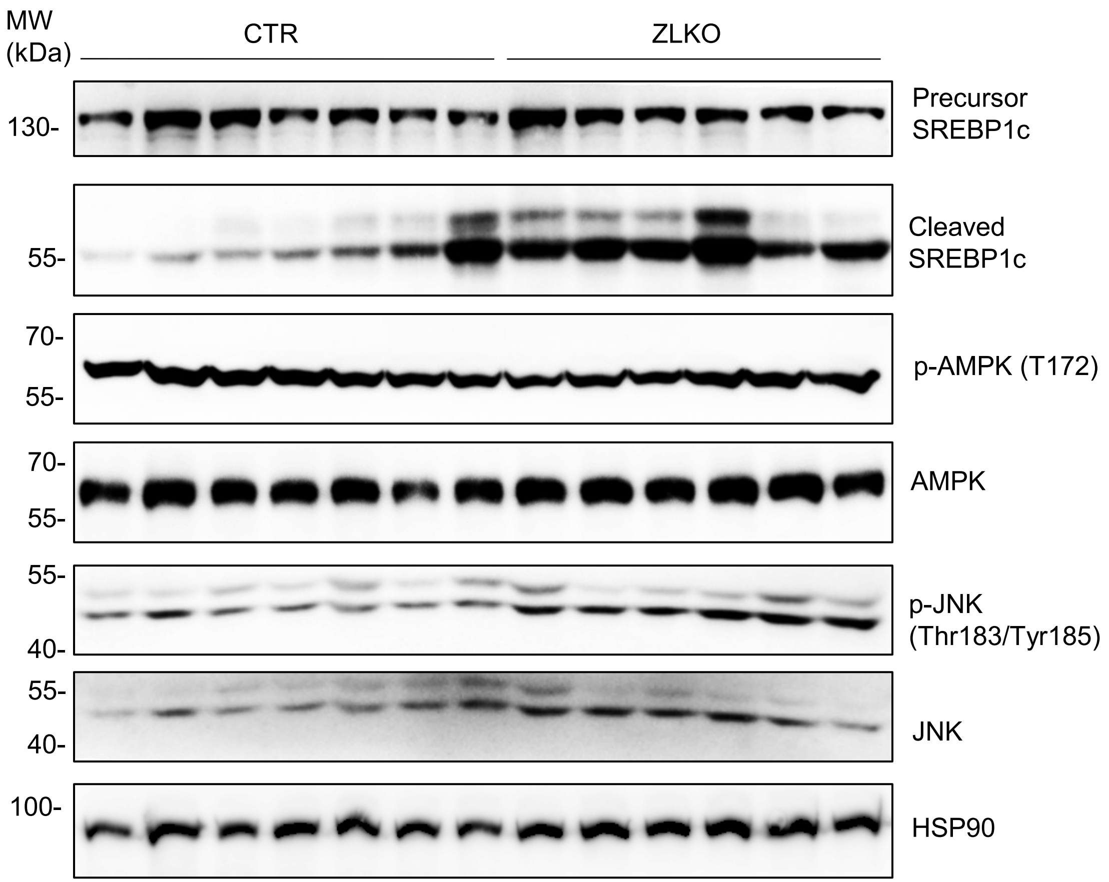

Full unedited blot for Figure 2I

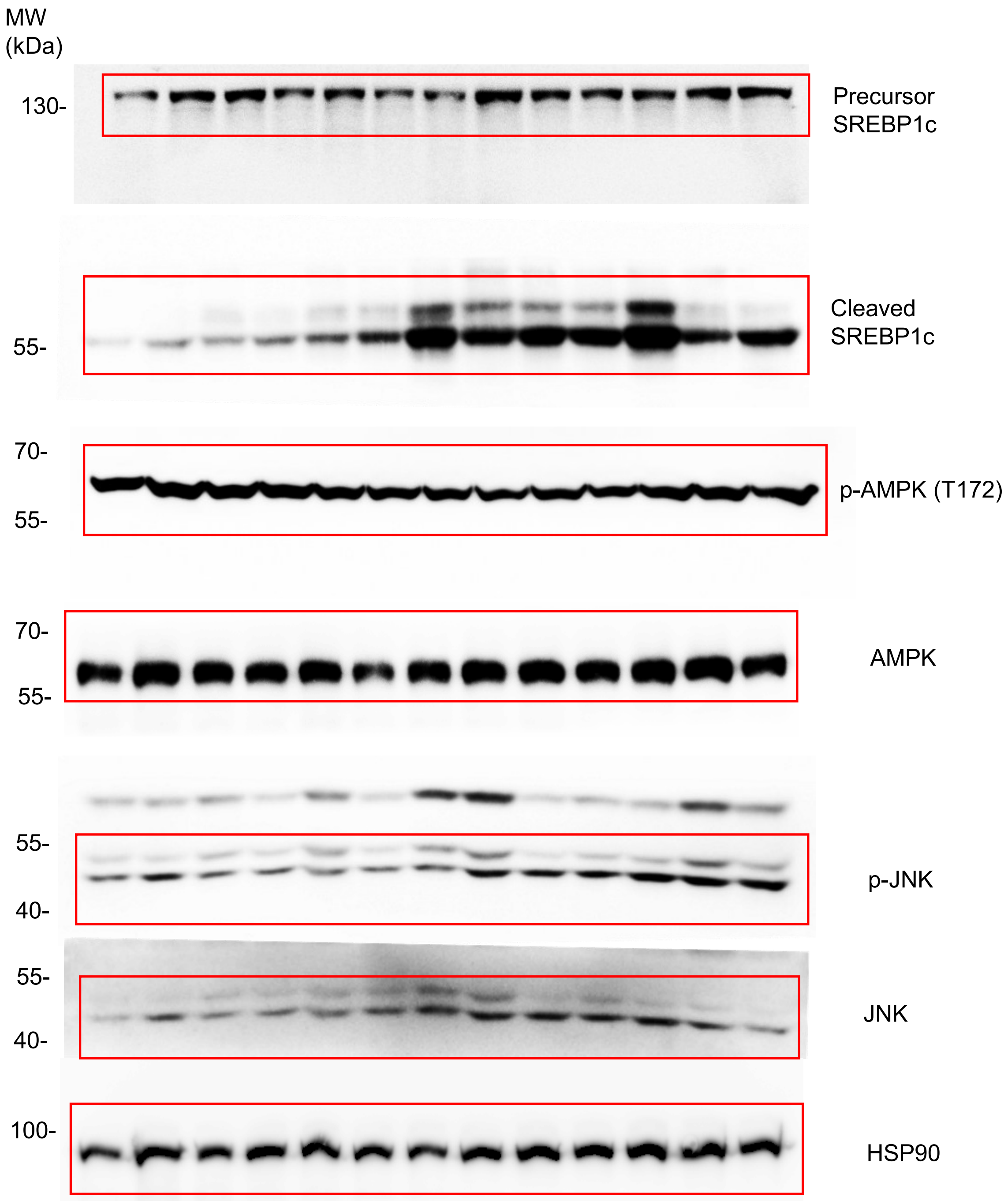

Figure 2O

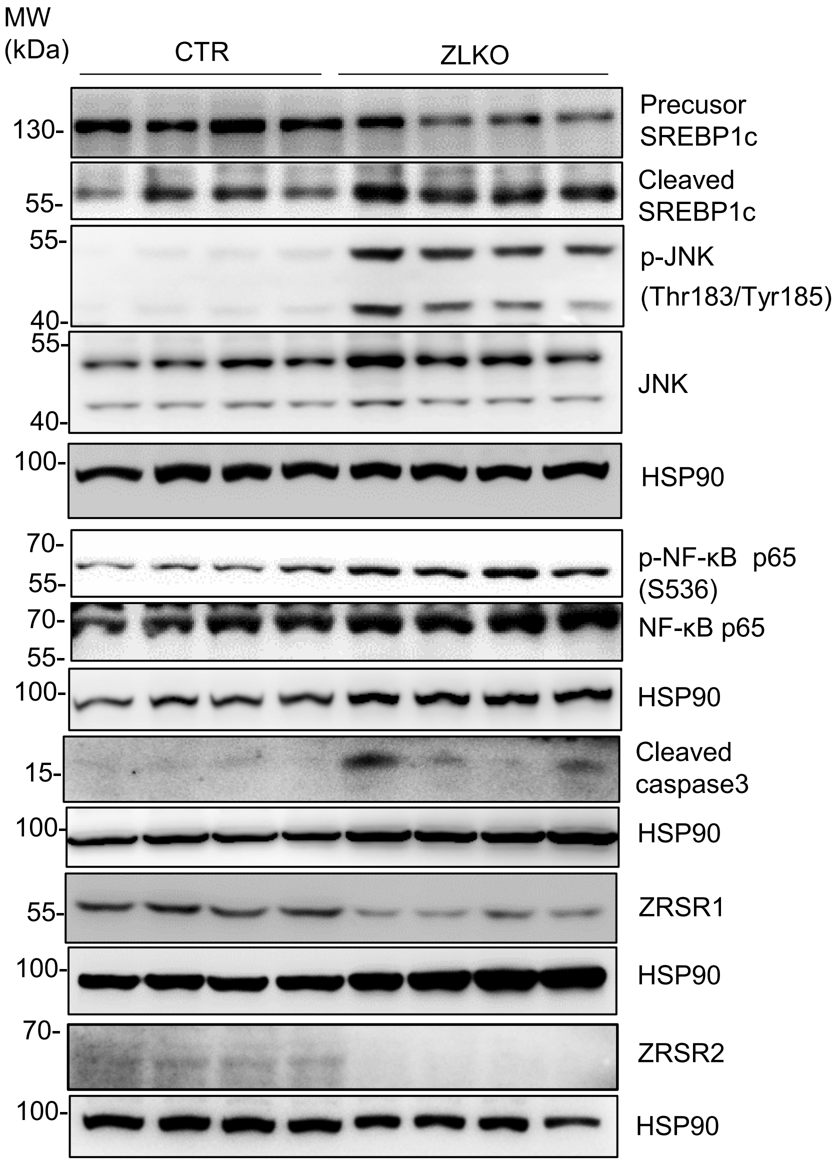

Full unedited blot for Figure 2O

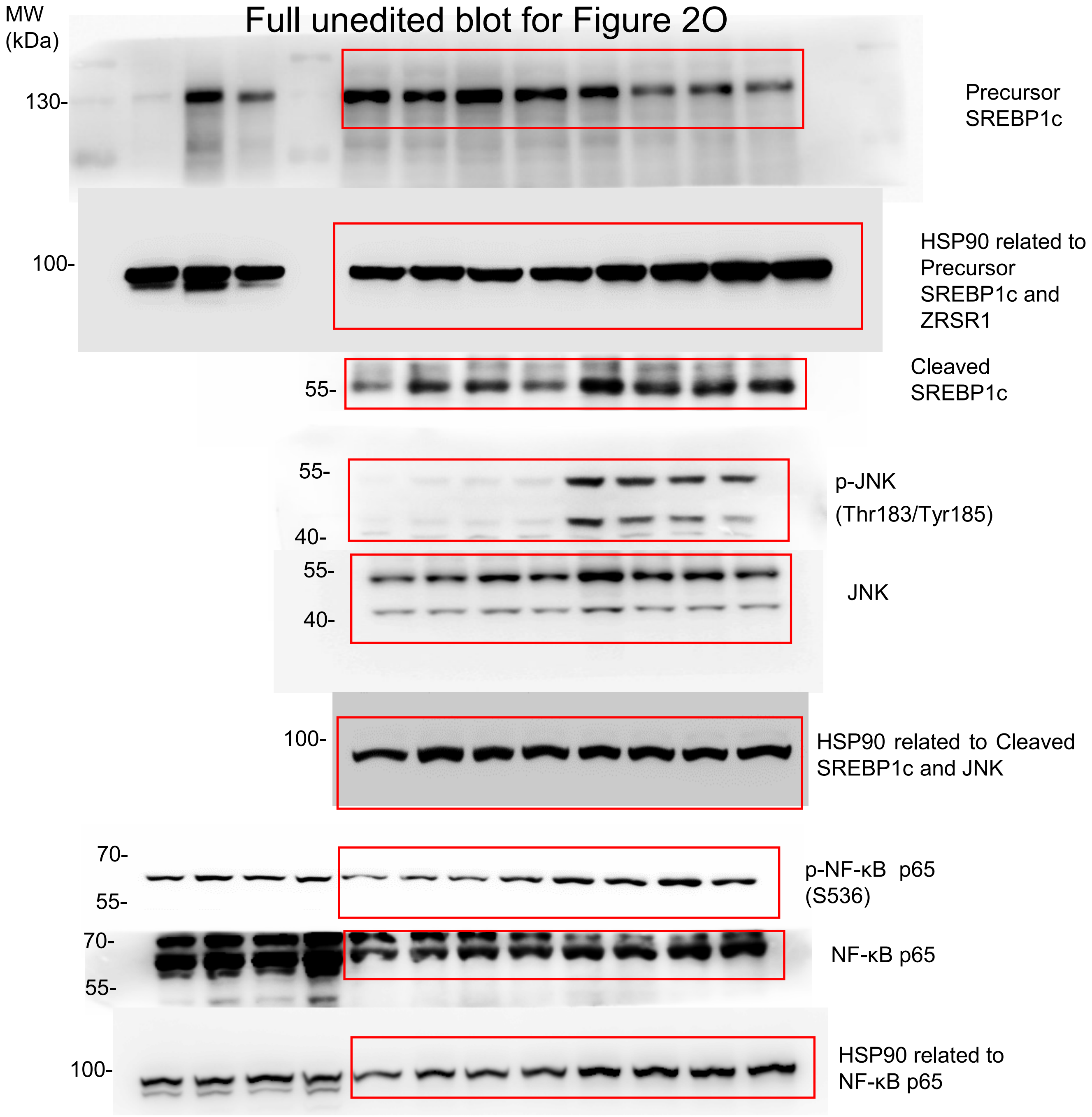

Figure 2O

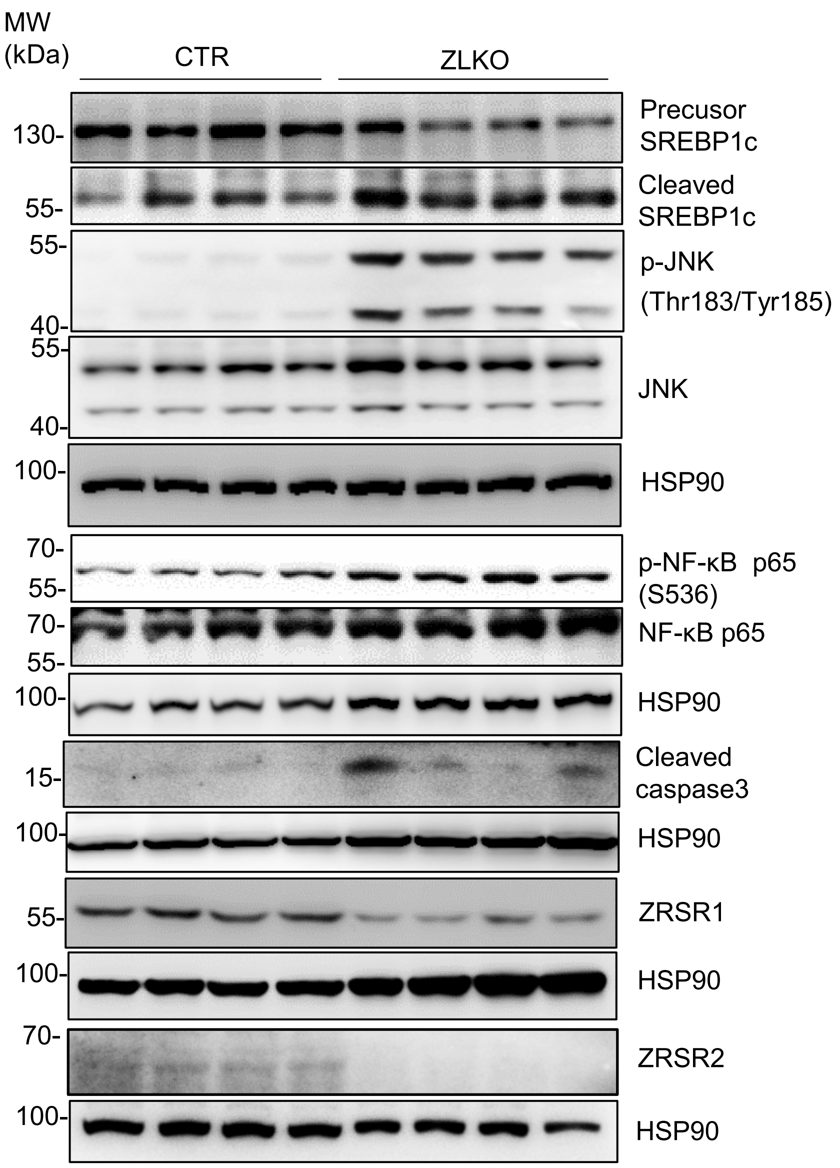

Full unedited blot for Figure 2O

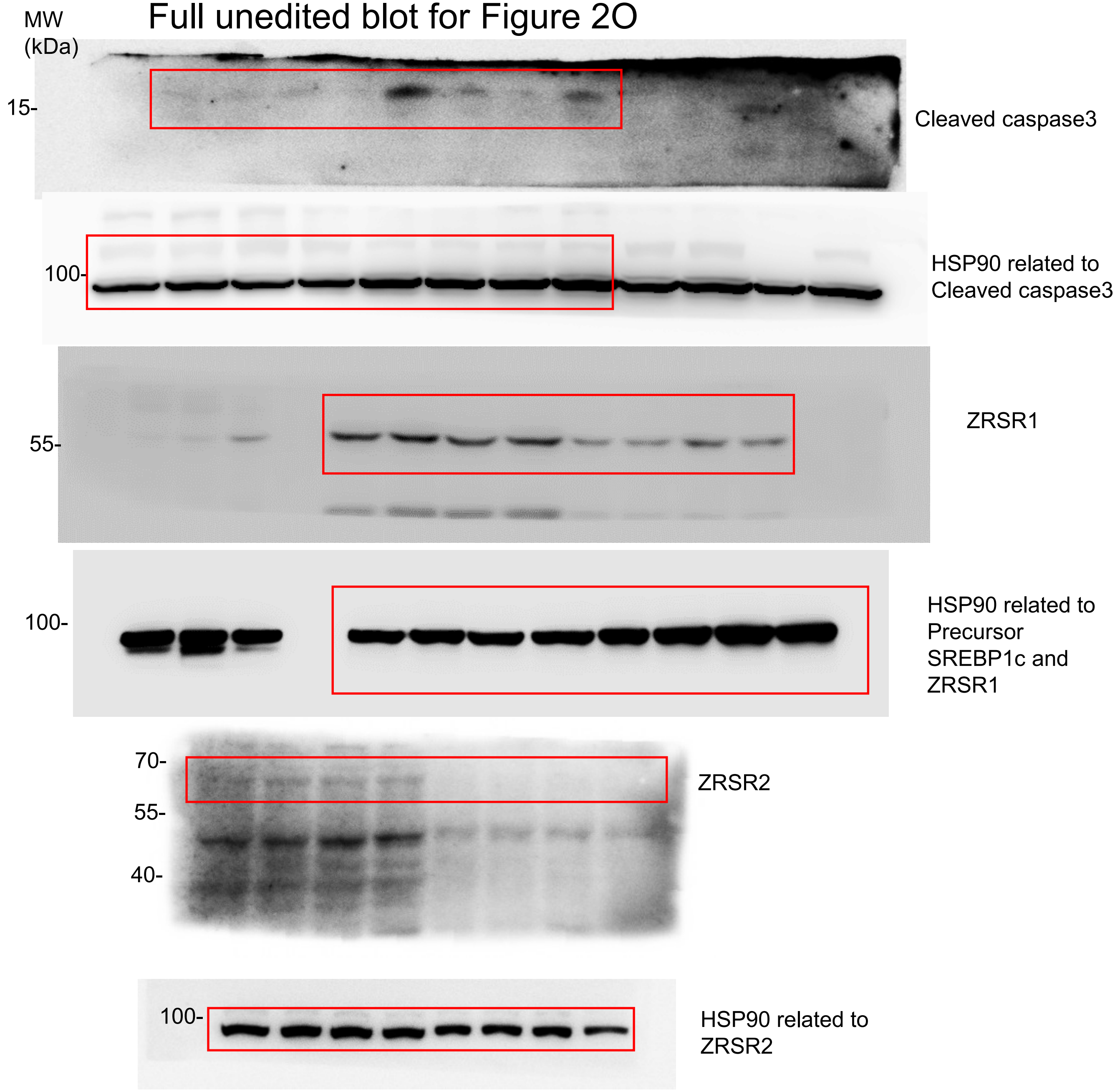

Figure 3I

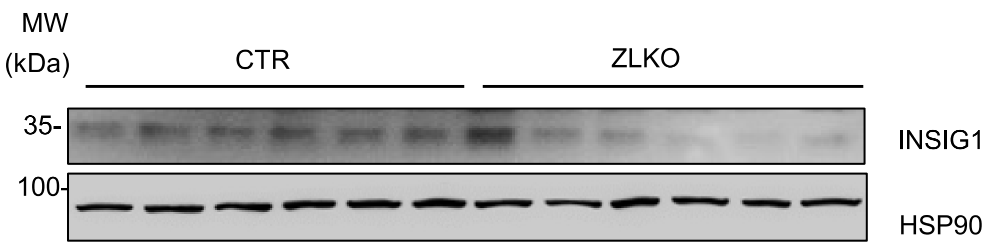

Full unedited blot for Figure 3I

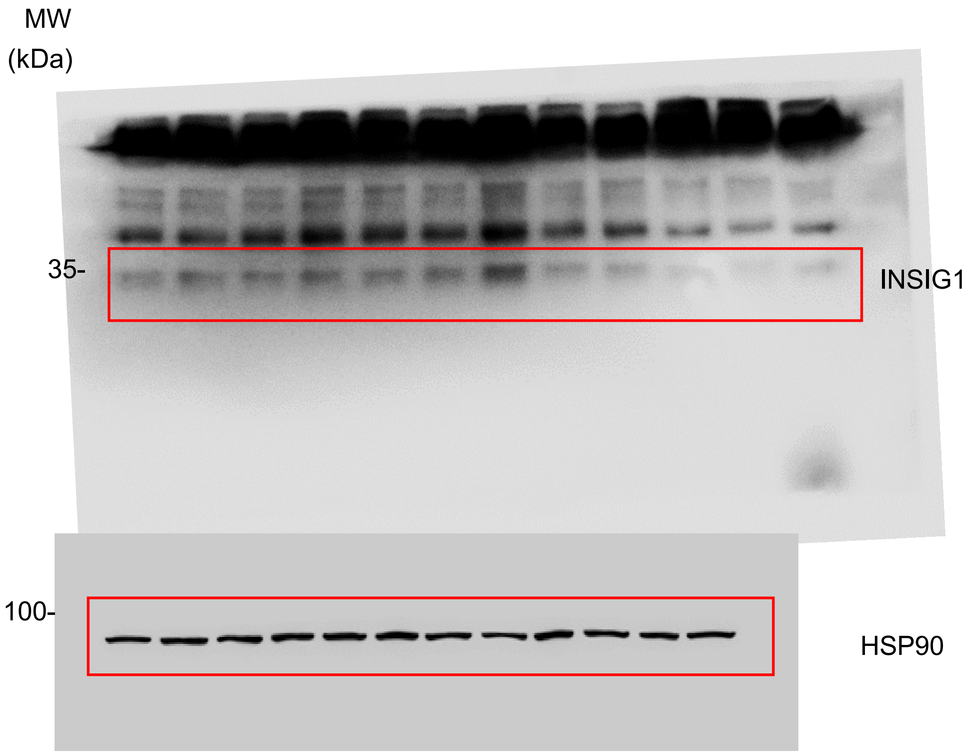

Figure 3L

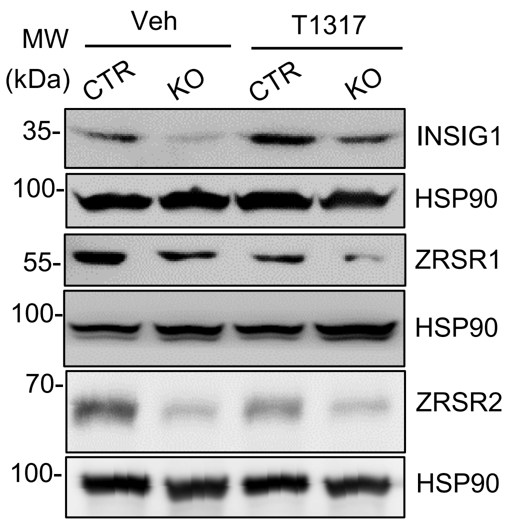

Full unedited blot for Figure 3L

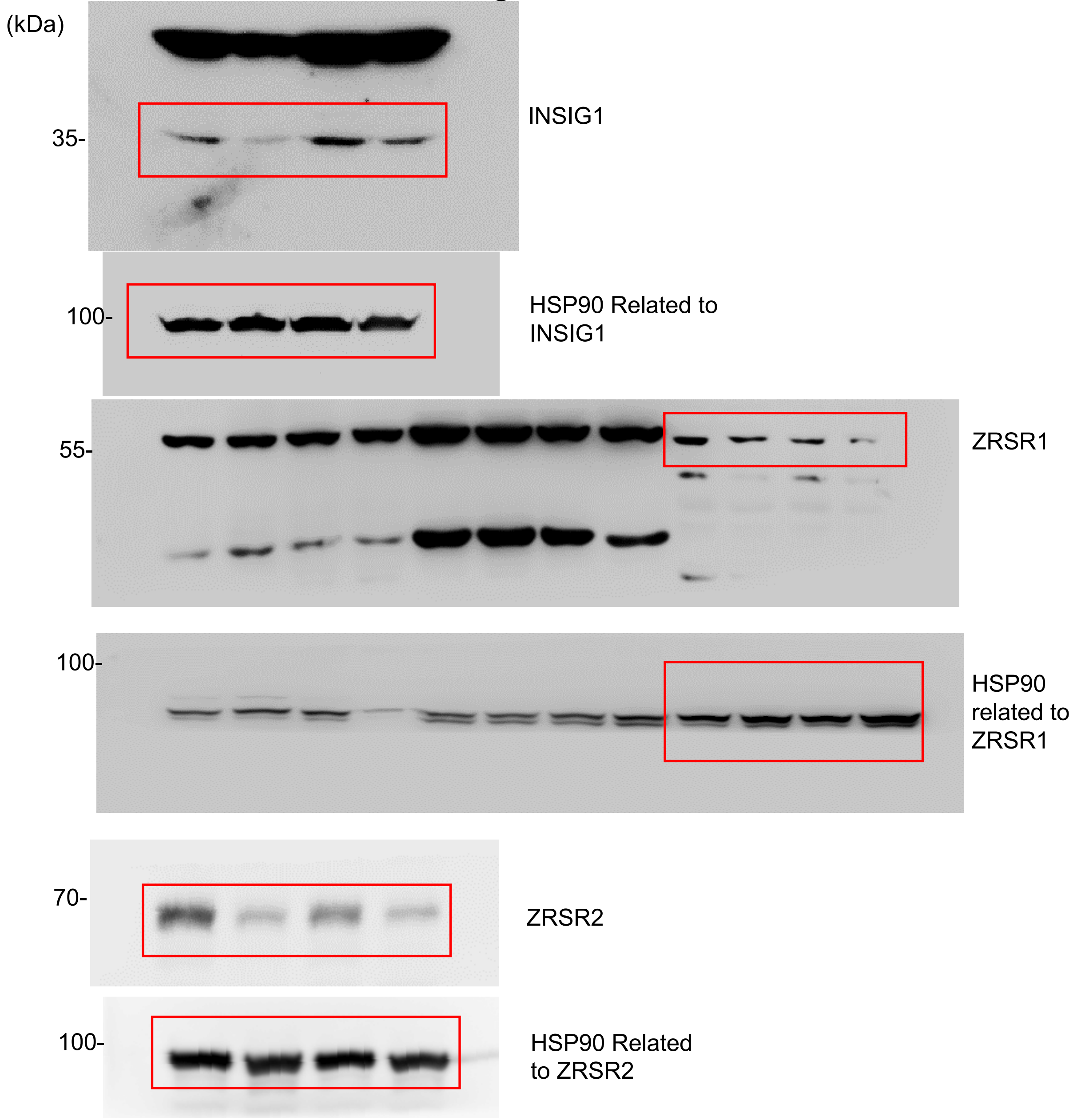

Full unedited blot for Figure 3N

Figure 3N

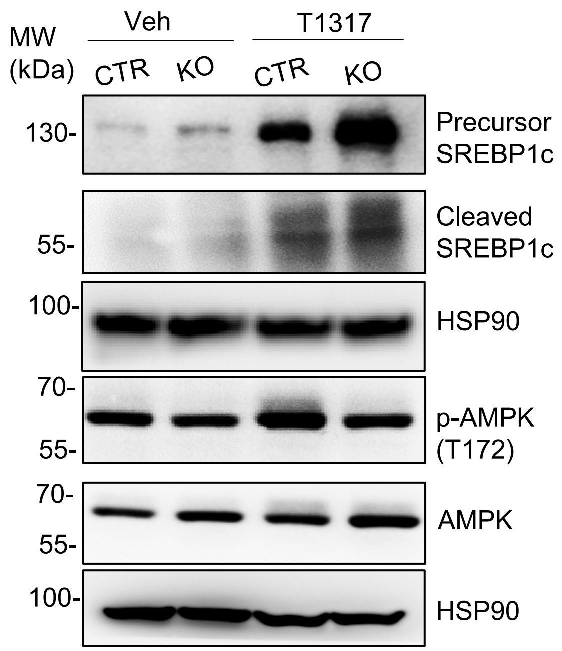

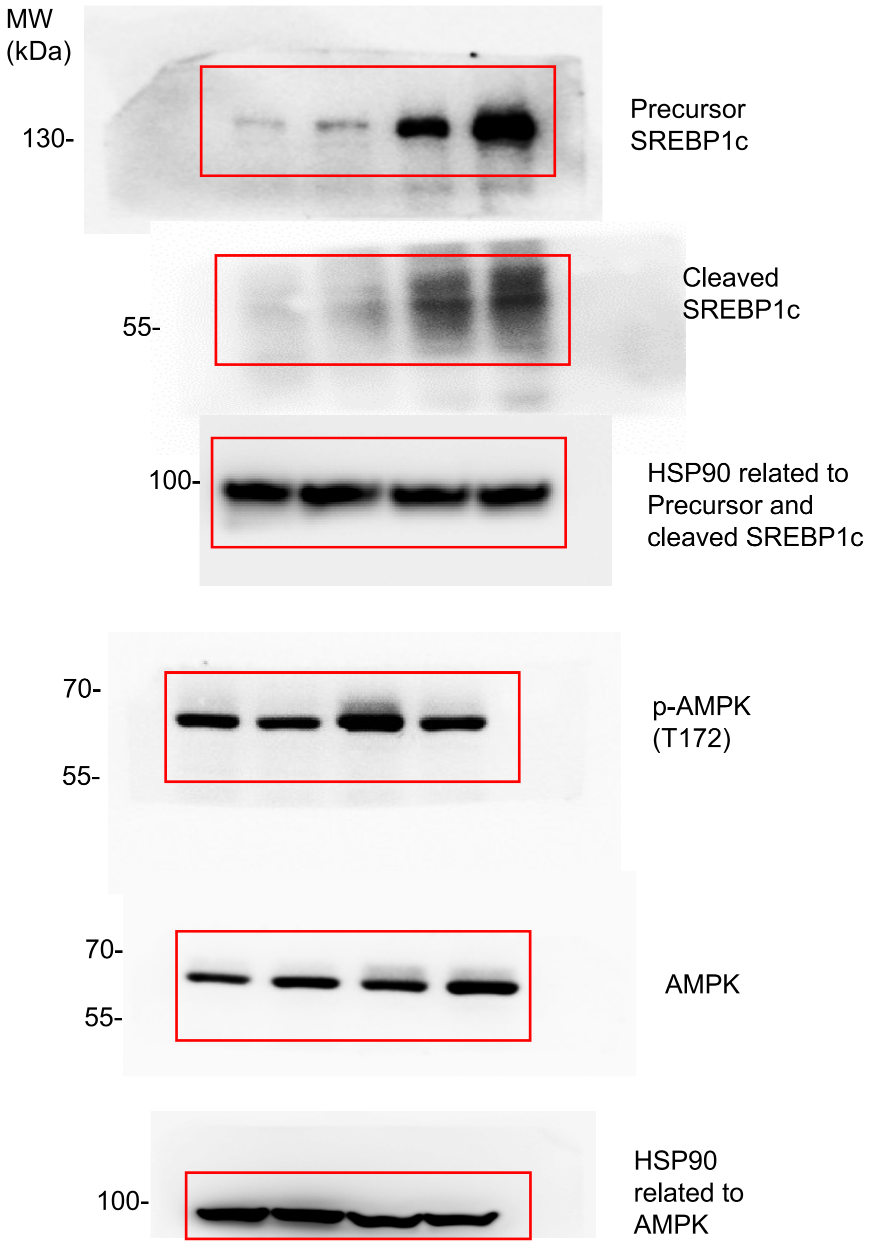

Figure 3P

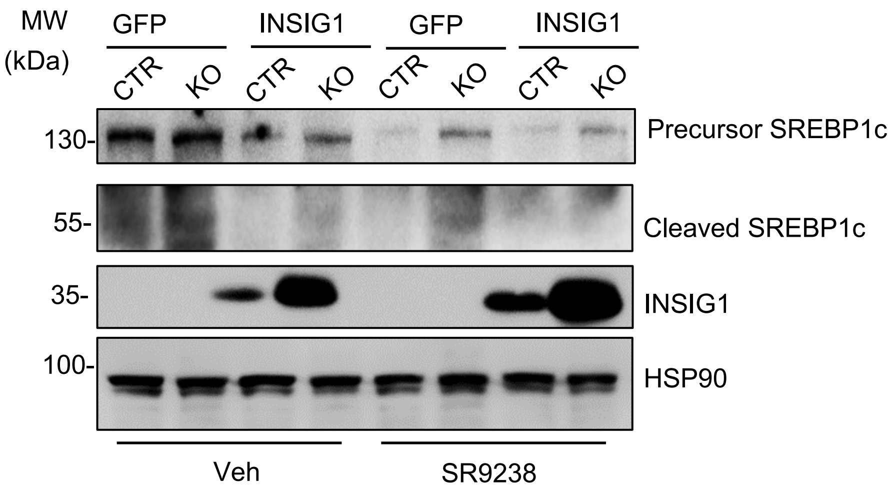

Full unedited blot for Figure 3P

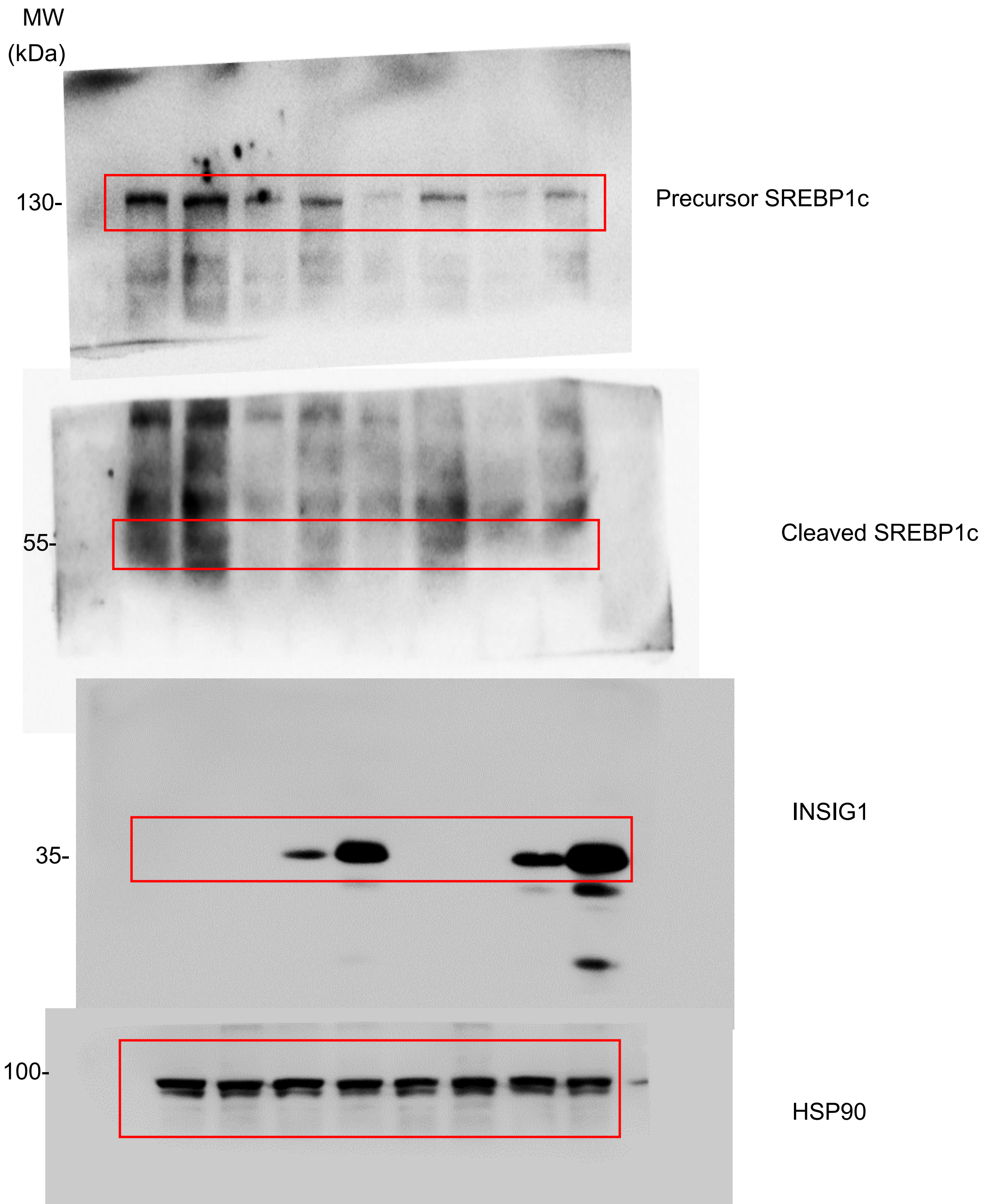

Figure 3S

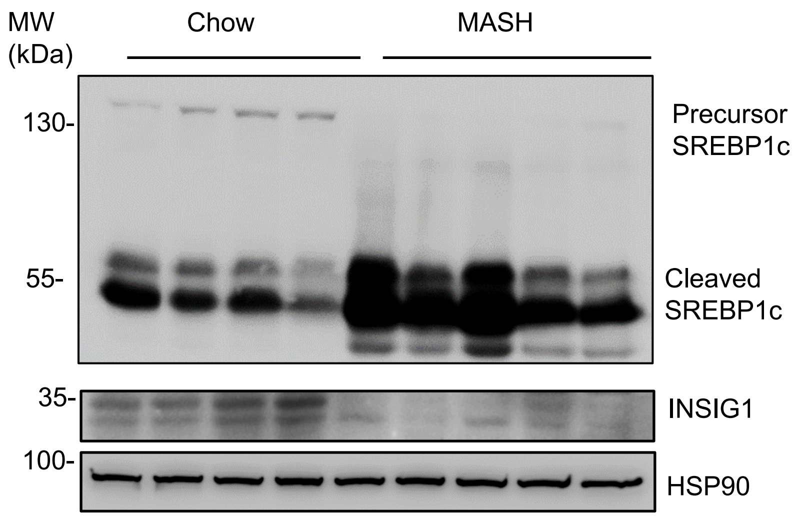

Full unedited blot for Figure 3S

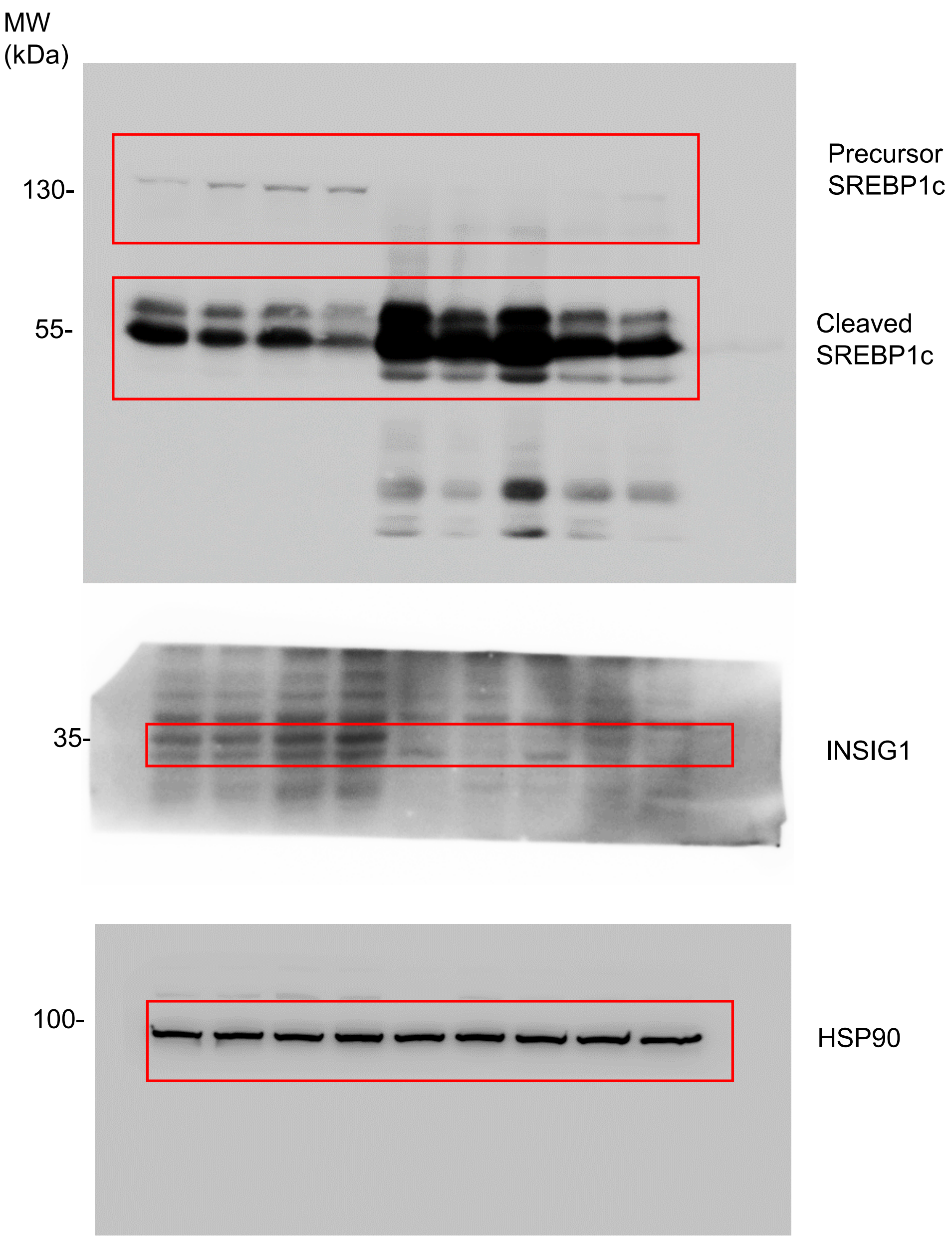

Figure 3V

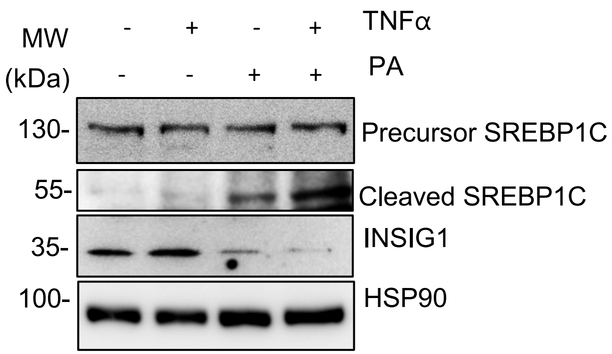

Full unedited blot for Figure 3V

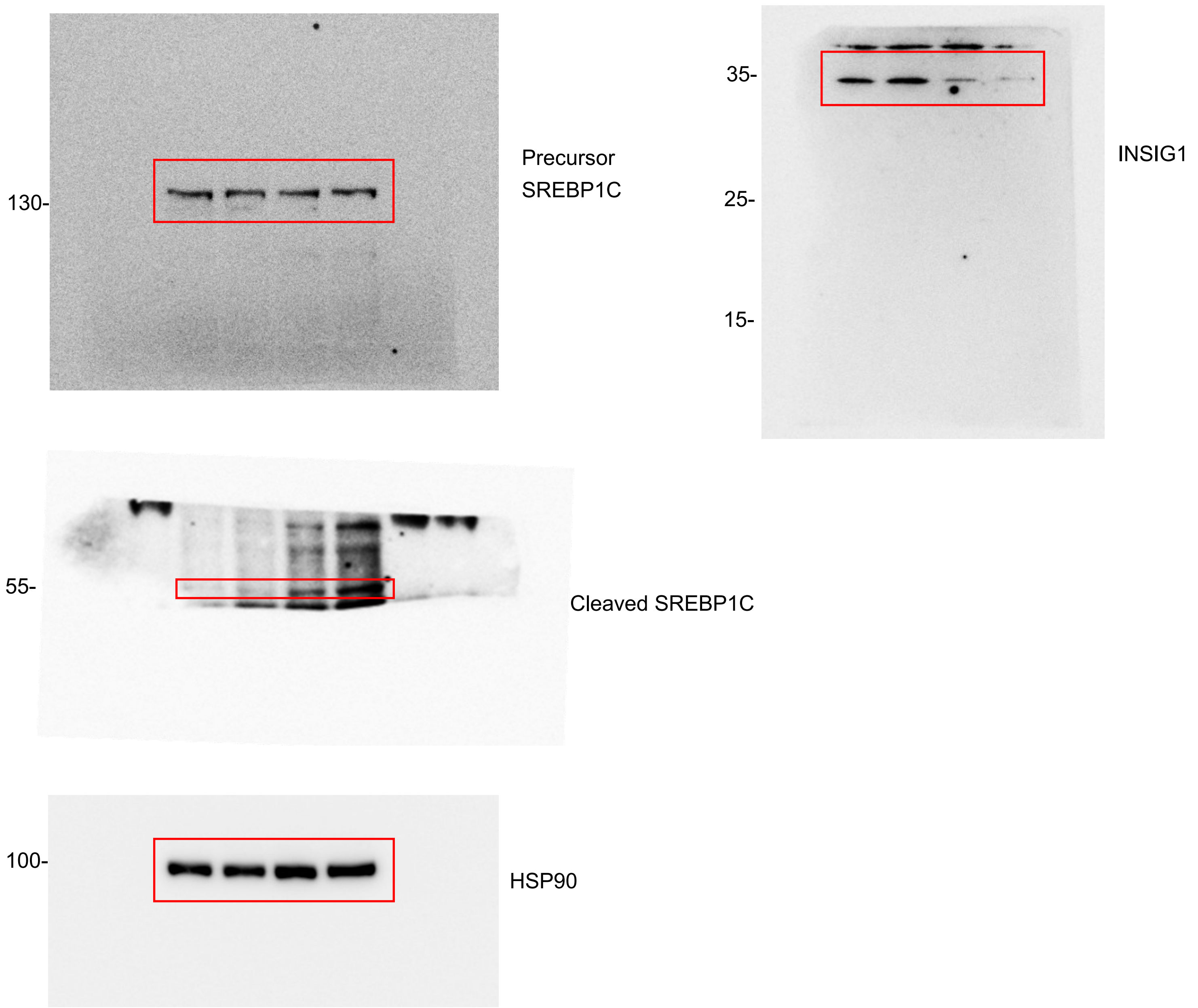

Figure 4O

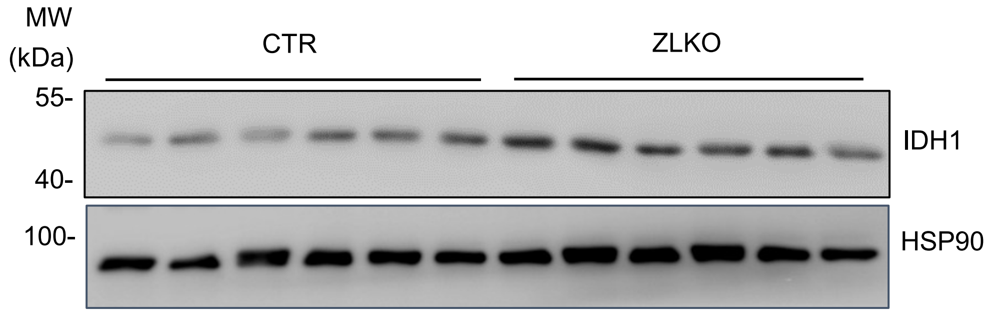

Full unedited blot for Figure 4O

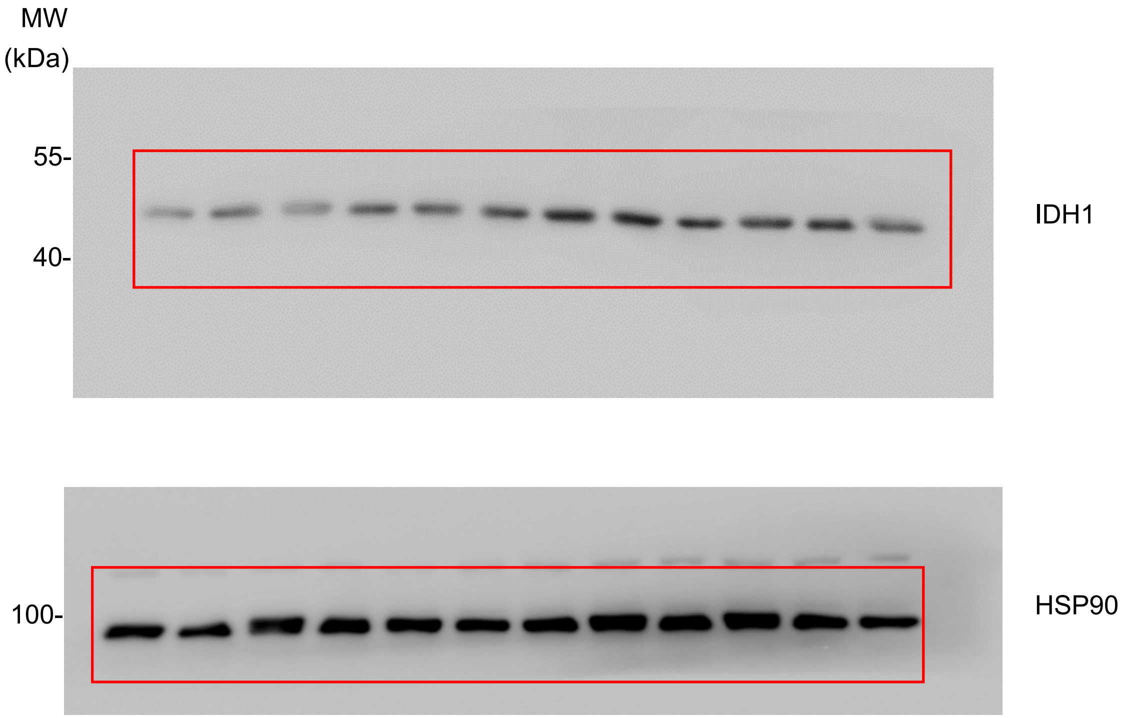

Figure 4Q

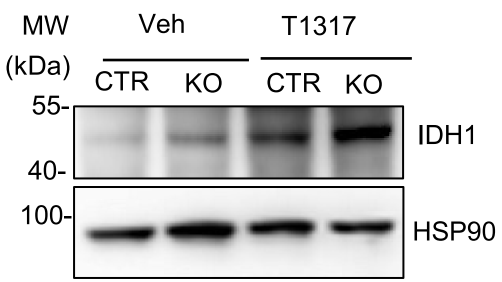

Full unedited blot for Figure 4Q

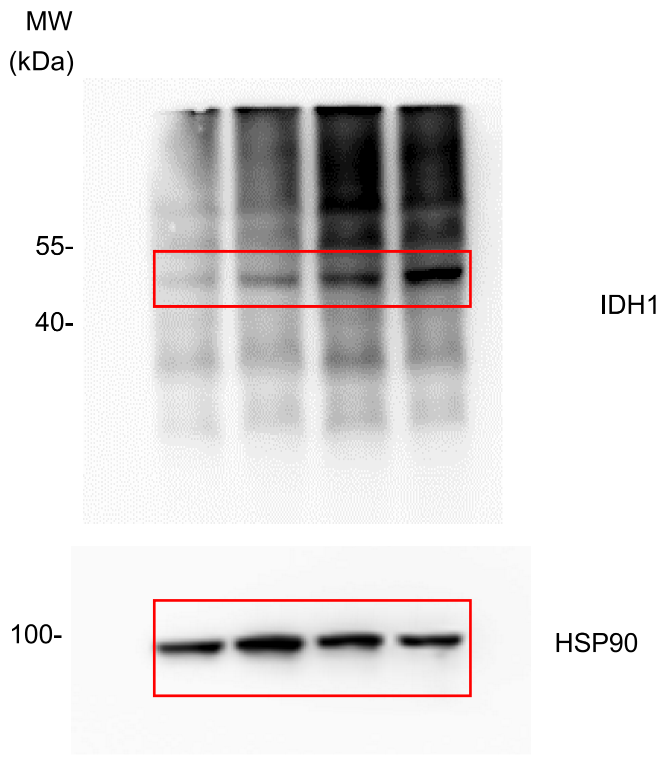

Figure 6F

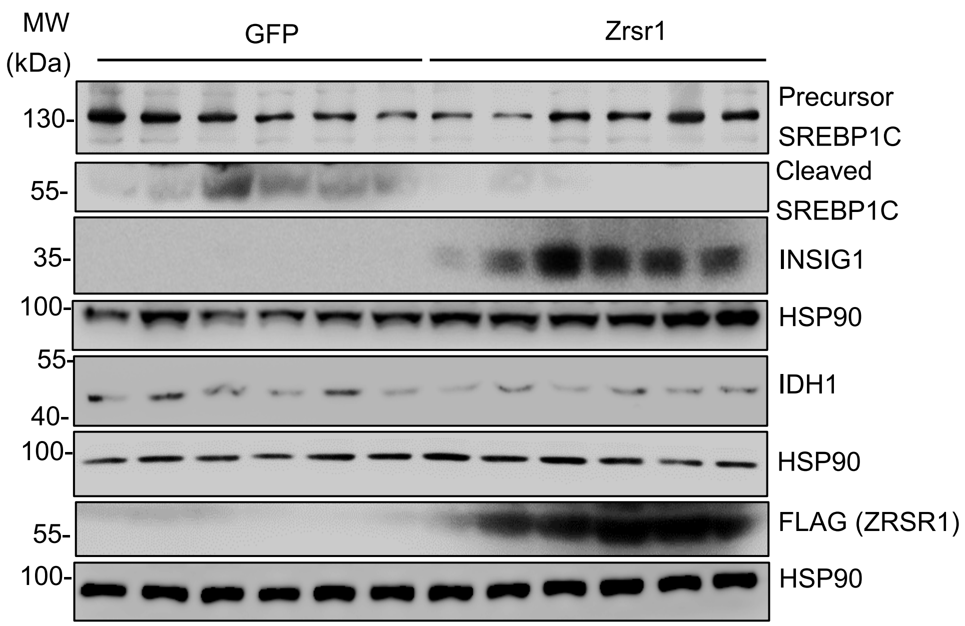

Full unedited blot for Figure 6F

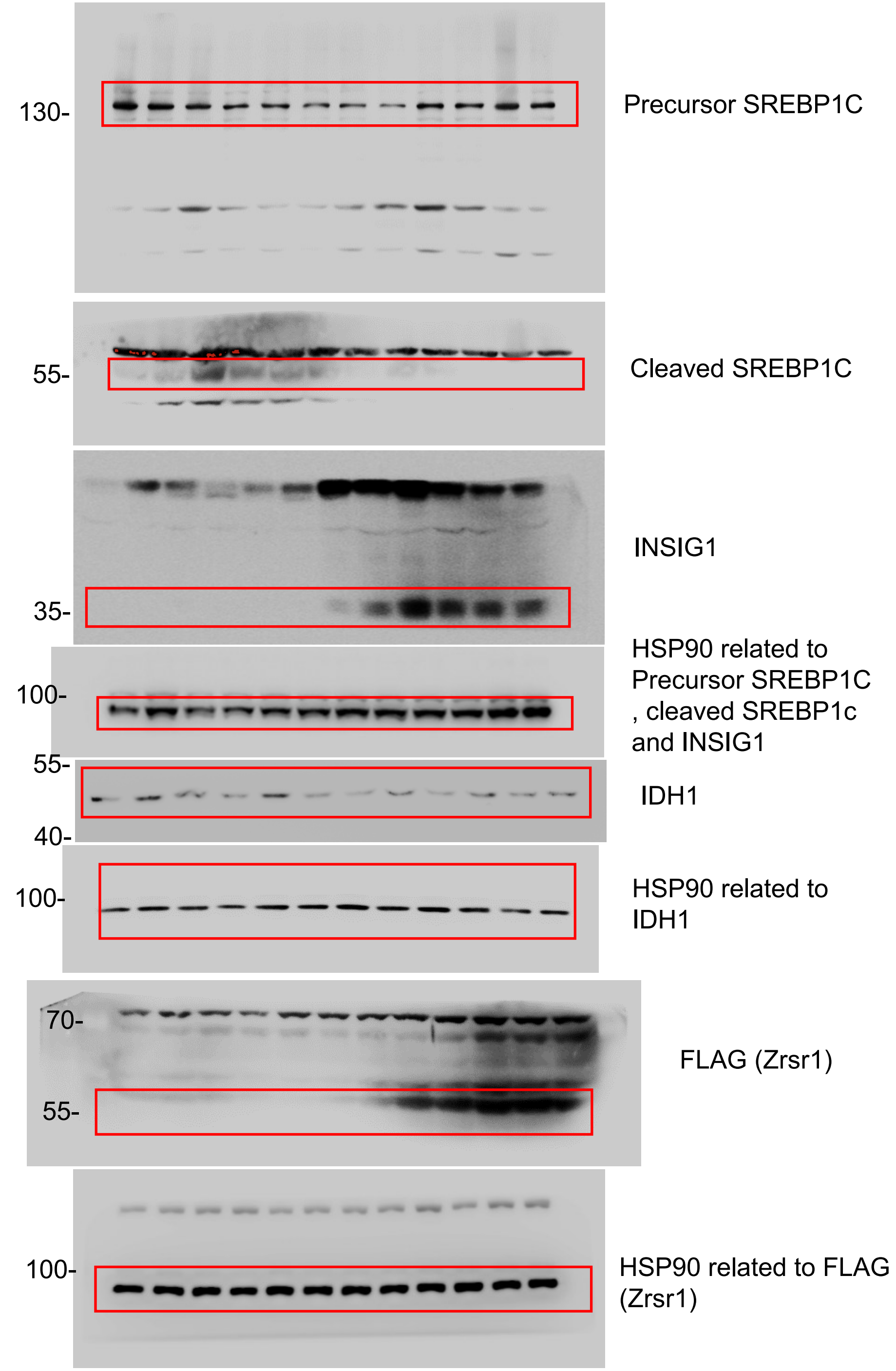

Full unedited gel for Supplement Figure 1

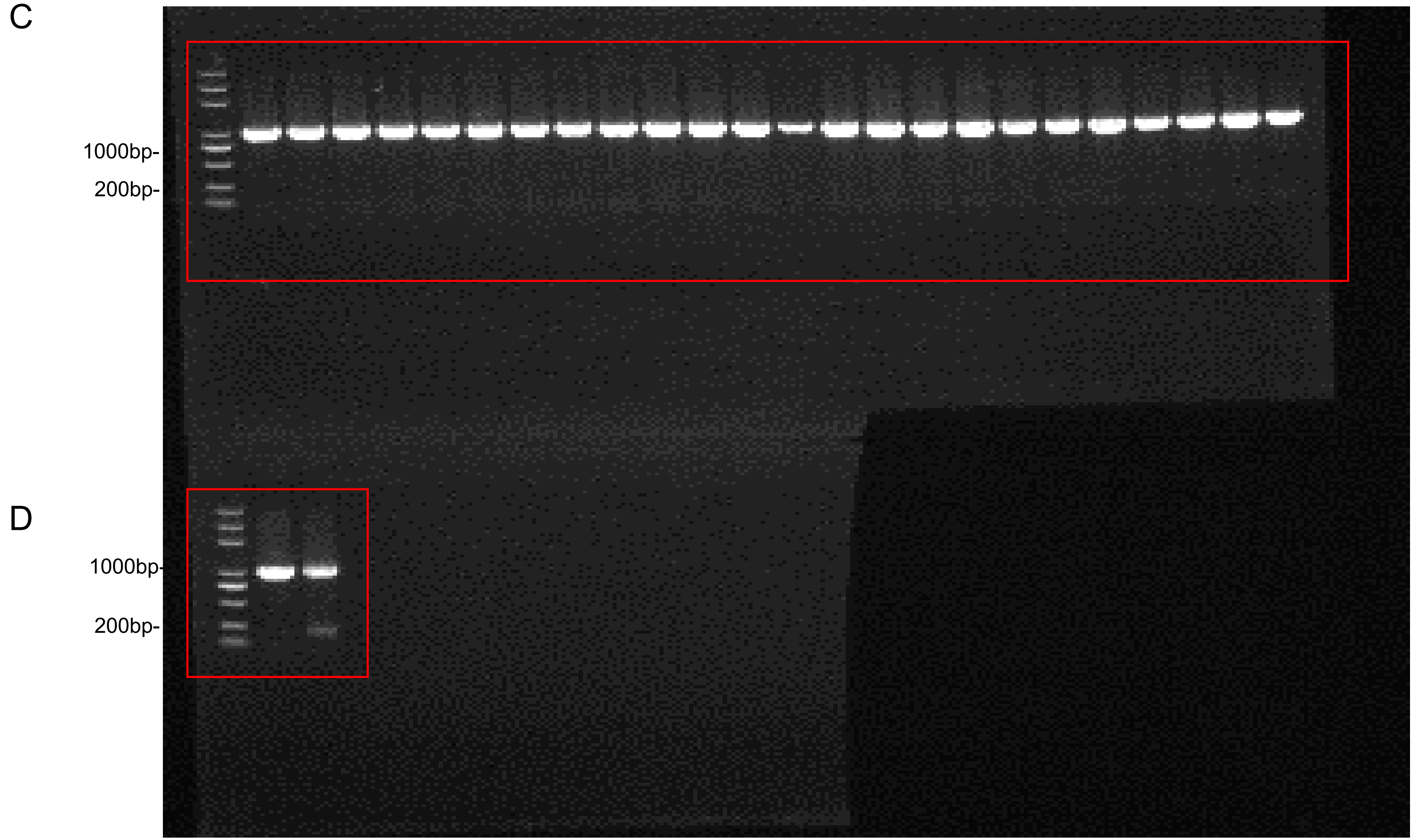

Full unedited gel for Supplement Figure 1

E

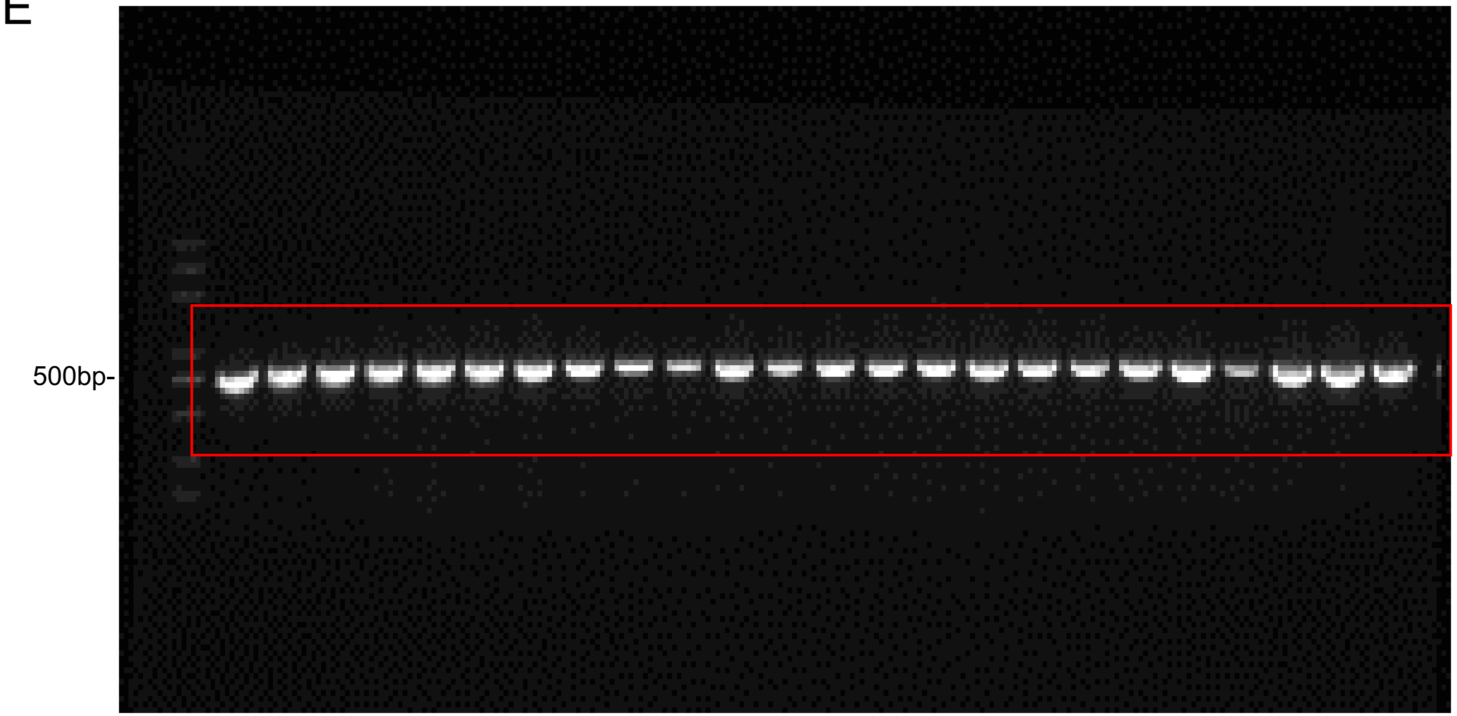

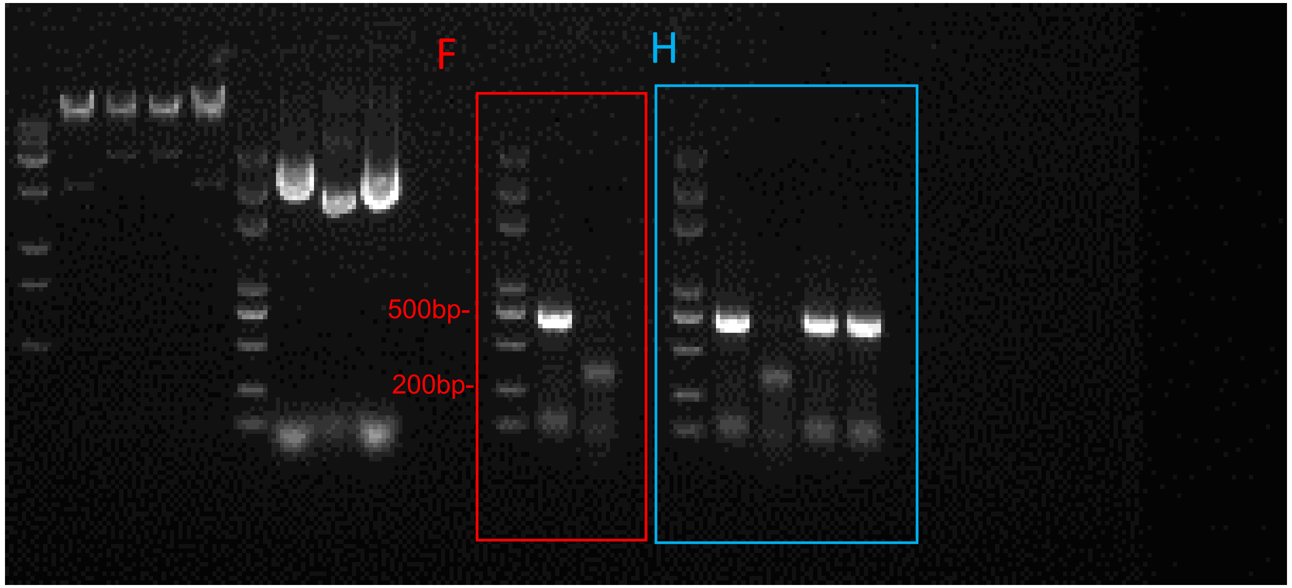

G

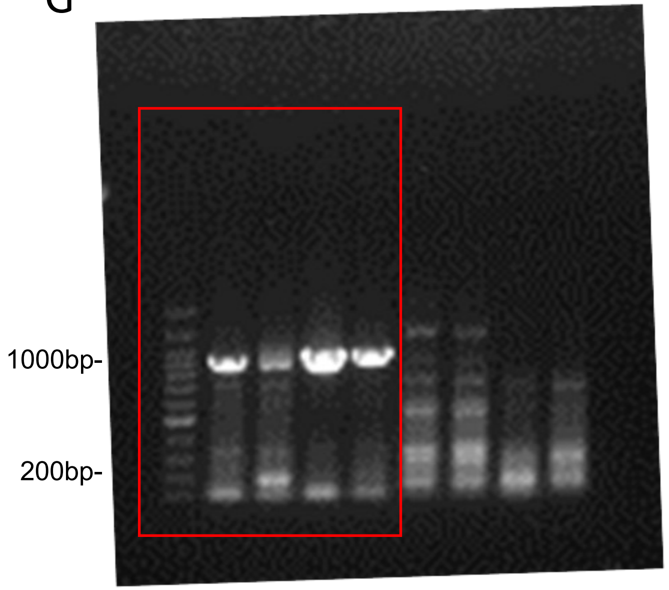

Figure S5H

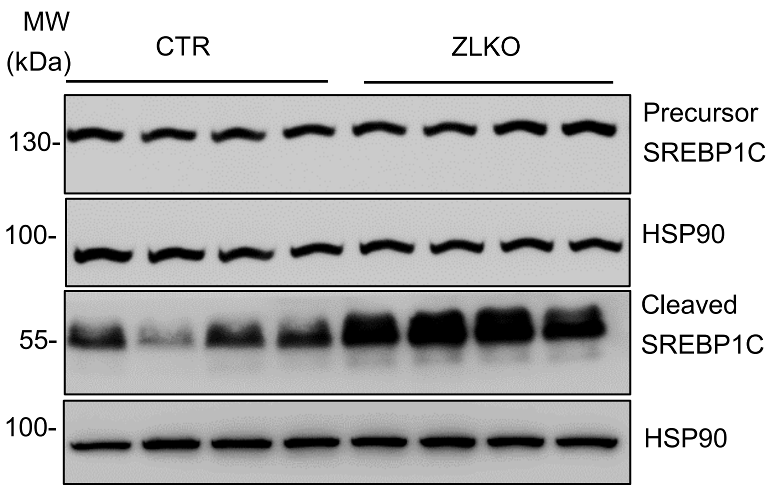

Full unedited blot for Figure S5H

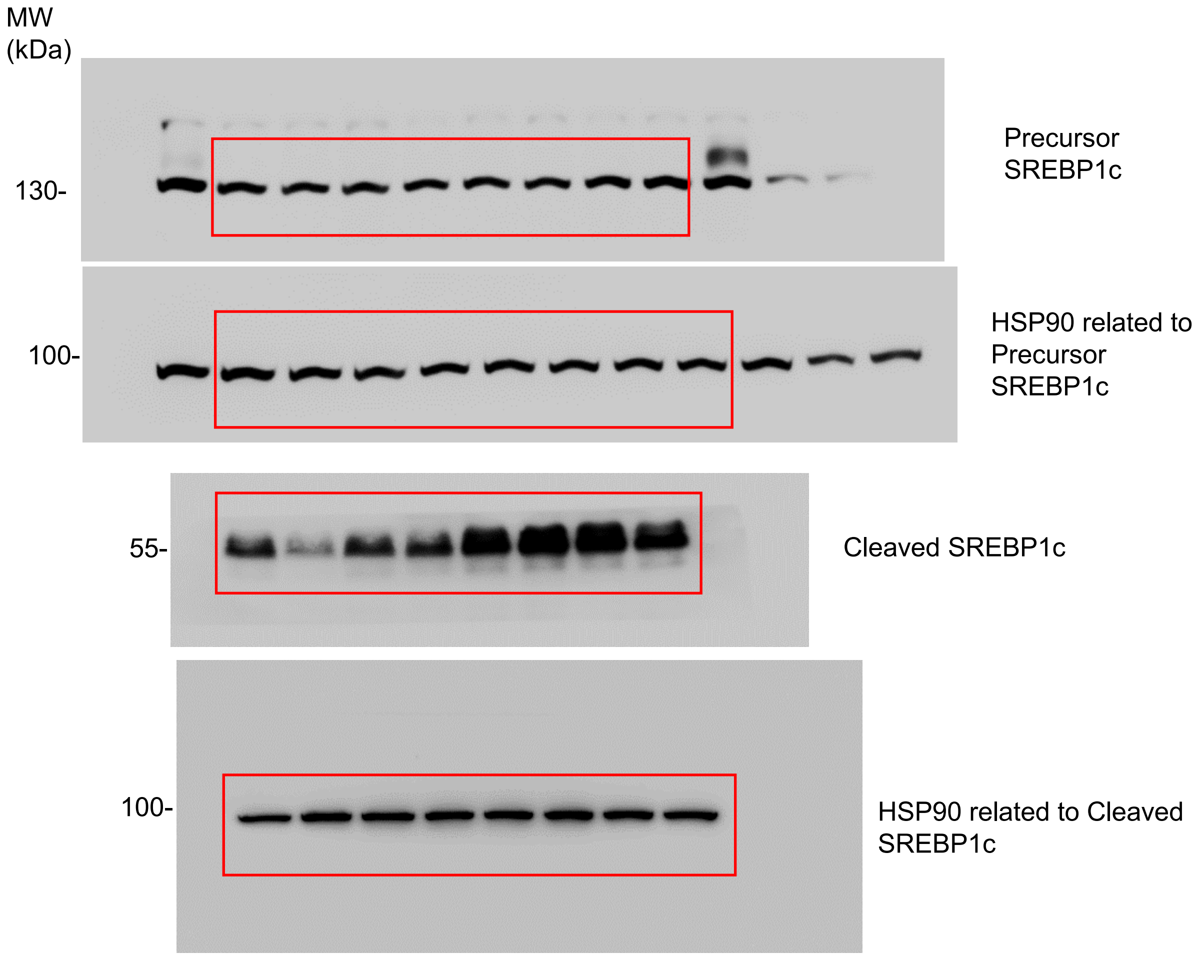

Figure S7K

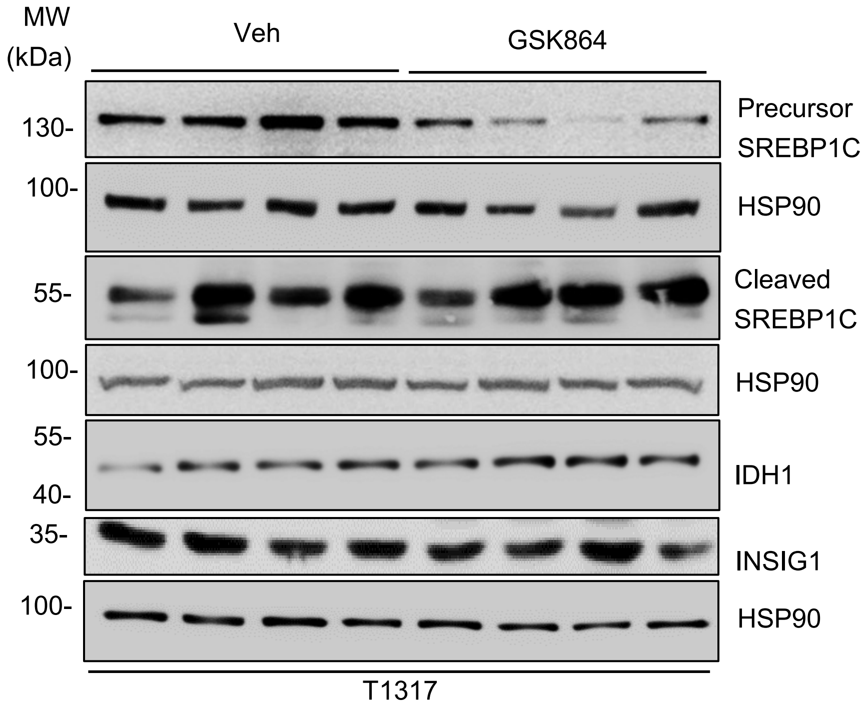

Full unedited blot for Figure S7K

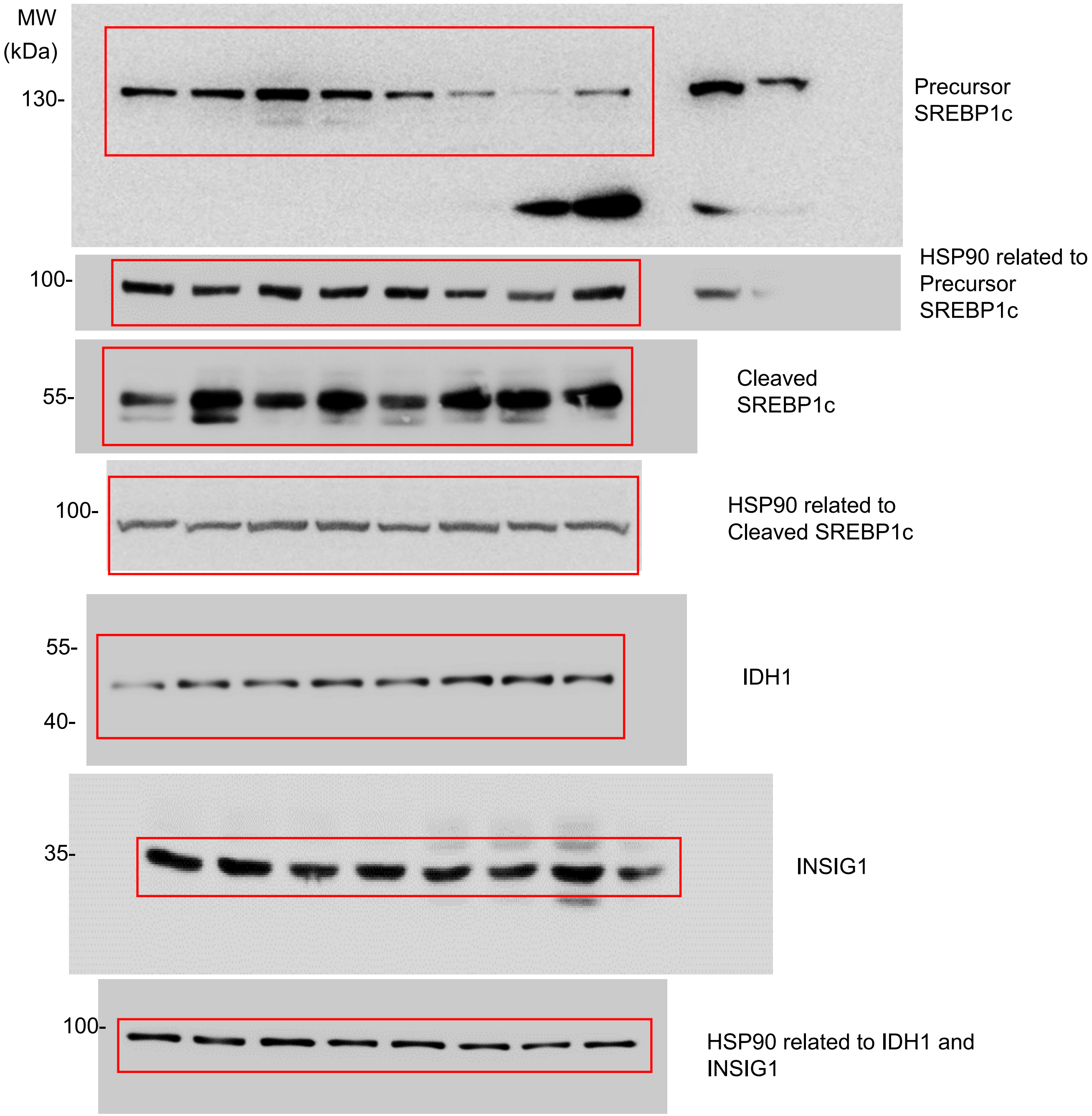

Supplement: Unedited blot and gel images [file jci-135-186478-s232.pdf]
